# Coevolution between simple sequence repeats (SSRs) and virus genome size

**DOI:** 10.1186/1471-2164-13-435

**Published:** 2012-08-30

**Authors:** Xiangyan Zhao, Yonglei Tian, Ronghua Yang, Haiping Feng, Qingjian Ouyang, You Tian, Zhongyang Tan, Mingfu Li, Yile Niu, Jianhui Jiang, Guoli Shen, Ruqin Yu

**Affiliations:** 1 Chinese Academy of Inspection and Quarantine, Beijing, 100029, China; 2 College of Biology, State Key Laboratory for Chemo/Biosensing and Chemometrics, Hunan University, Changsha, 410082, China; 3 College of Environmental Science and Engineering, Hunan University, Changsha, 410082, China

**Keywords:** Simple sequence repeats, Microsatellite, Genome size, Virus genomes, Evolution

## Abstract

**Background:**

Relationship between the level of repetitiveness in genomic sequence and genome size has been investigated by making use of complete prokaryotic and eukaryotic genomes, but relevant studies have been rarely made in virus genomes.

**Results:**

In this study, a total of 257 viruses were examined, which cover 90% of genera. The results showed that simple sequence repeats (SSRs) is strongly, positively and significantly correlated with genome size. Certain repeat class is distributed in a certain range of genome sequence length. Mono-, di- and tri- repeats are widely distributed in all virus genomes, tetra- SSRs as a common component consist in genomes which more than 100 kb in size; in the range of genome < 100 kb, genomes containing penta- and hexa- SSRs are not more than 50%. Principal components analysis (PCA) indicated that dinucleotide repeat affects the differences of SSRs most strongly among virus genomes. Results showed that SSRs tend to accumulate in larger virus genomes; and the longer genome sequence, the longer repeat units.

**Conclusions:**

We conducted this research standing on the height of the whole virus. We concluded that genome size is an important factor in affecting the occurrence of SSRs; hosts are also responsible for the variances of SSRs content to a certain degree.

## Background

Viruses are small infectious agents, which are found wherever there is a life and have probably existed since living cells first evolved [1,2]. There are millions of virus types [3]. Wherein, those virus species which have been reported were sorted into dsDNA, ssDNA, dsDNA-RT, ssRNA-RT, dsRNA, (−)ssRNA and (+)ssRNA viruses based on their genome types; they can also be sorted into algae, archaea, bacteria, fungi, invertebrates, plants, protozoa and vertebrates viruses based on the general host categories according to the ICTV (International Committee on the Taxonomy of Viruses) [4]. These viruses can infect all types of organisms including archaea, bacteria, plants and animals [5]. Many common human diseases are caused by viruses, such as common cold, influenza, chickenpox, cold scores, etc. In addition, many serious diseases such as ebola, AIDS, avian influenza and SARS are also caused by viruses. What's more, many genotypes of viruses are responsible for cancers, for example, human papillomavirus, hepatitis B virus, hepatitis C virus, Epstein-Barr virus, Kaposi's sarcoma-associated herpesvirus and human T-lymphotropic virus, and so on ( http://en.wikipedia.org/wiki/Virus). Though there are three main theories on the origin of virus: regressive, cellular and coevolution origin theory, it is still unclear how viruses originated because they do not like other organisms forming fossils [6,7]. So studying viruses via molecular information has been the most useful means in investigating how they arose and evolved [6,8-10]. Success of viral genome researches will promote our understandings and solutions of numerous problems, including their origin, evolution, infection mechanism, disease treatment, etc. The genome sizes (defined as haploid DNA content) of viruses vary greatly between species. The smallest viral genomes — the ssDNA circoviruses, family Circoviridae — code for only two proteins and have a genome size of only 2 kb; the largest — miniviruses have genome sizes of over 1.2 Mb and code for over one thousand proteins [11,12]. Two main mechanisms have been implicated in changes of genome size: one is the accumulation of transposable elements [13,14]; the other is the accumulation of tandemly repetitive sequences [15].

Simple sequence repeats (SSRs), also known as microsatellites, generally defined as simple sequences of 1–6 nucleotides that are repeated multiple times and are present in both coding and non-coding regions of the genome [16,17]. SSRs are ubiquitous and highly abundant in eukaryotic [18-21] and prokaryotic genomes [22,23]. DNA repeats are primarily expanded by three models: replication, repair and recombination [24]. Meiotic recombination plays a key role in the maintenance of sequence diversity in the human genome, and SSRs have been reported to be hot spots for recombination as well as sites for random integration [25,26]. Thus, alterations in SSRs lie at the center of DNA evolution and sequence diversity that drives adaptation; on the other hand, changes in repetitive sequences can result in deleterious effects on gene expression and function, leading to diseases [17]. The instability of SSRs was identified to be a pathway to lead to colorectal cancer [27]. It is now accepted that unstable maintenance of microsatellites occurs in about 15% of sporadic colorectal cancers [28,29]. Microsatellite instability is also frequently associated with other diseases such as ovarian cancers, malignant tumors of endometrium [30], small intestine [29], stomach [31], skin [32] and brain, etc. The features of microsatellite instability observed in bacteria, yeast, mice and man can provide general clues as to how genomes evolve and how certain instability could contribute to human disease [17]. Some pathogens use SSRs in a strategy that counteracts the host immune response by increasing the antigenic variance of the pathogen population [33].

Genome sequences with diverse lengths make it possible to investigate the relationship between genome size and accumulation of SSRs in all virus genera whose complete genome sequences have been reported. Therefore, scatter plots and regression analysis were performed to survey the correlation between repetitiveness (SSRs occurrence as well as SSRs length) and genome size. Distributions of different repeat classes were also surveyed among virus genomes of various sizes. While, relative abundance and relative density were examined to make the SSRs comparison parallel among differently sized species genomes; principal component analysis (PCA) was designed to investigate which repeat class(es) made a greater contribution to the variance among virus species as well as the relationships between repeat classes.

## Methods

### Genome sequences

The Eighth Report of ICTV (International Committee on Taxonomy of Viruses) provided information on 3 orders, 73 families, 9 subfamilies, 287 genera and 1938 virus species [4]; wherein 257 genera have been reported on complete genome sequences on NCBI and one typical species was identified as the representative for each genus according to the Listing in Taxonomic Order ( http://ictvdb.bio-mirror.cn/Ictv/index.htm). Therefore, the 257 genome sequences were selected as samples for the analysis of relationship between SSRs distribution and genome size in the level of the whole virus. All the genome sequences were downloaded in both Genbank and FASTA formats from the NCBI ( ftp://ncbi.nlm.nih.gov/genbank/). Sequences obtained include DNA and RNA, so both T and U bases were represented with T. Some genomes were segmented, multipartite and consist of two or more segments with various sizes ( Additional file 1).

### SSRs extraction

SSRs were identified and localized using the software SSR Identification Tool (SSRIT), which identifies perfect di-, tri-, tetra-, penta- and hexanucleotide repeats. We have considered only those repeats, wherein the motif was repeated more than 3 times for further analysis. Mononucleotide repeats (with a repeat length of 6 nt) were identified using the tool IMEX (Imperfect Microsatellite Extractor), which can extract perfect microsatellites as well as imperfect microsatellites. Here we presented the data for all perfect repeat types. No distinctions between the occurrence of repeats in coding and noncoding regions were made, the rationale for this decision was that the coding regions often account for the large proportion (mean value approximately 90%); while the sequences of noncoding regions are usually very short; moreover, the overlap phenomenon is very common in virus genomes, and many of the details were presented in Additional file 1.

### Relative abundance and relative density

These total numbers have been normalized by using relative abundance and relative density of SSRs to allow the comparisons to be parallel among genome sequences with different sizes. Relative abundance was calculated by dividing the number of SSRs by kilo base pair (kb) of sequences; and relative density (bp/kb) was calculated by dividing the total sequences analyzed (kb) by the number of base pairs of sequence contributed by each SSR.

### PCA

Principal Components Analysis (PCA) is a well known statistical technique which has wide ranging applications. The main goal of PCA is to reduce the dimensionality by decomposing the total variances observed in an original data set. That is to say, we use PCA method to transform a set of original variables into a set of new and uncorrelated variables. The mathematic principle of PCA method lies in coordinate conversion. Consequently, PC (principal component) is a linear combination of the original variables.

**Mathematical model.** If the sample size is*n*, and each sample has *P* observed index (*X*1,  *X*2, …, *Xp*), we can get the following matrix of the original dataset:

$$X=\left[\begin{array}{cccc} x_{11} & x_{12} & \ldots & x_{1p}\\ x_{21} & x_{22} & \ldots & x_{2p}\\ \vdots & \vdots & & \vdots \\ x_{n1} & x_{n2} & \ldots & x_{\mathrm{np}} \end{array}\right]=\left(X_{1},X_{2},\ldots ,X_{P}\right)$$

Wherein, $X_{i}=\left[\begin{array}{c} x_{1i}\\ x_{2i}\\ \vdots \\ x_{\mathrm{ni}} \end{array}\right],i=1,2,\ldots ,p\text{.}$

Making linear combinations using the *p* variables (*X*1,  *X*2, …, *Xp*) of the original data matrix *X*:

$$Y=\{\begin{array}{l} Y_{1}=e_{11}X_{1}+e_{21}X_{2}+\ldots +e_{p1}X_{p}\\ Y_{2}=e_{12}X_{1}+e_{22}X_{2}+\ldots +e_{p2}X_{p}\\ \ldots \\ Y_{p}=e_{1p}X_{1}+e_{2p}X_{2}+\ldots +e_{\mathrm{pp}}X_{p} \end{array}$$

Hence, $Y_{i}=e_{1i}X_{1}+e_{2i}X_{2}+\ldots +e_{\mathrm{pi}}X_{p},i=1,2,\ldots ,p$

Here, *Yi* is the principal component, but it must meet the following conditions: (1) ${e_{1i}}^{2}+{e_{2i}}^{2}+\ldots +{e_{\mathrm{pi}}}^{2}=1,\left(i=1,2,\ldots ,p\right)$; (2) there is no correlation between *Yi* and *Yj* ( i≠j,i,j=1,2,…,p); (3) the variance of *Yi* is the maximum during Yi,Yi+1,…,Yp; (4) $Var\left(Y1\right)+Var\left(Y2\right)+\ldots +Var\left(Yp\right)=Var\left(X1\right)+Var\left(X2\right)+\ldots +Var\left(Xp\right)$

**Figure 1:**
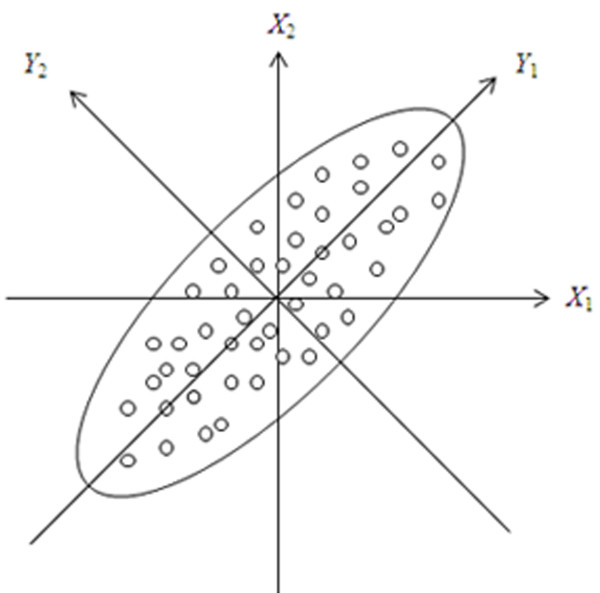
**Geometric meaning of PCA explained by using bivariate normally distributed variables.** Scatters of sample are distributed in the shape of ellipse roughly, then orthogonally rotate the original plane rectangular coordinates composed of *X*_*1*_ and *X*_*2*_with an angle θ. By now, two original correlated variables(*X*_*1*_*, X*_*2*_)were transformed into two integrated and uncorrelated variables (*Y*_*1*,_*Y*_*2*_). Because the variance of the original variables is greater in *Y*_*1*_ axis than in *Y*_*2*_ axis, so the minimum of information will be lost if integrated variable *Y*_*1*_ is used for replacing all original variables. Hence,*Y*_*1*_ is defined as the first principal component; in contrast, variance of variables is smaller in *Y*_*2*_ axis, and it can explain minor information relative to *Y*_*1*_, so*Y*_*2*_ is called the second principal component.

**Geometric meaning.** Supposing that the sample contains *n* individuals, each individual has two variables *X*1,  *X*2, and in addition, variables subject to the normal distribution. That is, we discuss the geometric meaning of PCA by using bivariate normally distributed variables. Therefore, scatters of sample are roughly distributed in the shape of ellipse (Figure 1). Then orthogonally rotate the original plane rectangular coordinates composed of *X*1 and *X*2 with an angle *θ*, thus, two original correlated variables (*X*1,  *X*2) were transformed into two integrated and uncorrelated variables (*Y*1,  *X*2), and the correlation between the original and new axes is as following:

$$\left\{ \begin{array}{l} F_{1}=X_{1}\cos\theta +X_{2}\sin\theta \\ F_{2}=-X_{1}\sin\theta +X_{2}\cos\theta \end{array}\right.$$

Because the variance of the original variables is greater in *Y*1 axis than in *Y*2 axis, so a minimum of information will be lost if integrated variable *Y*1 is used for replacing all original variables. Hence, *Y*1 is defined as the first principal component; in contrast, the variance of variables is smaller in *Y*2 axis, and it can explain minor information relative to *Y*1, so *Y*2 is called the second principal component.

## Results

To obtain an expansive and unbiased data set, all virus genera with complete genome sequences reported on NCBI were scanned for SSRs analysis; wherein, one typical species was selected as the representative for each genus according to the ICTVdb ( http://ictvdb.bio-mirror.cn/Ictv/index.htm). Therefore, we analyzed perfect SSRs over 6 bp long, from the 257 completely sequenced virus genomes. While, the genome size varies widely, ranging from 1682 bp (S170-(−)ssRNA-31, *Hepatitis delta virus*, NC_001653) to 407339 bp (S42-dsDNA-42, *Emiliania huxleyi virus 86*, NC_007346) ( Additional file 1).

### Relationship between SSRs and genome size

**Figure 2:**
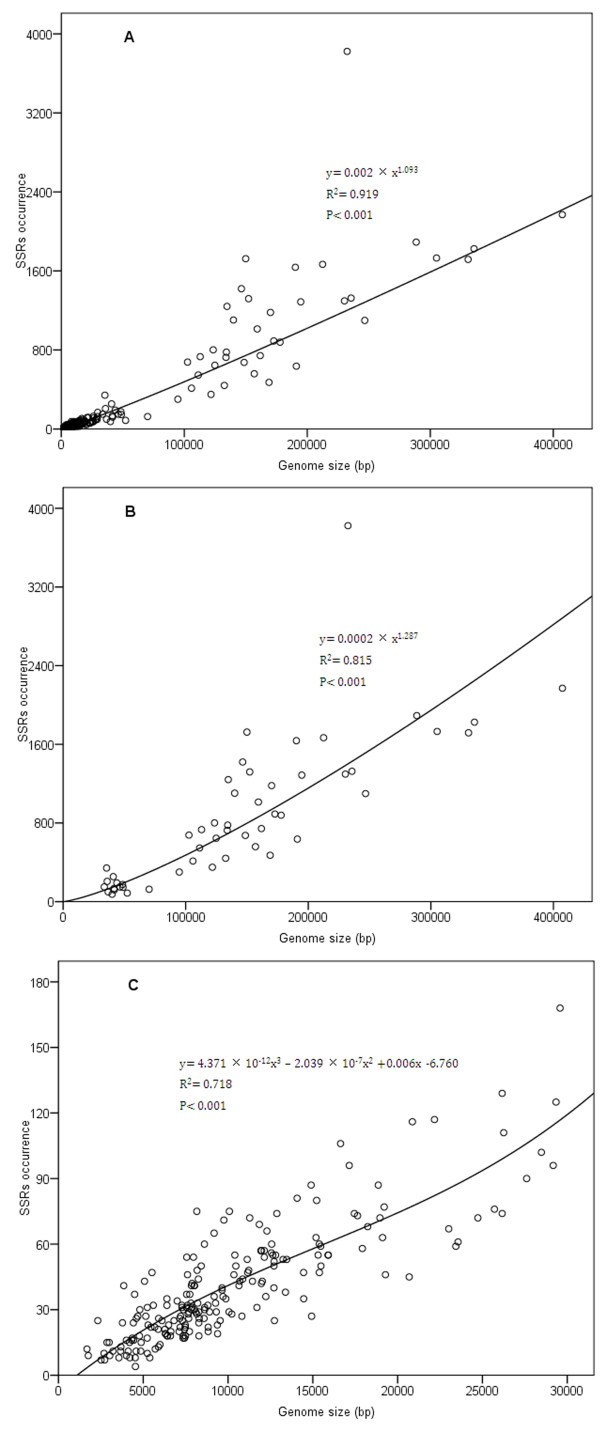
**Regression analysis of relationship between SSRs occurrence and genome size.** (**A**) Scatter plot of SSRs occurrences in all analyzed virus genomes. (**B**) Scatter plot of SSRs occurrences in analyzed virus genomes > 30000 bp. (**C**) Scatter plot of SSRs occurrences in analyzed virus genomes < 30000 bp.

**Figure 3:**
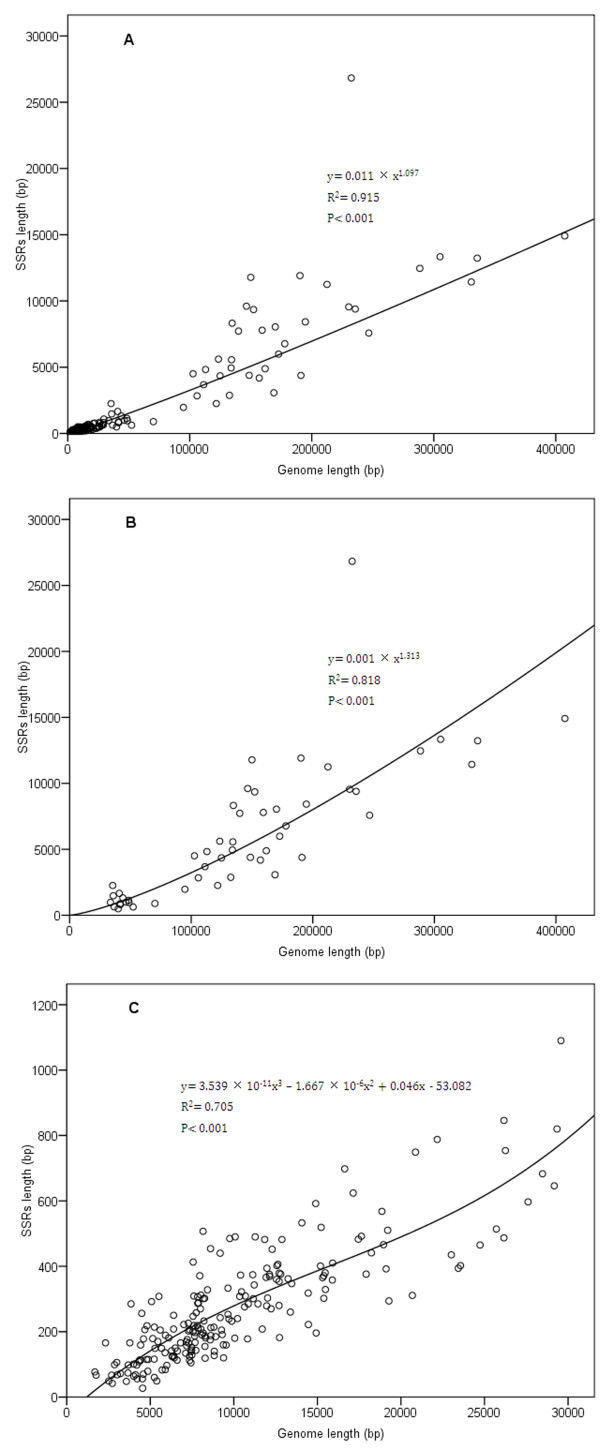
Regression analysis of relationship between SSRs length and genome size.

We constructed two sets of scatter plots and then performed regression analysis of SSRs (occurrence and length) versus complete genome size for all analyzed viruses to examine the relationship between SSRs and genome size. Above all, scatter plots were made, in which, genome size was taken as an independent variable, and all analyzed data were split into two groups (genome > 30000 bp and ≤ 30000 bp) to make the scatters and curves natural and visible (Figures 2, 3); and then 10 curves (linear, logarithmic, inverse, quadratic, cubic, compound, power, S, growth and exponential) were fitted according to their respective mathematical models by using the software SPSS 17.0. Parameter estimates and visual inspection showed that goodness fit of data varies greatly to different models; nevertheless, curves with the best goodness of fit were picked out for correlation analysis between SSRs (occurrence and length) and genome size (Figures 2, 3). The number of repeat arrays varies from 4 in *Nodamura virus* genome (S206-(+)ssRNA-36) to 3823 in *Amsacta moorei entomopoxvirus 'L'* genome (S33-dsDNA-33) ( Additional file 2). The power function model provides the best fitted values towards all studied SSRs occurrence and genome size by regression analysis, and results display a very strong and significant positive relationship between the occurrence of SSRs and genome size clearly (R^2^ = 0.919, P < 0.001) (Figure 2A). Power function and cubic model best fit for the data of genome > 30000 bp and ≤ 30000 bp group, respectively (Figure 2B,C). Clearly, the SSRs occurrence is strongly, significantly and positively related to the genome size in both genome > 30000 bp (R^2^ = 0.815, P < 0.001) and ≤ 30000 bp (R^2^ = 0.718, P < 0.001) group. Especially in the group of genome ≤ 30000 bp, the values of SSR occurrences fluctuate with a relatively narrow range. An exceptional case is worth noting. One point of the scatter plot locating far above the fitted curve represents the value of SSRs in *Amsacta moorei entomopoxvirus 'L'* genome (S33-dsDNA-33, NC_002520) with the size of 232392 bp, in which the SSRs occurrence is a total of 3823, far more than SSRs in any other analyzed virus genome.

The length of SSRs varies from 27 bp in *Nodamura virus* genome (S206-(+)ssRNA-36) to 26829 bp in *Amsacta moorei entomopoxvirus 'L'* genome (S33-dsDNA-33); and the percentage of SSRs varies from 0.59% in *Nodamura virus* genome (S206-(+)ssRNA-36) to 11.54% in *Amsacta moorei entomopoxvirus 'L'* genome (S33-dsDNA-33) ( Additional file 3). Similarly, we investigated the correlation between SSRs length and genome size. Figure 3 showed that the distribution of SSRs length is similar to the SSRs occurrence in differently-sized genomes, and it indicated that SSRs length is also significantly and positively correlated with the genome size to all analyzed data (R^2^ = 0.915, P < 0.001), to genome >30000 bp group (R^2^ = 0.818, P < 0.001) and to genome ≤ 30000 bp (R^2^ = 0.705, P < 0.001) group. Likewise, *Amsacta moorei entomopoxvirus 'L'* genome (S33-dsDNA-33, NC_002520) shows features out of the ordinary, with the total SSRs length of 26829 bp and SSRs percentage of 11.54%, occupying the number-one spot in length and percentage of SSRs among all analyzed virus genomes. Except that, other points float up and down the curve with a small range (Figure 3). The above results indicated that genome size is an important factor in affecting repetitiveness of microsatellites in viruses.

### Relationship between repeat class and genome size

We surveyed the distribution of different SSR classes in virus genomes to investigate the relationship between repeat classes (mono-, di-, tri-, tetra-, penta- and hexa-) and genome sequence length. The data of genome size < 2 kb group are not in our consideration here, because too small sample sizes lead to statistical insignificance. Data presents such a trend that, for the same repeat class, the ratio of genomes with corresponding SSRs to all genomes increases with the genome sequence growing, although the genome distribution is uneven among different genome ranges (Table 1). For example, the ratio of genomes with hexanucleotide SSRs is 0 in group of 2 ~ 5 kb, and it is 1.1% in 5 ~ 10 kb, 2.6% in 10 ~ 20 kb, 6.7% in 30 ~ 100 kb and 63.9% in > 100 kb group, respectively. For the same range of genome sizes, tendency seems to be that the ratio decreases with the increase of the length of repeat unit. For example, in the genome range of 10 ~ 30 kb, the ratio is 100% (mono-), 100% (di-), 98.7% (tri-), 19.2% (tetra-), 2.6% (penta-) and 2.6% (hexa-), respectively. Observed value per virus genome showed a rising trend with the increase of the genome sequence. Additionally, long repeat units such as penta- and hexa- SSRs were rarely, or even not, observed in small genomes, and certain repeat unit class distributed in genomes with a certain range of sequence length. All mono- and di- repeats were observed in analyzed genomes except *Duck hepatitis B virus* (S103-dsDNA-RT-2), *Cryphonectria parasitica mitovirus 1* (S174-(+)ssRNA-4) and *Nodamura virus* (S206-(+)ssRNA-36) in which mono- repeats were not found; tri- repeats seemed to widely distribute in all virus genomes; and tetra- SSRs, as a common component, consist in genomes with size more than 100 kb (94.4% of the virus genomes contain tetra- in group of genome >100 kb); In contrast, it is rarely observed in genomes with size < 100 kb; and genomes containing penta- and hexa- SSRs are not more than 50% in < 100 kb group. Moreover, the number of tetra-, penta- and hexa- SSRs is very small in genome range of < 100 kb (Table 1). Results indicated that the correlation is strong between length of repeat unit and genome size. The longer the genome sequence, the longer repeat units. For the same repeat unit class such as mononucleotide SSRs, the number of SSRs increases with the genome length increasing. It confirmed a preference that SSRs tend to accumulate in larger virus genomes.

**Table 1:** Distribution of repeat classes in different ranges of genome size

**Range (kb)**	**Geno. No.**^**1**^	**Mono-**			**Di-**			**Tri-**			**Tetra-**			**Penta-**			**Hexa-**		
		**G .N. R.**^**2**^	**%**^**3**^	**O. V.**^**4**^	**G .N. R.**	**%**	**O. V.**	**G .N. R.**	**%**	**O. V.**	**G .N. R.**	**%**	**O. V.**	**G .N. R.**	**%**	**O. V.**	**G .N. R.**	**%**	**O. V.**
~ 2	2	2	100	10	2	100	7	2	100	3	1	50	1	0	0	0	0	0	0
2 ~ 5	32	29	90.6	162	32	100	268	28	87.5	81	2	6.3	4	0	0	0	0	0	0
5 ~ 10	94	94	100	851	94	100	1585	90	95.7	363	9	9.6	11	1	1.1	1	1	1.1	1
10 ~ 30	78	78	100	1482	78	100	2822	77	98.7	626	15	19.2	17	2	2.6	4	2	2.6	2
30 ~ 100	15	15	100	1020	15	100	1183	15	100	342	4	26.7	5	2	13.3	3	1	6.7	1
100 ~ 410	36	36	100	16009	36	100	19587	36	100	5440	34	94.4	236	19	52.8	40	23	63.9	121

^1^ Genome number, e.g., the number of genomes is 32 with the size of 2 ~ 5 kb; ^2^ Genome number with corresponding repeat class, e. g., there are 29 virus genomes from which mononucleotide SSRs were extracted in the genome range of 2 ~ 5 kb; ^3^ Ratio of G. N. R. to Geno. No.; ^4^ Observed value of corresponding SSRs, e. g., a total of 162 mononucleotide repeat motifs were extracted from the genome range of 2 ~ 5 kb.

### Relative abundance and relative density of SSRs

**Figure 4:**
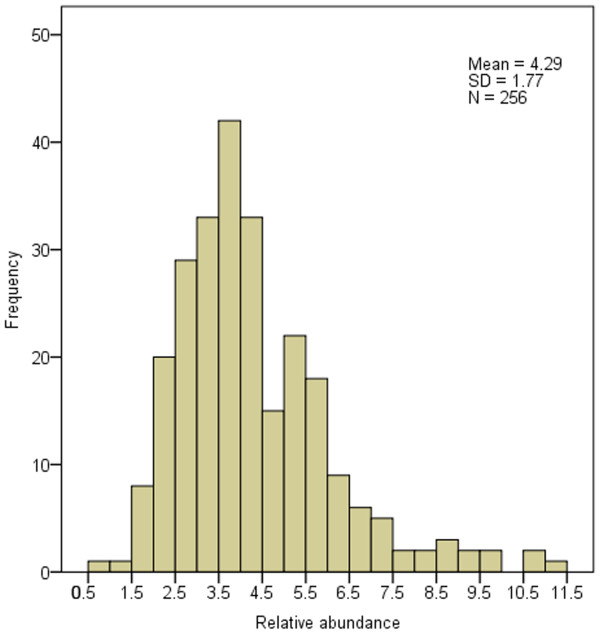
**Histogram of SSRs relative abundance.** The horizontal axis represents the relative abundance of SSRs in all analyzed virus genomes. The vertical axis represents the genome frequency with the corresponding SSRs relative abundance. The definition of relative abundance of SSRs can be seen in MATERIAL AND METHODS.

**Figure 5:**
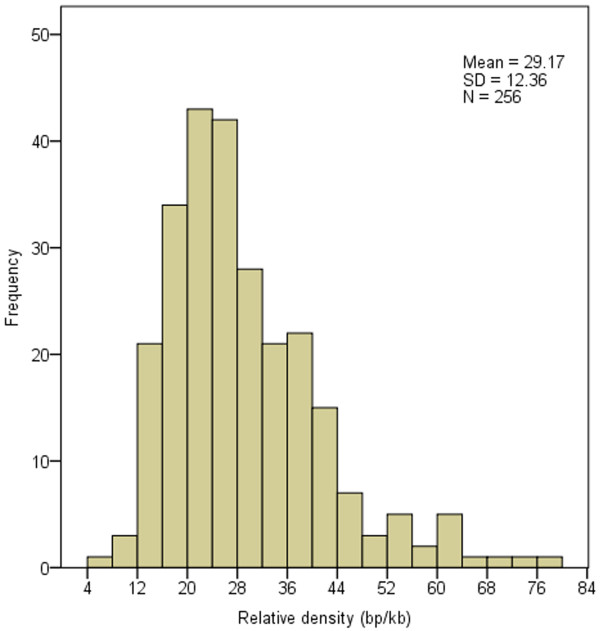
Histogram of SSRs relative density.

Because of the irregular sizes of analyzed virus genomes, we calculated the relative abundance and relative density of SSRs to make the comparison of SSRs abundance parallel among differently-sized genomes. Frequency of virus genomes with the SSRs relative abundance of 2.0 ~ 6.0 is quite high with the value of 212 (82.8% of all analyzed viruses). Wherein, 108 genomes (42.2% of all analyzed viruses) were found to have the SSRs relative abundance of 3.0 ~ 4.5. However, genomes with the SSRs relative abundance of < 2.0 and > 6.0 are relatively fewer (with the total number of 44, accounting for 17.2% of all analyzed viruses) (Figure 4, Additional file 4). Paralleling, frequency of genomes is relatively high in the SSRs relative density range of 12 ~ 44 bp/kb with the genome number of 226 (88.3% of all analyzed viruses), and 147 genomes (57.4%) have the SSRs relative density among 16 ~ 32 bp/kb; moreover, 85 genomes (33.2%) have the SSRs density of 20 ~ 28 bp/kb (Figure 5, Additional file 5). The relationship between SSRs relative abundance, relative density and genome size were investigated respectively. Scatter plots showed that the correlations between the SSRs relative abundance and genome size and between the relative density and genome size are quite weak ( Additional file 6, Additional file 7). The results indicated that the genome size has slightly affected the relative abundance and relative density of SSRs in virus genomes. Chen et al. [34] also found that the relative abundance and relative density of SSRs were not significantly related to genome size. On the contrary, SSRs are distributed in the virus genomes with a certain proportion.

### PCA applying to SSRs study

PCA was used to examine which factor(s) primarily lead(s) to differences in SSRs abundance among the virus species. The sample with the size of 257 (n = 257 virus genomes) contains 6 variables (p = 6, including the percentages of mono-, di-, tri-, tetra-, penta-, hexa-, respectively). Di- SSRs is the most and hexa- SSRs is the least on average, but the standard deviation is very large for each repeat unit class among the virus genomes ( Additional file 8). Even so, correlation is still strong and extremely significant between the original variables ( Additional file 9). The results showed that the two principal components with eigenvalues of 4.041 and 0.811 together can account for 80.869% of all differences of SSRs abundance among viruses. Wherein, the first component can account for 67.351% and the second 13.518% of all variances, respectively. Other components played a less important role in explaining the differences of SSRs abundance among virus genomes. The comparison of the parameters' coefficients for the first and second components showed that the first component has a major loading on the difference of SSRs during analyzing genomes (Table 2). The results indicated that the SSRs differences among virus genomes are mainly due to the following parameters: mono-, di-, tri- and tetra-. Wherein, the variable of di- affects the differences of SSRs among virus genomes most strongly with the loading of 0.939, followed by tri-, mono- and tetra-. In this component, penta- and hexa- played relative minor role in explaining the differences of SSRs among virus genomes. In the second component, hexa- with high positive coefficient and tetra-, penta- with negative coefficients showed different effects on SSRs abundance. In this component, hexa- played the most important role in explaining differences of SSRs abundance. Overall, the results of PCA indicated that di- affected the SSRs variances among virus genomes most strongly, followed by tri-, mono- and tetra-; and then by hexa-; penta- played the weakest role in influencing the variances of SSRs abundance among viruses.

**Table 2:** Loadings of variables on the first two extracted principal components

**Variable**	**PC 1**	**PC 2**
Mono-	**0.885**	−0.100
Di-	**0.939**	−0.036
Tri-	**0.892**	−0.035
Tetra-	**0.875**	−0.138
Penta-	0.752	−0.206
Hexa-	0.500	**0.859**
Eigenvalue	4.041	0.811
% of Variance	67.351	13.518
Equation	Y_1_ = 0.440X_1_ + 0.467X_2_ + 0.444X_3_ + 0.435X_4_ + 0.374X_5_ + 0.248X_6_	Y_2_ = −0.111X_1_-0.040X_2_-0.038X_3_-0.153X_4_-0.229X_5_ + 0.953X_6_
Cumulative %	80.869	
KMO Measure	0.866	
Bartlett's Test	< 0.001 (df = 15)	
Scree Test	Y	
Analyzed No.	257	

All results of Kaiser-Meyer-Olkin (KMO), Bartlett's and scree test indicated that it is significantly meaningful to analyze our data using PCA (Table 2). The KMO measure with the value of 0.866 is close to 1, and Bartlett's test (< 0.001) approximates to 0, and scree plot displays the "cliff" and the "screes" vividly ( Additional file 10). Moreover, the correlation is strong between the original variables ( Additional file 9).

### Preference of SSRs

SSRs vary greatly in repeat classes and motifs among analyzed virus genomes (Table 3, Additional file 11, Additional file 12, Additional file 13 and Additional file 14). Dinucleotide SSRs accounts for the largest proportion of 48.68% in all repeat classes, followed by mono- (37.36%) and trinucleotide SSRs (13.11%). Both A and T mono- SSRs are much more than C and G SSRs, and they make up about 16.38%, 15.54%, 2.74% and 2.69% of all SSRs in analyzed viruses respectively. AT/TA SSRs predominate in dinucleotide repeats with the proportion of 17.27%, and it is slightly more than A and T mono- SSRs (16.38%, 15.54%); other di- repeat motifs are neck and neck in occurrence, but they are all higher than C and G mono- SSRs (Table 3). Repeat motif group of AAT/ATA/ATT/TAA/TAT/TTA showed the highest percentage and AGT/ACT/CTA/GTA/TAC/TAG showed the lowest percentage in tri- SSRs. Tetra-, penta- and hexanucleotide SSRs are rare, accounting for 0.5% more or less. It's abnormal that penta- SSRs are less than hexa- SSRs with 0.09%, which is approximately only one third of hexa- SSRs. However, it is usually assumed that the longer repeat unit, the lower frequency it occurred. Repeat motifs differ greatly among different virus genomes (details in Additional file 11, Additional file 12, Additional 13, Additional file 14).

**Table 3:** Frequency of repeat motifs (group) in all analyzed virus genomes

**Repeat motif (group)**	**Frequency**	**Percentage (%)**
Mono-	19534	37.36
A	8564	16.38
C	1434	2.74
G	1408	2.69
T	8128	15.54
Di-	25452	48.68
AC/CA	3358	6.42
AG/GA	3124	5.97
AT/TA	9029	17.27
CG/GC	4094	7.83
CT/TC	2664	5.09
GT/GT	3183	6.09
Tri-	6855	13.11
AAT/ATA/ATT/TAA/TAT/TTA	1447	2.77
AAC/ACA/CAA/GTT/TGT/TTG	666	1.27
AAG/AGA/CTT/GAA/TCT/TTC	910	1.74
ACC/CAC/CCA/GGT/GTG/TGG	613	1.17
ACG/CGA/CGT/GAC/GTC/TCG	479	0.92
AGT/ACT/CTA/GTA/TAC/TAG	228	0.44
AGC/CAG/CTG/GCA/GCT/TGC	540	1.03
AGG/CCT/CTC/GAG/GGA/TCC	538	1.03
ATG/ATC/CAT/GAT/TCA/TGA	736	1.41
GGC/CCG/CGC/CGG/GCC/GCG	698	1.33
Tetra-	274	0.52
Penta-	48	0.09
Hexa-	125	0.24
Total	52288	100.00

## Discussion

These analyses extend those in Chen et al. [34] in three ways: firstly, by using larger sample such that these analyses cover almost all taxonomic virus genera; secondly, by making the data more comprehensive because the genome size varies greatly, ranging from 1682 bp (S170-(−)ssRNA-31, *Hepatitis delta virus*, NC_001653) to 407339 bp (S42-dsDNA-42, *Emiliania huxleyi virus 86*, NC_007346), ( Additional file 1); and thirdly, by applying statistically significant methods. The above extension made it possible to investigate the relationship between repetitiveness of microsatellites and genome size more fully and deeply.

The previous analysis [34] simply considered the correlation between microsatellites and genome size based on relatively small sample with 54 complete Hepatitis C virus (HCV) genomes, and they found that the number of SSRs is weakly correlated with genome size. We believe that Chen's result is lacking of statistical significance due to the relatively small sample size and uniform genome length. Here, the sample made up of 257 representative virus genome sequences was designed to investigate the relationship between SSRs and genome size on the level of the whole virus. The result of our data showed a very strong and significant positive relationship between the occurrence, or length of SSRs and genome size with the value of R^2^ = 0.919, P < 0.001 (Figure 2A) and R^2^ = 0.915, P < 0.001 (Figure 3A), respectively. That is, the longer the virus genome sequence, the more SSRs extracted. Hancock [15,35,36] confirmed that the simple sequence repeats were positively and significantly correlated with the genome size in both archaea and eubacteria, and SSRs accumulate preferentially in organisms with larger genomes. Moreover, there is evidence proved that short SSRs (1–4 bp length) exist in reduced genomes, but long SSRs (5–11 bp length) consist in larger genomes in prokaryotes [23]. The overall level of repetition in genomes is related to genome size and to the degree of repetition, and the entire genome accepts simple sequences in a concerted manner when its size increases [36,37]. A relative scarcity of repeating DNA is a major factor in causing the relatively compact size of the avian genome [38,39]. What's more, differences in genome size account for approximately 10% of the variance in genomic repetition in archaea and eubacteria [15], suggesting that other factors can also play important roles. DNA structure and base-stacking determined the number and length distributions of microsatellites in vertebrate genomes over evolutionary time [18]. Hosts are responsible for the variances of SSRs content to a certain degree. For example, with the similar genome size, viruses infecting vertebrates and invertebrates tend to be higher than viruses attacking bacteria in SSRs content, relative abundance and relative density of SSRs overall ( Additional file 15). This can be explained by the following statements. Genomes of reptiles are estimated to consist of about 30-50% repeats, birds have been estimated to consist of 15-20% of repeats [40,41], *Mus musculus* of 26.1% [42,43], and 44.9% of human genome were occupied by repeats [44,45]. While SSR tracts make up 2.4% of the *E. coli* genome [46], significantly less than vertebrates'. SSRs have been reported to be hot spots for recombination as well as sites for random integration [25,26]. Thus, the increase of viral SSRs content is maybe due to combining partial genome sequences of hosts in the process of infecting vertebrates and invertebrates. As we know, hosts evolved a number of defense systems in response to the challenge from parasites. Meanwhile, the parasites evolved multiple counter-defense mechanism as well under the selection pressure from hosts. Bacteria have developed CRISPR/Cas (CRISPR, Clustered regularly interspaced short palindromic repeats; Cas, CRISPR-associated) immune system to defend against bacteriophages by cleaving their DNA [47]. Antagonistic coevolution between bacteria and their ubiquitous parasites, bacteriophage (phage), is well known [48,49]. The genomic regions of CRISPR/Cas are hot spot of recombination, and CRISPR/Cas modules underwent rapid evolution in natural environments because of recurrent selection pressure exerted by coevolving viruses [50]. Meanwhile, viruses may combine partial CRISPR/Cas sequence in response to the counter-defense of bacteria. Therefore, it is no coincidence that SSRs content is high in both viruses that infect vertebrates and invertebrates and these hosts themselves. The recombination enhanced the virus's ability of infection and anti-immunity to a certain extent. Evolutionarily speaking, it is the result of selection in the process of interaction between viruses and hosts. It has proposed that reduced genome size represents an adaptation to the high rate of oxidative metabolism in birds, which results primarily from the demands of flight, and the relatively small genome size of birds in general may reflect the selective pressure to minimize the amount of repetitive DNA [51,52].

Overall, the longer genome sequence, the stronger capability the genome holding long SSRs. Each type of repeat unit is distributed in a certain length range of genomes. Mono- and di- SSRs were observed in almost all analyzed virus genomes; tri- repeats appeared to widely distribute in all virus genomes but it's number is obviously less than mono- and di- SSRs; tetra- SSRs as a common component consist in genomes with size more than 100 kb (94.4% of the genomes contain tetra- SSRs in group of genome > 100 kb). In contrast, it is relatively rare in genomes with the size < 100 kb; genomes containing penta- and hexa- SSRs are not more than 50% in < 100 kb group. Moreover, the number of tetra-, penta- and hexa- SSRs is very small (Table 1). Dinucleotide and trinucleotide SSRs were observed in all analyzed HIV genomes (genome size approximately 9 kb), but almost no tetra-, penta- and hexanucleotide SSRs were found [53]. Tetranucleotide SSRs are contained in 26.7% of the analyzed *Potyvirus* genomes (genome size approximately 10 kb), but the number of tetranucleotide SSRs is small [54]. The data of tetra-, penta- and hexanucleotide SSRs are also rare in Mycoplasma, but they are relatively sufficient in bacterial [46,55], fungal [56], plant [57], vertebrates [39,41] and human [58,59]. Those results confirmed that SSRs distribution is closely related to the genome size, indeed. The accumulation of simple sequence repeats would be attributed to the results of selection in the process of evolution. It has been well known that viruses such as influenza virus, hepatitis virus and human immunodeficiency virus (HIV) have a higher mutation rate to resist drugs, vaccines and so on during the process of replication and (or) recombination, which is one of the reasons for curing flu, hepatitis and acquired immunodeficiency syndrome (AIDS) with difficulty. Moreover, viruses lack complete repair mechanisms. Therefore, long SSRs can be poorly found in viruses. In the opinion of Mrázek et al. [23], small genomes have a strong negative selection against long SSRs due to their strong constraints against expansion.

## Conclusions

Genome size is an important factor in affecting the occurrence and the total length of SSRs, moreover, there is a positive correlation between them. Additionally, hosts are also responsible for the variances of SSRs content to a certain degree. For example, with similar genome sizes, viruses infecting vertebrates and invertebrates tend to be higher than viruses attacking bacteria in SSRs content, relative abundance and relative density of SSRs, overall. We inferred that maybe viruses combined partial genome sequences of hosts in infecting, resulting in relative large genome and high content of SSRs. Evolutionarily speaking, it is the result of selection in the process of interaction between viruses and hosts. Virus is a group of parasite, so studying of SSRs in viruses is helpful to the research of many etiopathogenesis of its hosts.

## Misc

Xiangyan Zhao, Yonglei Tian, both authors contributed equally to this work.

## Competing interest

The authors declare that they have no competing interests.

## Authors' contributions

ZT and ML conceived and designed this study. XZ and YT performed and drafted manuscript. HF, QO, YT and YN participated in the data processing. RY, JJ, GS and RY involved in revising the manuscript critically for important intellectual content. All authors read and approved the final manuscript.

## Supplementary Material

Additional file 1 List of the basic information of all analyzed viruses. Click here for file

Additional file 2 Occurrence of SSRs in analyzed virus genomes. Click here for file

Additional file 3 Length (bp) of SSRs in analyzed virus genomes. Click here for file

Additional file 4 Relative abundance of SSRs in analyzed virus genomes. Click here for file

Additional file 5 Relative density of SSRs in analyzed virus genomes. Click here for file

Additional file 6 **Scatter plots of SSRs relative abundance versus genome size.** (*A*)Scatter plot of SSRs relative abundances in all analyzed virus genomes. (*B*) Scatter plot of SSRs relative abundances in analyzed virus genomes with size of < 30000 bp. (*C*) Scatter plot of SSRs relative abundances in analyzed virus genomes with size of > 30000 bp. Click here for file

Additional file 7 **Scatter plots of SSRs relative density versus genome size.** (*A*) Scatter plot of SSRs relative densities in all analyzed virus genomes. (*B*) Scatter plot of SSRs relative densities in analyzed virus genomes with size of < 30000 bp. (*C*) Scatter plot of SSRs relative densities in analyzed virus genomes with size of > 30000 bp. Click here for file

Additional file 8 Descriptive statistics of SSRs variables. Click here for file

Additional file 9 Matrix of correlation coefficients and 1-tailed tests between SSRs. Click here for file

Additional file 10 **Scree plot.** It displays the "cliff" and the "screes" vividly, which can be visually proved that the applicability of PCA is very good to the current data set. Click here for file

Additional file 11 Occurrence of mono- SSRs in analyzed virus genomes. Click here for file

Additional file 12 Occurrence of di- SSRs in analyzed virus genomes. Click here for file

Additional file 13 Occurrence of tri- SSRs in analyzed virus genomes. Click here for file

Additional file 14 Occurrence of tetra-, penta- and hexa- SSRs in analyzed virus genomes. Click here for file

Additional file 15 Hosts of analyzed virus genomes. Click here for file
