# Supplementary material for: Coevolution between simple sequence repeats (SSRs) and virus genome size
Source: BMC Genomics. 2012 Aug 30;13:435. doi: 10.1186/1471-2164-13-435 (PMC3585866; doi:10.1186/1471-2164-13-435)
Supplement: Additional file 1 — List of the basic information of all analyzed viruses. [file 1471-2164-13-435-S1.pdf]

Additional file 1 List of the basic information of all analyzed viruses

Family	Subfamily	Genus name	Type species name	Acc. no.	Size (bp)	Coding S (%)	Genome no.
Myoviridae	—	T4-like viruses	Enterobacteria phage T4	NC_000866	168903	93	S1-dsDNA-1
		P1-like viruses	Enterobacteria phage P1	NC_005856	94800	86	S2-dsDNA-2
		P2-like viruses	Enterobacteria phage P2	NC_001895	33593	92	S3-dsDNA-3
		Mulike viruses	Enterobacteria phage Mu	NC_000929	36717	94	S4-dsDNA-4
		SP01like viruses	Bacillus phage SPO1	NC_011421	132562	89	S5-dsDNA-5
Siphoviridae	—	λ-like phages	Enterobacteria phage λ	NC_001416	48502	87	S6-dsDNA-6
		T1-like viruses	Enterobacteria phage T1	NC_005833	48836	91	S7-dsDNA-7
		T5-like viruses	Enterobacteria phage T5	NC_005859	121750	79	S8-dsDNA-8
		c2-like viruses	Lactococcus phage C2	NC_001706	22172	92	S9-dsDNA-9
		L5-like viruses	Mycobacterium phage L5	NC_001335	52297	87	S10-dsDNA-10
		N15-like viruses	Enterobacteria phage N15	NC_001901	46375	90	S11-dsDNA-11
		φ 31-like viruses	Streptomyces phage ϕC31	NC_001978	41491	89	S12-dsDNA-12
Podoviridae	—	T7-like viruses	Enterobacteria phage T7	NC_001604	39937	92	S13-dsDNA-13
		φ 29-like viruses	Bacillus phage φ 29	NC_011048	19282	93	S14-dsDNA-14
		P22-like viruses	Enterobacteria phage P22	NC_002371	41724	90	S15-dsDNA-15
		N4-like viruses	Enterobacteria phage N4	NC_008720	70153	94	S16-dsDNA-16
Tectiviridae	—	Tectivirus	Enterobacteria phage PRD1	NC_001421	14927	95	S17-dsDNA-17
Corticoviridae	—	Corticovirus	Pseudoalteromonas phage PM2	NC_000867	10079	92	S18-dsDNA-18
Plasmaviridae	—	Plasmavirus	Acholeplasma phage L2	NC_001447	11965	82	S19-dsDNA-19
Lipothrixviridae	—	Betalipothrixvirus	Sulfolobus islandicus filamentous virus	NC_003214	40900	88	S20-dsDNA-20
		Gammalipothrixvirus	Acidianus filamentous virus 1	NC_005830	20869	91	S21-dsDNA-21
Rudiviridae	—	Rudivirus	Sulfolobus islandicus rod-shaped virus 2	NC_004086	35450	79	S22-dsDNA-22
Fuselloviridae	—	Fusellovirus	Sulfolobus spindle-shaped virus 1	NC_001338	15465	90	S23-dsDNA-23
—	—	Salterprovirus	His1 virus	NC_007914	14462	91	S24-dsDNA-24
Poxviridae	Chordopoxvirinae	Orthopoxvirus	Vaccinia virus	NC_006998	194711	88	S25-dsDNA-25
		Parapoxvirus	Orf virus	NC_005336	139962	89	S26-dsDNA-26
		Avipoxvirus	Fowlpox virus	NC_002188	288539	84	S27-dsDNA-27
		Capripoxvirus	Sheeppox virus	NC_004002	149955	93	S28-dsDNA-28
		Leporipoxvirus	Myxoma virus	NC_001132	161773	95	S29-dsDNA-29
		Suipoxvirus	Swinepox virus	NC_003389	146454	96	S30-dsDNA-30
		Molluscipoxvirus	Molluscum contagiosum virus	NC_001731	190289	85	S31-dsDNA-31
		Yatapoxvirus	Yaba monkey tumor virus	NC_005179	134721	94	S32-dsDNA-32
	Entomopoxvirinae	Betaentomopoxvirus	Amsacta moorei entomopoxvirus 'L'	NC_002520	232392	91	S33-dsDNA-33
Asfarviridae	—	Asfivirus	African swine fever virus	NC_001659	170101	87	S34-dsDNA-34
Iridoviridae	—	Iridovirus	invertebrates iridescent virus 6	NC_003038	212482	90	S35-dsDNA-35
		Chloriridovirus	invertebrates iridescent virus 3	NC_008187	191100	68	S36-dsDNA-36

		Ranavirus	Frog virus 3	NC_005946	105903	79	S37-dsDNA-37
		Lymphocystivirus	Lymphocystis disease virus 1	NC_001824	102653	92	S38-dsDNA-38
		Megalocytivirus	Infectious spleen and kidney necrosis virus	NC_003494	111362	93	S39-dsDNA-39
Phycodnaviridae	—	Chlorovirus	Paramecium bursaria Chlorella virus 1	NC_000852	330743	90	S40-dsDNA-40
		Phaeovirus	Ectocarpus siliculosus virus 1	NC_002687	335593	70	S41-dsDNA-41
		Coccolithovirus	Emiliania huxleyi virus 86	NC_007346	407339	90	S42-dsDNA-42
Baculoviridae	—	Nucleopolyhedrovirus	Autographa californica nucleopolyhedrovirus	NC_001623	133894	91	S43-dsDNA-43
	—	Granulovirus	Cydia pomonella granulovirus	NC_002816	123500	88	S44-dsDNA-44
Nimaviridae	—	Whispovirus	Shrimp white spot syndrome virus	NC_003225	305107	92	S45-dsDNA-45
Herpesviridae	—	Ictalurivirus	Ictalurid herpesvirus 1	NC_001493	134226	83	S46-dsDNA-46
	Alphaherpesvirinae	Simplexvirus	Human herpesvirus 1	NC_001806	152261	79	S47-dsDNA-47
		Varicellovirus	Human herpesvirus 3	NC_001348	124884	89	S48-dsDNA-48
		Mardivirus	Gallid herpesvirus 2	NC_002229	177874	73	S49-dsDNA-49
		Iltovirus	Gallid herpesvirus 1	NC_006623	148687	81	S50-dsDNA-50
	Betaherpesvirinae	Cytomegalovirus	Human herpesvirus 5	NC_006273	235646	79	S51-dsDNA-51
		Muromegalovirus	Murid herpesvirus 1	NC_004065	230278	72	S52-dsDNA-52
		Roseolovirus	Human herpesvirus 6	NC_001664	159322	79	S53-dsDNA-53
	Gammaherpesvirinae	Lymphocryptovirus	Human herpesvirus 4	NC_009334	172764	68	S54-dsDNA-54
		Rhadinovirus	Saimiriine herpesvirus 2	NC_001350	112930	87	S55-dsDNA-55
Adenoviridae	—	Mastadenovirus	Human adenovirus C	NC_001405	35937	90	S56-dsDNA-56
		Aviadenovirus	Fowl adenovirus A	AC_000014	43804	84	S57-dsDNA-57
		Atadenovirus	Ovine adenovirus D	NC_004037	29576	92	S58-dsDNA-58
		Siadenovirus	Frog adenovirus 1	NC_002501	26163	92	S59-dsDNA-59
Polyomaviridae	—	Polyomavirus	Simian virus 40	NC_001669	5243	89	S60-dsDNA-60
Papillomaviridae	—	Alphapapillomavirus	Human papillomavirus type 32	NC_001586	7961	84	S61-dsDNA-61
		Betapapillomavirus	Human papillomavirus type 5	NC_001531	7746	92	S62-dsDNA-62
		Gammapapillomavirus	Human papillomavirus type 4	NC_001457	7353	92	S63-dsDNA-63
		Deltapapillomavirus	European elk papillomavirus	NC_001524	8095	91	S64-dsDNA-64
		Epsilonpapillomavirus	Bovine papillomavirus type 5	NC_004195	7841	86	S65-dsDNA-65
		Zetapapillomavirus	Equus caballus papillomavirus 1	NC_003748	7610	87	S66-dsDNA-66
		Etapapillomavirus	Fringilla coelebs papillomavirus	NC_004068	7729	89	S67-dsDNA-67
		Thetapapillomavirus	Psittacus erithacus timneh papillomavirus	NC_003973	7304	93	S68-dsDNA-68
		Iotapapillomavirus	Mastomys natalensis papillomavirus	NC_001605	7687	92	S69-dsDNA-69
		Kappapapillomavirus	Cottontail rabbit papillomavirus	NC_001541	7868	90	S70-dsDNA-70
		Lambdapapillomavirus	Canine oral papillomavirus	NC_001619	8607	77	S71-dsDNA-71
		Mupapillomavirus	Human papillomavirus 1	NC_001356	7815	86	S72-dsDNA-72
		Nupapillomavirus	Human papillomavirus type 41	NC_001354	7614	90	S73-dsDNA-73
		Xipapillomavirus	Bovine papillomavirus 3	NC_004197	7276	91	S74-dsDNA-74

		Omicronpapillomavirus	Phocoena spinipinnis papillomavirus	NC_003348	7879	89	S75-dsDNA-75
Polydnaviridae	—	Ichnovirus	Campoletis sonorensis ichnovirus superhelical segment A	NC_007997	6138	N/A	S76-dsDNA-76
			Campoletis sonorensis ichnovirus superhelical segment A2	NC_008005	6283	N/A	
			Campoletis sonorensis ichnovirus superhelical segment B	NC_007987	6567	N/A	
			Campoletis sonorensis ichnovirus superhelical segment C	NC_007986	7276	N/A	
			Campoletis sonorensis ichnovirus superhelical segment D	NC_008008	7604	N/A	
			Campoletis sonorensis ichnovirus superhelical segment E	NC_007988	7760	N/A	
			Campoletis sonorensis ichnovirus superhelical segment F	NC_008004	7887	N/A	
			Campoletis sonorensis ichnovirus superhelical segment G	NC_007989	8284	N/A	
			Campoletis sonorensis ichnovirus superhelical segment G2	NC_007990	8171	N/A	
			Campoletis sonorensis ichnovirus superhelical segment H	NC_007991	8315	N/A	
			Campoletis sonorensis ichnovirus superhelical segment I	NC_007992	8600	N/A	
			Campoletis sonorensis ichnovirus superhelical segment I2	NC_007995	8864	N/A	
			Campoletis sonorensis ichnovirus superhelical segment J	NC_007993	9155	N/A	
			Campoletis sonorensis ichnovirus superhelical segment L	NC_007994	9391	N/A	
			Campoletis sonorensis ichnovirus superhelical segment M	NC_007996	10223	N/A	
			Campoletis sonorensis ichnovirus superhelical segment N	NC_008006	10757	N/A	
			Campoletis sonorensis ichnovirus superhelical segment O1	NC_007998	11285	N/A	
			Campoletis sonorensis ichnovirus superhelical segment P	NC_007999	11841	N/A	
			Campoletis sonorensis ichnovirus superhelical segment Q	NC_007985	12098	18	
			Campoletis sonorensis ichnovirus superhelical segment T	NC_008000	14531	N/A	
			Campoletis sonorensis ichnovirus superhelical segment U	NC_008001	14825	N/A	

			Campoletis sonorensis ichnovirus superhelical segment V	NC_008003	15510	6	
			Campoletis sonorensis ichnovirus chromosome segment W	NC_008007	15812	7	
			Campoletis sonorensis ichnovirus chromosome segment Z	NC_008002	19557	N/A	
Ascoviridae	—	Ascovirus	Spodoptera frugiperda ascovirus 1a	NC_008361	156922	68	S77-dsDNA-77
Inoviridae	—	Inovirus	Enterobacteria phage M13	NC_003287	6407	91	S78-ssDNA-1
		Plectrovirus	Acholeplasma phage MV-L1	NC_001341	4491	48	S79-ssDNA-2
Microviridae	—	Microvirus	Enterobacteria phage φX174	NC_001422	5386	95	S80-ssDNA-3
		Spiromicrovirus	Spiroplasma phage 4	NC_003438	4421	93	S81-ssDNA-4
		Bdellomicrovirus	Bdellovibrio phage phiMH2K	NC_002643	4594	86	S82-ssDNA-5
		Chlamydiamicrovirus	Chlamydia phage Chp1	NC_001741	4877	94	S83-ssDNA-6
Geminiviridae	—	Mastrevirus	Maize streak virus - A[South Africa]	NC_001346	2690	82	S84-ssDNA-7
		Curtovirus	Beet curly top virus - California	NC_001412	2994	88	S85-ssDNA-8
		Begomovirus	Bean golden yellow mosaic virus DNA A	NC_001439	2647	87	S86-ssDNA-9
			Bean golden yellow mosaic virus DNA B	NC_001438	2585	63	
		Topocuvirus	Tomato pseudo-curly top virus	NC_003825	2861	89	S87-ssDNA-10
Circoviridae	—	Circovirus	Porcine circovirus 1	NC_001792	1758	92	S88-ssDNA-11
		Gyrovirus	Chicken anemia virus	NC_001427	2319	90	S89-ssDNA-12
—	—	Anellovirus	Torque teno virus (TTV)	NC_002076	3852	70	S90-ssDNA-13
Nanoviridae	—	Nanovirus	Subterranean clover stunt virus DNA 1	NC_003813	1001	33	S91-ssDNA-14
			Subterranean clover stunt virus DNA 2	NC_003814	1022	82	
			Subterranean clover stunt virus DNA 3	NC_003815	991	49	
			Subterranean clover stunt virus DNA 4	NC_003816	1002	46	
			Subterranean clover stunt virus DNA 5	NC_003817	998	51	
			Subterranean clover stunt virus DNA 6	NC_003818	1017	84	
			Subterranean clover stunt virus DNA 7	NC_003819	988	44	
			Subterranean clover stunt virus DNA 8	NC_003812	1005	85	
		Babuvirus	Banana bunchy top virus DNA 1	NC_003479	1111	77	S92-ssDNA-15
			Banana bunchy top virus DNA 2	NC_003475	1060	N/A	

			Banana bunchy top virus DNA 3	NC_003473	1075	49	
			Banana bunchy top virus DNA 4	NC_003474	1043	33	
			Banana bunchy top virus DNA 5	NC_003477	1018	47	
			Banana bunchy top virus DNA 6	NC_003476	1089	42	
Parvoviridae	Parvovirinae	Parvovirus	Minute virus of mice	NC_001510	5149	86	S93-ssDNA-16
		Erythrovirus	Human parvovirus B19	NC_000883	5594	77	S94-ssDNA-17
		Dependovirus	Adeno-associated virus - 2	NC_001401	4679	87	S95-ssDNA-18
		Amdovirus	Aleutian mink disease virus	NC_001662	4801	82	S96-ssDNA-19
		Bocavirus	Bovine parvovirus	NC_001540	5517	82	S97-ssDNA-20
	Densovirinae	Densovirus	Junonia coenia densovirus	NC_004284	5908	78	S98-ssDNA-21
		Iteravirus	Bombyx mori densovirus 5	NC_004287	5078	84	S99-ssDNA-22
		Brevidensovirus	Aedes aegypti densovirus	NC_012636	3776	92	S100-ssDNA-23
		Pefudensovirus	Periplaneta fuliginosa densovirus	NC_000936	5454	94	S101-ssDNA-24
Hepadnaviridae	—	Orthohepadnavirus	Hepatitis B virus	NC_003977	3215	100	S102-dsDNA-RT-1
		Avihepadnavirus	Duck hepatitis B virus	NC_001344	3027	100	S103-dsDNA-RT-2
Caulimoviridae	—	Caulimovirus	Cauliflower mosaic virus	NC_001497	8024	89	S104-dsDNA-RT-3
		Soymovirus	Soybean chlorotic mottle virus	NC_001739	8178	93	S105-dsDNA-RT-4
		Cavemovirus	Cassava vein mosaic virus	NC_001648	8159	93	S106-dsDNA-RT-5
		Tungrovirus	Rice tungro bacilliform virus	NC_001914	8002	89	S107-dsDNA-RT-6
		Badnavirus	Commelina yellow mottle virus	NC_001343	7489	89	S108-dsDNA-RT-7
		Petuvirus	Petunia vein clearing virus	NC_001839	7206	90	S109-dsDNA-RT-8
Retroviridae	Orthoretrovirinae	Betaretrovirus	Mouse mammary tumor virus	NC_001503	8805	91	S110-ssRNA-RT-1
		Gammaretrovirus	Rauscher murine leukemia virus	NC_001819	8282	86	S111-ssRNA-RT-2
		Alpharetrovirus	Avian leukosis virus - RSA	NC_001408	7286	65	S112-ssRNA-RT-3
		Deltaretrovirus	Bovine leukemia virus	NC_001414	8419	79	S113-ssRNA-RT-4
		Lentivirus	Human immunodeficiency virus 1	NC_001802	9181	93	S114-ssRNA-RT-5
		Epsilonretrovirus	Walleye dermal sarcoma virus	NC_001867	12708	86	S115-ssRNA-RT-6
	Spumaretrovirinae	Spumavirus	Simian foamy virus	NC_001364	13246	78	S116-ssRNA-RT-7
Cystoviridae	—	Cystovirus	Pseudomonas phage phi-6 segment L	NC_003715	6374	90	S117-dsRNA-1
			Pseudomonas phage phi-6 segment M	NC_003716	4063	68	
			Pseudomonas phage phi-6 segment S	NC_003714	2948	66	

Reoviridae	—	Orthoreovirus	Mammalian orthoreovirus 3 segment L1	NC_004282	3860	98	S118-dsRNA-2
			Mammalian orthoreovirus 3 segment L2	NC_004275	3916	98	
			Mammalian orthoreovirus 3 segment L3	NC_004274	3901	98	
			Mammalian orthoreovirus 3 segment M1	NC_004280	2304	95	
			Mammalian orthoreovirus 3 segment M2	NC_004278	2207	96	
			Mammalian orthoreovirus 3 segment M3	NC_004281	2235	96	
			Mammalian orthoreovirus 3 segment S1	NC_004277	1416	96	
			Mammalian orthoreovirus 3 segment S2	NC_004279	1331	94	
			Mammalian orthoreovirus 3 segment S3	NC_004283	1198	91	
			Mammalian orthoreovirus 3 segment S4	NC_004276	1196	91	
		Orbivirus	Bluetongue virus segment 1	NC_006023	3944	99	S119-dsRNA-3
			Bluetongue virus segment 2	NC_006013	2953	97	
			Bluetongue virus segment 3	NC_006014	2772	97	
			Bluetongue virus segment 4	NC_006024	1980	97	
			Bluetongue virus segment 5	NC_006025	1769	93	
			Bluetongue virus segment 6	NC_006010	1638	96	
			Bluetongue virus segment 7	NC_006022	1156	90	
			Bluetongue virus segment 8	NC_006007	1125	94	
			Bluetongue virus segment 9	NC_006008	1049	94	
			Bluetongue virus segment 10	NC_006015	822	83	
		Rotavirus	Rotavirus A segment 1	NC_011507	3267	100	S120-dsRNA-4
			Rotavirus A segment 2	NC_011506	2649	100	
			Rotavirus A segment 3	NC_011508	2508	100	
			Rotavirus A segment 4	NC_011510	2331	100	
			Rotavirus A segment 5	NC_011500	1491	100	
			Rotavirus A segment 6	NC_011509	1194	100	
			Rotavirus A segment 7	NC_011502	954	100	
			Rotavirus A segment 8	NC_011501	948	100	
			Rotavirus A segment 9	NC_011503	981	100	
			Rotavirus A segment 10	NC_011504	528	100	
			Rotavirus A segment 11	NC_011505	597	100	
		Coltivirus	Colorado tick fever virus segment 1	NC_004181	4350	99	S121-dsRNA-5
			Colorado tick fever virus segment 2	NC_004182	3909	92	

			Colorado tick fever virus segment 3	NC_004183	3586	98	
			Colorado tick fever virus segment 4	NC_004184	3157	97	
			Colorado tick fever virus segment 5	NC_004185	2432	92	
			Colorado tick fever virus segment 6	NC_004186	2141	97	
			Colorado tick fever virus segment 7	NC_004187	2133	96	
			Colorado tick fever virus segment 8	NC_004188	2029	97	
			Colorado tick fever virus segment 9	NC_004180	1884	96	
			Colorado tick fever virus segment 10	NC_004189	1880	96	
			Colorado tick fever virus segment 11	NC_004191	998	75	
			Colorado tick fever virus segment 12	NC_004190	675	82	
		Aquareovirus	Aquareovirus A segment 1	NC_007582	3947	98	S122-dsRNA-6
			Aquareovirus A segment 2	NC_007583	3867	96	
			Aquareovirus A segment 3	NC_007584	3690	98	
			Aquareovirus A segment 4	NC_007585	1619	72	
			Aquareovirus A segment 5	NC_007586	2242	N/A	
			Aquareovirus A segment 6	NC_007592	2052	94	
			Aquareovirus A segment 7	NC_007587	1395	N/A	
			Aquareovirus A segment 8	NC_007588	1317	95	
			Aquareovirus A segment 9	NC_007589	1118	94	
			Aquareovirus A segment 10	NC_007590	985	91	
			Aquareovirus A segment 11	NC_007591	783	90	
		Cypovirus	Lymantria dispar cypovirus 1 segment 1	NC_003016	4164	96	S123-dsRNA-7
			Lymantria dispar cypovirus 1 segment 2	NC_003017	3853	95	
			Lymantria dispar cypovirus 1 segment 3	NC_003018	3846	96	
			Lymantria dispar cypovirus 1 segment 4	NC_003019	3262	97	
			Lymantria dispar cypovirus 1 segment 5	NC_003020	2851	92	
			Lymantria dispar cypovirus 1 segment 6	NC_003021	1792	94	
			Lymantria dispar cypovirus 1 segment 7	NC_003022	1501	89	
			Lymantria dispar cypovirus 1 segment 8	NC_003023	1332	88	
			Lymantria dispar cypovirus 1 segment 9	NC_003024	1187	81	
			Lymantria dispar cypovirus 1 segment 10	NC_003025	944	79	
		Fijivirus	Fiji disease virus segment 1	NC_007159	4532	97	S124-dsRNA-8
			Fiji disease virus segment 2	NC_007154	3820	93	

			Fiji disease virus segment 3	NC_007158	3623	96	
			Fiji disease virus chromosome segment 4	NC_007155	3568	96	
			Fiji disease virus segment 5	NC_007160	3150	95	
			Fiji disease virus segment 6	NC_007157	2831	89	
			Fiji disease virus segment 7	NC_007163	2194	92	
			Fiji disease virus segment 8	NC_007161	1959	91	
			Fiji disease virus segment 9	NC_007156	1843	88	
			Fiji disease virus segment 10	NC_007162	1819	91	
		Phytoreovirus	Rice gall dwarf virus segment S1	NC_009248	4505	97	S125-dsRNA-9
			Rice gall dwarf virus segment S2	NC_009244	3514	98	
			Rice gall dwarf virus segment S3	NC_009243	3224	95	
			Rice gall dwarf virus segment S4	NC_009249	2622	83	
			Rice gall dwarf virus segment S5	NC_009247	2542	94	
			Rice gall dwarf virus segment S6	NC_009250	1648	89	
			Rice gall dwarf virus segment S7	NC_009251	1652	92	
			Rice gall dwarf virus segment S8	NC_009241	1578	81	
			Rice gall dwarf virus segment S9	NC_009246	1202	80	
			Rice gall dwarf virus segment S10	NC_009242	1198	80	
			Rice gall dwarf virus segment S11	NC_009245	1171	91	
			Rice gall dwarf virus segment S12	NC_009252	853	72	
		Oryzavirus	Rice ragged stunt virus segment 1	NC_003749	3849	96	S126-dsRNA-10
			Rice ragged stunt virus segment 2	NC_003750	3808	93	
			Rice ragged stunt virus segment 3	NC_003751	3699	95	
			Rice ragged stunt virus segment 4	NC_003771	3823	98	
			Rice ragged stunt virus segment 5	NC_003759	2682	90	
			Rice ragged stunt virus segment 6	NC_003752	2157	82	
			Rice ragged stunt virus segment 7	NC_003770	1938	94	
			Rice ragged stunt virus segment 8	NC_003758	1914	93	
			Rice ragged stunt virus segment 9	NC_003757	1132	89	
			Rice ragged stunt virus segment 10	NC_003769	1162	76	
		Seadornavirus	Banna virus segment 1	NC_004211	3747	97	S127-dsRNA-11
			Banna virus segment 2	NC_004217	3048	93	
			Banna virus segment 3	NC_004218	2400	90	
			Banna virus segment 4	NC_004219	2038	84	
			Banna virus segment 5	NC_004220	1716	88	
			Banna virus segment 6	NC_004221	1671	76	
			Banna virus segment 7	NC_004204	1136	81	
			Banna virus segment 8	NC_004203	1119	81	
			Banna virus segment 9	NC_004202	1101	77	
			Banna virus segment 10	NC_004201	977	76	
			Banna virus segment 11	NC_004200	867	62	
			Banna virus segment 12	NC_004198	862	72	

		Mycoreovirus	Mycoreovirus 1 segment 1	NC_010743	4127	98	S128-dsRNA-12
			Mycoreovirus 1 segment 2	NC_010744	3846	96	
			Mycoreovirus 1 segment 3	NC_010745	3258	98	
			Mycoreovirus 1 segment 4	NC_010746	2269	95	
			Mycoreovirus 1 segment 5	NC_010747	2056	94	
			Mycoreovirus 1 segment 6	NC_010748	2023	96	
			Mycoreovirus 1 segment 7	NC_010749	1539	91	
			Mycoreovirus 1 segment 8	NC_010750	1536	94	
			Mycoreovirus 1 segment 9	NC_010751	1072	83	
			Mycoreovirus 1 segment 10	NC_010752	975	76	
			Mycoreovirus 1 segment 11	NC_010753	732	41	
Birnaviridae	—	Aquabirnavirus	Infectious pancreatic necrosis virus segment A	NC_001915	3097	95	S129-dsRNA-13
			Infectious pancreatic necrosis virus segment B	NC_001916	2784	91	
	—	Avibirnavirus	Infectious bursal disease virus segment A	NC_004178	3183	96	S130-dsRNA-14
			Infectious bursal disease virus segment B	NC_004179	2715	97	
	—	Entomobirnavirus	Drosophila x virus segment A	NC_004177	3360	92	S131-dsRNA-15
			Drosophila x virus segment B	NC_004169	3243	92	
Totiviridae	—	Totivirus	Saccharomyces cerevisiae virus L-A (L1)	NC_003745	4579	98	S132-dsRNA-16
		Giardiavirus	Giardia lamblia virus	NC_003555	6277	89	S133-dsRNA-17
		Leishmaniavirus	Leishmania RNA virus 1 - 1	NC_002063	5284	94	S134-dsRNA-18
Partitiviridae	—	Partitivirus	Atkinsonella hypoxylon partitivirus RNA 1	NC_003470	2180	91	S135-dsRNA-19
			Atkinsonella hypoxylon partitivirus RNA 2	NC_003471	2135	91	
			Atkinsonella hypoxylon partitivirus RNA 3	NC_003472	1790	N/A	
		Alphacryptovirus	White clover cryptic virus 1 RNA1	NC_006275	1955	94	S136-dsRNA-20
			White clover cryptic virus 1 RNA2	NC_006276	1708	85	
Chrysoviridae	—	Chrysovirus	Penicillium chrysogenum virus segment 1	NC_007539	3562	94	S137-dsRNA-21
			Penicillium chrysogenum virus segment 2	NC_007540	3200	92	
			Penicillium chrysogenum virus segment 3	NC_007541	2976	92	
			Penicillium chrysogenum virus segment 4	NC_007542	2902	87	
Hypoviridae	—	Hypovirus	Cryphonectria hypovirus 1	NC_001492	12734	89	S138-dsRNA-22
—	—	Endornavirus	Vicia faba endornavirus	NC_007648	17635	99	S139-dsRNA-23

Bornaviridae	—	Bornavirus	Borna disease virus	NC_001607	8910	97	S140-(-)ssRNA-1
Rhabdoviridae	—	Vesiculovirus	Vesicular stomatitis Indiana virus	NC_001560	11161	95	S141-(-)ssRNA-2
		Lyssavirus	Rabies virus	NC_001542	11932	91	S142-(-)ssRNA-3
		Ephemerovirus	Bovine ephemeral fever virus	NC_002526	14900	95	S143-(-)ssRNA-4
		Cytorhabdovirus	Lettuce necrotic yellows virus	NC_007642	12807	90	S144-(-)ssRNA-5
		Nucleorhabdovirus	Taro vein chlorosis virus	NC_006942	12020	95	S145-(-)ssRNA-6
		Novirhabdovirus	Infectious hematopoietic necrosis virus	NC_001652	11131	92	S146-(-)ssRNA-7
Filoviridae	—	Marburgvirus	Lake Victoria marburgvirus - Musoke	NC_001608	19111	76	S147-(-)ssRNA-8
		Ebolavirus	Zaire ebolavirus	NC_002549	18959	76	S148-(-)ssRNA-9
Paramyxoviridae	Paramyxovirinae	Respirovirus	Sendai virus	NC_001552	15384	94	S149-(-)ssRNA-10
		Morbillivirus	Measles virus	NC_001498	15894	89	S150-(-)ssRNA-11
		Rubulavirus	Mumps virus	NC_002200	15384	92	S151-(-)ssRNA-12
		Henipavirus	Hendra virus	NC_001906	18234	82	S152-(-)ssRNA-13
		Avulavirus	Newcastle disease virus B1	NC_002617	15186	90	S153-(-)ssRNA-14
	Pneumovirinae	Pneumovirus	Human respiratory syncytial virus	NC_001781	15225	89	S154-(-)ssRNA-15
		Metapneumovirus	Avian metapneumovirus	NC_007652	14071	92	S155-(-)ssRNA-16
—	—	Varicosavirus	Lettuce big-vein associated virus segment 1	NC_011558	6797	90	S156-(-)ssRNA-17
			Lettuce big-vein associated virus segment 2	NC_011568	6081	76	
—	—	Ophiovirus	Citrus psorosis virus RNA1	NC_006314	8186	96	S157-(-)ssRNA-18
			Citrus psorosis virus RNA2	NC_006315	1645	86	
			Citrus psorosis virus RNA3	NC_006316	1447	91	
Orthomyxoviridae	—	Influenzavirus A	Influenza A virus (A/Korea/426/68(H2N2)) segment 1	NC_007378	2341	97	S158-(-)ssRNA-19
			Influenza A virus (A/Korea/426/68(H2N2)) segment 2	NC_007375	2341	97	
			Influenza A virus (A/Korea/426/68(H2N2)) segment 3	NC_007376	2233	96	
			Influenza A virus (A/Korea/426/68(H2N2)) segment 4	NC_007374	1773	95	
			Influenza A virus (A/Korea/426/68(H2N2)) segment 5	NC_007381	1497	100	
			Influenza A virus (A/Korea/426/68(H2N2)) segment 6	NC_007382	1410	100	
			Influenza A virus (A/Korea/426/68(H2N2)) segment 7	NC_007377	1027	95	
			Influenza A virus (A/Korea/426/68(H2N2)) segment 8	NC_007380	838	100	
	—	Influenzavirus C	Influenza C virus (C/Ann Arbor/1/50) segment 1	NC_006307	2325	100	S159-(-)ssRNA-20

			Influenza C virus (C/Ann Arbor/1/50) segment 2	NC_006308	2265	100	
			Influenza C virus (C/Ann Arbor/1/50) segment 3	NC_006309	2130	100	
			Influenza C virus (C/Ann Arbor/1/50) segment 4	NC_006310	1968	100	
			Influenza C virus (C/Ann Arbor/1/50) segment 5	NC_006311	1807	93	
			Influenza C virus (C/Ann Arbor/1/50) segment 6	NC_006312	1125	100	
			Influenza C virus (C/Ann Arbor/1/50) segment 7	NC_006306	935	92	
	—	Thogotovirus	Thogoto virus segment 1	NC_006508	2375	97	S160-(-)ssRNA-21
			Thogoto virus segment 2	NC_006495	2212	96	
			Thogoto virus segment 3	NC_006496	1927	96	
			Thogoto virus segment 4	NC_006506	1574	97	
			Thogoto virus segment 5	NC_006507	1418	96	
			Thogoto virus segment 6	NC_006504	955	95	
	—	Influenzavirus B	Influenza B virus RNA 1	NC_002204	2368	95	S161-(-)ssRNA-22
			Influenza B virus RNA 2	NC_002205	2313	100	
			Influenza B virus RNA 3	NC_002206	2204	98	
			Influenza B virus RNA 4	NC_002207	1882	93	
			Influenza B virus RNA 5	NC_002208	1841	91	
			Influenza B virus RNA 6	NC_002209	1557	90	
			Influenza B virus RNA 7	NC_002210	1191	90	
			Influenza B virus RNA 8	NC_002211	1096	93	
	—	Isavirus	Infectious salmon anemia virus segment 1	NC_006505	2169	100	S162-(-)ssRNA-23
			Infectious salmon anemia virus segment 2	NC_006503	2185	97	
			Infectious salmon anemia virus segment 3	NC_006502	2046	90	
			Infectious salmon anemia virus segment 4	NC_006501	1787	97	
			Infectious salmon anemia virus segment 5	NC_006500	1504	88	
			Infectious salmon anemia virus segment 6	NC_006499	1323	89	
			Infectious salmon anemia virus segment 7	NC_006498	966	90	
			Infectious salmon anemia virus segment 8	NC_006497	736	95	
Bunyaviridae	—	Orthobunyavirus	Bunyamwera virus L segment	NC_001925	6875	97	S163-(-)ssRNA24

			Bunyamwera virus M segment	NC_001926	4458	96	
			Bunyamwera virus S segment	NC_001927	961	73	
	—	Hantavirus	Hantaan virus M	NC_005219	3616	94	S164-(-)ssRNA-25
			Hantaan virus L	NC_005222	6533	98	
			Hantaan virus S	NC_005218	1696	76	
	—	Nairovirus	Dugbe virus segment L	NC_004159	12255	98	S165-(-)ssRNA-26
			Dugbe virus segment M	NC_004158	4888	95	
			Dugbe virus segment S	NC_004157	1716	84	
	—	Phlebovirus	Rift Valley fever virus M	NC_014396	3885	92	S166-(-)ssRNA-27
			Rift Valley fever virus S	NC_014395	1690	90	
			Rift Valley fever virus L	NC_014397	6404	98	
	—	Tospovirus	Tomato spotted wilt virus RNA L	NC_002052	8897	96	S167-(-)ssRNA-28
			Tomato spotted wilt virus RNA M	NC_002050	4821	74	
			Tomato spotted wilt virus RNA S	NC_002051	2916	86	
—	—	Tenuivirus	Rice stripe virus RNA 1	NC_003755	8970	97	S168-(-)ssRNA-29
			Rice stripe virus RNA 2	NC_003754	3514	88	
			Rice stripe virus RNA 3	NC_003776	2504	64	
			Rice stripe virus RNA 4	NC_003753	2157	64	
Arenaviridae	—	Arenavirus	Lymphocytic choriomeningitis virus segment L	NC_004291	6680	99	S169-(-)ssRNA-30
			Lymphocytic choriomeningitis virus segment S	NC_004294	3376	94	
—	—	Deltavirus	Hepatitis delta virus	NC_001653	1682	59	S170-(-)ssRNA-31
Leviviridae	—	Levivirus	Enterobacteria phage MS2	NC_001417	3569	90	S171-(+)ssRNA-1
		Allolevivirus	Enterobacteria phage Qβ	NC_001890	4215	95	S172-(+)ssRNA-2
Narnaviridae	—	Narnavirus	Saccharomyces 20S RNA narnavirus	NC_004051	2514	99	S173-(+)ssRNA-3
		Mitovirus	Cryphonectria parasitica mitovirus 1-NB631	NC_004046	2728	89	S174-(+)ssRNA-4
Picornaviridae	—	Enterovirus	Poliovirus	NC_002058	7440	89	S175-(+)ssRNA-5
		Rhinovirus	Human rhinovirus 89	NC_001617	7152	90	S176-(+)ssRNA-6
		Hepatovirus	Hepatitis A virus	NC_001489	7478	89	S177-(+)ssRNA-7
		Cardiovirus	Encephalomyocarditis virus	NC_001479	7835	87	S178-(+)ssRNA-8
		Aphthovirus	Foot-and-mouth disease virus - type A	NC_011450	8161	85	S179-(+)ssRNA-9
		Parechovirus	Human parechovirus	NC_001897	7348	89	S180-(+)ssRNA-10
		Erbovirus	Equine rhinitis B virus 1	NC_003983	8828	88	S181-(+)ssRNA-11
		Kobuvirus	Aichi virus	NC_001918	8251	88	S182-(+)ssRNA-12
		Teschovirus	Porcine teschovirus 1	NC_003985	7117	94	S183-(+)ssRNA-13
—	—	Iflavirus	Infectious flacherie virus	NC_003781	9650	95	S184-(+)ssRNA-14
Dicistroviridae	—	Cripavirus	Cricket paralysis virus	NC_003924	9185	87	S185-(+)ssRNA-15
Marnaviridae	—	Marnavirus	Heterosigma akashiwo RNA virus	NC_005281	8587	90	S186-(+)ssRNA-16
Sequiviridae	—	Sequivirus	Parsnip yellow fleck virus	NC_003628	9871	92	S187-(+)ssRNA-17
		Waikavirus	Rice tungro spherical virus	NC_001632	12226	85	S188-(+)ssRNA-18

—	—	Sadwavirus	Satsuma dwarf virus RNA 1	NC_003785	6794	91	S189-(+)ssRNA-19
			Satsuma dwarf virus RNA 2	NC_003786	5344	88	
—	—	Cheravirus	Cherry rasp leaf virus	NC_006271	7034	96	S190-(+)ssRNA-20
			Cherry rasp leaf virus RNA 2	NC_006272	3315	86	
Comoviridae	—	Comovirus	Cowpea mosaic virus RNA 1	NC_003549	5889	95	S191-(+)ssRNA-21
			Cowpea mosaic virus RNA 2	NC_003550	3481	91	
		Fabavirus	Broad bean wilt virus 1 RNA 1	NC_005289	5817	95	S192-(+)ssRNA-22
			Broad bean wilt virus 1 RNA 2	NC_005290	3446	88	
		Nepovirus	Tobacco ringspot virus RNA 1	NC_005097	7514	92	S193-(+)ssRNA-23
			Tobacco ringspot virus RNA 2	NC_005096	3929	84	
Potyviridae	—	Potyvirus	Potato virus Y	NC_001616	9704	94	S194-(+)ssRNA-24
		Rymovirus	Ryegrass mosaic virus	NC_001814	9535	97	S195-(+)ssRNA-25
		Bymovirus	Barley yellow mosaic virus RNA 1	NC_002990	7637	94	S196-(+)ssRNA-26
			Barley yellow mosaic virus RNA 2	NC_002991	3582	74	
		Ipomovirus	Sweet potato mild mottle virus	NC_003797	10818	95	S197-(+)ssRNA-27
		Tritimovirus	Wheat streak mosaic virus	NC_001886	9384	97	S198-(+)ssRNA-28
Caliciviridae	—	Vesivirus	Vesicular exanthema of swine virus	NC_002551	8284	97	S199-(+)ssRNA-29
		Lagovirus	Rabbit hemorrhagic disease virus	NC_001543	7437	99	S200-(+)ssRNA-30
		Norovirus	Norwalk virus	NC_001959	7654	99	S201-(+)ssRNA-31
		Sapovirus	Sapovirus C12	NC_006554	7476	98	S202-(+)ssRNA-32
—	—	Hepevirus	Hepatitis E virus	NC_001434	7176	98	S203-(+)ssRNA-33
Astroviridae	—	Mamastrovirus	Human astrovirus	NC_001943	6813	97	S204-(+)ssRNA-34
		Astrovirius	Turkey astrovirus	NC_002470	7003	97	S205-(+)ssRNA-35
Nodaviridae	—	Alphanodavirus	Nodamura virus RNA1	NC_002690	3204	97	S206-(+)ssRNA-36
			Nodamura virus RNA2	NC_002691	1336	89	
		Betanodavirus	Striped Jack nervous necrosis virus RNA 2	NC_003449	1421	71	S207-(+)ssRNA-37
			Striped Jack nervous necrosis virus RNA 1	NC_003448	3107	95	
Tetraviridae	—	Betatetravirus	Nudaurelia capensis β virus	NC_001990	6625	87	S208-(+)ssRNA-38
—	—	Sobemovirus	Southern bean mosaic virus	NC_001625	4194	95	S209-(+)ssRNA-39
Luteoviridae	—	Luteovirus	Barley yellow dwarf virus - PAV	NC_004750	5677	86	S210-(+)ssRNA-40
		Polerovirus	Potato leafroll virus	NC_001747	5987	91	S211-(+)ssRNA-41
		Enamovirus	Pea enation mosaic virus-1	NC_003629	5706	89	S212-(+)ssRNA-42
Tombusviridae	—	Tombusvirus	Tomato bushy stunt virus	NC_001554	4776	87	S213-(+)ssRNA-43
		Carmovirus	Carnation mottle virus	NC_001265	4003	91	S214-(+)ssRNA-44
		Necrovirus	Tobacco necrosis virus A	NC_001777	3684	96	S215-(+)ssRNA-45
		Dianthovirus	Carnation ringspot virus RNA 1	NC_003530	3840	95	S216-(+)ssRNA-46
			Carnation ringspot virus RNA 2	NC_003531	1403	65	
		Machlomovirus	Maize chlorotic mottle virus	NC_003627	4437	89	S217-(+)ssRNA-47
		Avenavirus	Oat chlorotic stunt virus	NC_003633	4114	88	S218-(+)ssRNA-48
		Aureusvirus	Pothos latent virus	NC_000939	4354	93	S219-(+)ssRNA-49
		Panicovirus	Panicum mosaic virus	NC_002598	4326	91	S220-(+)ssRNA-50

Arteriviridae	—	Arterivirus	Equine arteritis virus	NC_002532	12704	97	S221-(+)ssRNA-51
Coronaviridae	—	Coronavirus	Avian infectious bronchitis virus	NC_001451	27608	95	S222-(+)ssRNA-52
		Torovirus	Breda virus	NC_007447	28475	96	S223-(+)ssRNA-53
Roniviridae	—	Okavirus	Gill-associated virus	NC_010306	26253	97	S224-(+)ssRNA-54
Flaviviridae	—	Flavivirus	Yellow fever virus	NC_002031	10862	94	S225-(+)ssRNA-55
		Pestivirus	Bovine viral diarrhea virus 1	NC_001461	12573	95	S226-(+)ssRNA-56
		Hepacivirus	Hepatitis C virus genotype 1	NC_004102	9646	93	S227-(+)ssRNA-57
Togaviridae	—	Alphavirus	Sindbis virus	NC_001547	11703	96	S228-(+)ssRNA-58
		Rubivirus	Rubella virus	NC_001545	9755	99	S229-(+)ssRNA-59
—	—	Tobamovirus	Tobacco mosaic virus	NC_001367	6395	95	S230-(+)ssRNA-60
—	—	Tobravirus	Tobacco rattle virus RNA 1	NC_003805	6791	92	S231-(+)ssRNA-61
			Tobacco rattle virus RNA 2	NC_003811	3855	66	
—	—	Hordeivirus	Barley stripe mosaic virus RNA 1	NC_003469	3768	90	S232-(+)ssRNA-62
			Barley stripe mosaic virus RNA 2	NC_003481	3289	85	
			Barley stripe mosaic virus RNA 3	NC_003478	3164	87	
—	—	Furovirus	Soil-borne wheat mosaic virus RNA1	NC_002041	7099	91	S233-(+)ssRNA-63
			Soil-borne wheat mosaic virus RNA2	NC_002042	3593	77	
—	—	Pomovirus	Potato mop-top virus RNA 1	NC_003723	6043	90	S234-(+)ssRNA-64
			Potato mop-top virus RNA 2	NC_003725	2964	77	
			Potato mop-top virus RNA 3	NC_003724	3134	79	
—	—	Pecluvirus	Peanut clump virus RNA 1	NC_003672	5897	91	S235-(+)ssRNA-65
			Peanut clump virus RNA 2	NC_003668	4504	82	
—	—	Benyvirus	Beet necrotic yellow vein virus RNA 1	NC_003514	6746	93	S236-(+)ssRNA-66
			Beet necrotic yellow vein virus RNA 2	NC_003515	4609	92	
			Beet necrotic yellow vein virus RNA 3	NC_003516	1774	37	
			Beet necrotic yellow vein virus RNA 4	NC_003517	1465	57	
			Beet necrotic yellow vein virus RNA 5	NC_003513	1320	52	
Bromoviridae	—	Alfamovirus	Alfalfa mosaic virus RNA 1	NC_001495	3644	92	S237-(+)ssRNA-67
			Alfalfa mosaic virus RNA 2	NC_002024	2593	91	
			Alfalfa mosaic virus RNA 3	NC_002025	2037	77	
		Ilarvirus	Tobacco streak virus RNA 1	NC_003844	3491	94	S238-(+)ssRNA-68
			Tobacco streak virus RNA 2	NC_003842	2926	93	
			Tobacco streak virus RNA 3	NC_003845	2205	71	
		Bromovirus	Brome mosaic virus RNA 1	NC_002026	3234	89	S239-(+)ssRNA-69
			Brome mosaic virus RNA 2	NC_002027	2865	86	
			Brome mosaic virus RNA 3	NC_002028	2111	70	

		Cucumovirus	Cucumber mosaic virus RNA 1	NC_002034	3357	88	S240-(+)ssRNA-70
			Cucumber mosaic virus RNA 2	NC_002035	3050	87	
			Cucumber mosaic virus RNA 3	NC_001440	2216	67	
		Oleavirus	Olive latent virus 2 RNA 1	NC_003673	3128	87	S241-(+)ssRNA-71
			Olive latent virus 2 RNA 2	NC_003674	2735	86	
			Olive latent virus 2 RNA 3	NC_003671	2438	64	
—	—	Ourmiavirus	Ourmia melon virus RNA 1	NC_011068	2814	91	S242-(+)ssRNA-72
			Ourmia melon virus RNA 2	NC_011069	1064	81	
			Ourmia melon virus RNA 3	NC_011070	974	64	
—	—	Idaeovirus	Raspberry bushy dwarf virus RNA 1	NC_003739	5449	93	S243-(+)ssRNA-73
			Raspberry bushy dwarf virus RNA 2	NC_003740	2231	85	
Tymoviridae	—	Maculavirus	Grapevine fleck virus	NC_003347	7564	92	S244-(+)ssRNA-74
		Tymovirus	Turnip yellow mosaic virus	NC_004063	6318	96	S245-(+)ssRNA-75
		Marafivirus	Maize rayado fino virus	NC_002786	6305	96	S246-(+)ssRNA-76
Closteroviridae	—	Closterovirus	Beet yellows virus	NC_001598	15480	97	S247-(+)ssRNA-77
		Crinivirus	Lettuce infectious yellows virus RNA 1	NC_003617	8118	96	S248-(+)ssRNA-78
			Lettuce infectious yellows virus RNA 2	NC_003618	7193	83	
		Ampelovirus	Grapevine leafroll-associated virus 3	NC_004667	17919	89	S249-(+)ssRNA-79
Flexiviridae	—	Potexvirus	Potato virus X	NC_011620	6435	96	S250-(+)ssRNA-80
		Mandarivirus	Indian citrus ringspot virus	NC_003093	7560	98	S251-(+)ssRNA-81
		Allexivirus	Shallot virus X	NC_003795	8832	96	S252-(+)ssRNA-82
		Foveavirus	Apple stem pitting virus	NC_003462	9306	96	S253-(+)ssRNA-83
		Capillovirus	Apple stem grooving virus	NC_001749	6495	97	S254-(+)ssRNA-84
		Vitivirus	Grapevine virus A	NC_003604	7351	97	S255-(+)ssRNA-85
		Trichovirus	Apple chlorotic leaf spot virus	NC_001409	7555	95	S256-(+)ssRNA-86
Barnaviridae	—	Barnavirus	Mushroom bacilliform virus	NC_001633	4009	91	S257-(+)ssRNA-87
