# Supplementary material for: Coevolution between simple sequence repeats (SSRs) and virus genome size
Source: BMC Genomics. 2012 Aug 30;13:435. doi: 10.1186/1471-2164-13-435 (PMC3585866; doi:10.1186/1471-2164-13-435)
Supplement: Additional file 2 — Occurrence of SSRs in analyzed virus genomes. [file 1471-2164-13-435-S2.pdf]

Additional file 2 Occurrence of SSRs in analyzed virus genomes

No. Type	Genome size (bp)	Mono-	Di-	Tri-	Tetra-	Penta-	Hexa-	Total
S1-dsDNA-1	168903	191	211	67	1	1	1	472
S2-dsDNA-2	94800	139	129	33	0	0	0	301
S3-dsDNA-3	33593	73	61	15	1	0	0	150
S4-dsDNA-4	36717	55	36	8	0	0	0	99
S5-dsDNA-5	132562	149	255	37	0	0	0	441
S6-dsDNA-6	48502	83	72	19	0	0	0	174
S7-dsDNA-7	48836	66	63	16	1	0	0	146
S8-dsDNA-8	121750	100	203	46	1	0	0	350
S9-dsDNA-9	22172	64	37	15	1	0	0	117
S10-dsDNA-10	52297	13	46	27	1	0	0	87
S11-dsDNA-11	46375	72	59	18	0	1	0	150
S12-dsDNA-12	41491	11	102	20	0	0	0	133
S13-dsDNA-13	39937	1	62	12	0	0	0	75
S14-dsDNA-14	19282	5	35	6	0	0	0	46
S15-dsDNA-15	41724	46	53	21	0	0	0	120
S16-dsDNA-16	70153	25	61	38	2	0	0	126
S17-dsDNA-17	14927	15	4	8	0	0	0	27
S18-dsDNA-18	10079	36	31	8	0	0	0	75
S19-dsDNA-19	11965	21	18	3	0	0	0	42
S20-dsDNA-20	40900	128	108	18	0	0	0	254
S21-dsDNA-21	20869	44	64	7	1	0	0	116
S22-dsDNA-22	35450	193	128	19	0	2	0	342
S23-dsDNA-23	15465	20	36	3	0	0	0	59
S24-dsDNA-24	14462	5	29	0	1	0	0	35
S25-dsDNA-25	194711	495	676	108	5	1	2	1287
S26-dsDNA-26	139962	50	858	173	20	0	2	1103
S27-dsDNA-27	288539	724	988	169	10	1	0	1892
S28-dsDNA-28	149955	1107	492	115	8	1	1	1724
S29-dsDNA-29	161773	295	384	60	4	0	0	743
S30-dsDNA-30	146454	536	752	120	8	1	3	1420
S31-dsDNA-31	190289	123	1349	157	3	2	3	1637
S32-dsDNA-32	134721	836	337	66	2	0	0	1241
S33-dsDNA-33	232392	1383	1855	554	22	6	3	3823
S34-dsDNA-34	170101	787	307	80	6	0	0	1180
S35-dsDNA-35	212482	1054	417	186	7	2	1	1667
S36-dsDNA-36	191100	286	220	122	6	1	1	636
S37-dsDNA-37	105903	90	259	64	0	0	0	413
S38-dsDNA-38	102653	405	205	62	1	2	2	677
S39-dsDNA-39	111362	64	403	78	1	0	0	546

S40-dsDNA-40	330743	822	746	141	2	4	2	1717
S41-dsDNA-41	335593	489	999	306	11	1	19	1825
S42-dsDNA-42	407339	768	998	388	13	0	3	2170
S43-dsDNA-43	133894	306	309	101	4	2	3	725
S44-dsDNA-44	123500	206	436	148	9	1	1	801
S45-dsDNA-45	305107	522	641	555	5	0	8	1731
S46-dsDNA-46	134226	282	409	78	4	3	2	778
S47-dsDNA-47	152261	663	468	161	19	4	4	1319
S48-dsDNA-48	124884	283	315	39	5	2	1	645
S49-dsDNA-49	177874	348	442	56	5	2	26	879
S50-dsDNA-50	148687	245	366	60	3	0	0	674
S51-dsDNA-51	235646	343	685	296	2	0	0	1326
S52-dsDNA-52	230278	239	680	355	18	0	5	1297
S53-dsDNA-53	159322	497	430	58	1	0	27	1013
S54-dsDNA-54	172764	420	334	132	5	0	0	891
S55-dsDNA-55	112930	354	309	67	2	0	0	732
S56-dsDNA-56	35937	68	95	42	0	0	1	206
S57-dsDNA-57	43804	47	108	36	0	0	0	191
S58-dsDNA-58	29576	112	45	11	0	0	0	168
S59-dsDNA-59	26163	73	45	11	0	0	0	129
S60-dsDNA-60	5243	21	6	4	0	0	0	31
S61-dsDNA-61	7961	15	32	7	0	0	0	54
S62-dsDNA-62	7746	16	18	3	0	0	0	37
S63-dsDNA-63	7353	9	15	6	0	0	0	30
S64-dsDNA-64	8095	13	15	1	0	0	0	29
S65-dsDNA-65	7841	20	15	6	0	0	0	41
S66-dsDNA-66	7610	27	14	5	0	0	0	46
S67-dsDNA-67	7729	8	8	4	0	0	0	20
S68-dsDNA-68	7304	6	16	3	0	0	0	25
S69-dsDNA-69	7687	10	15	4	0	0	0	29
S70-dsDNA-70	7868	17	10	4	0	0	0	31
S71-dsDNA-71	8607	31	23	5	1	0	0	60
S72-dsDNA-72	7815	11	18	4	0	0	0	33
S73-dsDNA-73	7614	11	14	7	0	0	0	32
S74-dsDNA-74	7276	11	14	7	0	0	0	32
S75-dsDNA-75	7879	11	25	6	0	0	0	42
S76-dsDNA-76	246734	469	495	114	17	3	1	1099
S77-dsDNA-77	156922	78	354	121	6	0	0	559
S78-ssDNA-1	6407	6	7	5	0	0	0	18
S79-ssDNA-2	4491	16	16	5	0	0	0	37
S80-ssDNA-3	5386	4	4	0	0	0	0	8
S81-ssDNA-4	4421	15	8	1	0	0	0	24
S82-ssDNA-5	4594	15	7	4	0	0	0	26

S83-ssDNA-6	4877	6	4	5	0	0	0	15
S84-ssDNA-7	2690	3	5	2	0	0	0	10
S85-ssDNA-8	2994	4	6	5	0	0	0	15
S86-ssDNA-9	5232	4	11	2	0	0	0	17
S87-ssDNA-10	2861	5	7	3	0	0	0	15
S88-ssDNA-11	1758	4	2	2	1	0	0	9
S89-ssDNA-12	2319	17	7	1	0	0	0	25
S90-ssDNA-13	3852	22	13	6	0	0	0	41
S91-ssDNA-14	8024	2	28	10	0	1	0	41
S92-ssDNA-15	6396	7	19	9	0	0	0	35
S93-ssDNA-16	5149	12	12	3	0	0	0	27
S94-ssDNA-17	5594	16	14	2	0	0	0	32
S95-ssDNA-18	4679	2	16	5	4	0	0	27
S96-ssDNA-19	4801	7	17	6	0	0	0	30
S97-ssDNA-20	5517	14	28	5	0	0	0	47
S98-ssDNA-21	5908	7	11	8	0	0	0	26
S99-ssDNA-22	5078	16	18	8	1	0	0	43
S100-ssDNA-23	3776	9	11	4	0	0	0	24
S101-ssDNA-24	5454	6	9	7	0	0	0	22
S102-dsDNA-RT-1	3215	2	7	2	0	0	0	11
S103-dsDNA-RT-2	3027	0	5	4	0	0	0	9
S104-dsDNA-RT-3	8024	8	15	7	0	0	0	30
S105-dsDNA-RT-4	8178	32	12	4	0	0	0	48
S106-dsDNA-RT-5	8159	36	27	11	1	0	0	75
S107-dsDNA-RT-6	8002	16	21	4	0	0	0	41
S108-dsDNA-RT-7	7489	1	10	7	0	0	0	18
S109-dsDNA-RT-8	7206	13	10	4	0	0	0	27
S110-ssRNA-RT-1	8805	12	16	3	1	0	0	32
S111-ssRNA-RT-2	8282	11	16	2	0	0	0	29
S112-ssRNA-RT-3	7286	5	23	3	0	0	0	31
S113-ssRNA-RT-4	8419	22	26	2	0	0	0	50
S114-ssRNA-RT-5	9181	21	32	12	0	0	0	65
S115-ssRNA-RT-6	12708	20	22	7	1	0	0	50
S116-ssRNA-RT-7	13246	12	30	10	0	0	1	53
S117-dsRNA-1	13385	4	29	5	0	0	0	38
S118-dsRNA-2	23564	6	47	8	0	0	0	61
S119-dsRNA-3	19208	21	45	11	0	0	0	77
S120-dsRNA-4	17448	27	39	8	0	0	0	74
S121-dsRNA-5	29174	16	63	16	1	0	0	96
S122-dsRNA-6	23015	15	43	9	0	0	0	67
S123-dsRNA-7	24732	1	64	7	0	0	0	72
S124-dsRNA-8	29339	55	52	18	0	0	0	125
S125-dsRNA-9	25709	16	47	13	0	0	0	76

S126-dsRNA-10	26164	4	61	9	0	0	0	74
S127-dsRNA-11	20682	5	30	10	0	0	0	45
S128-dsRNA-12	23433	4	46	8	1	0	0	59
S129-dsRNA-13	5881	5	13	3	0	0	0	21
S130-dsRNA-14	5898	1	10	2	0	0	0	13
S131-dsRNA-15	6603	7	10	1	0	0	0	18
S132-dsRNA-16	4579	1	9	1	0	0	0	11
S133-dsRNA-17	6277	7	12	2	0	0	0	21
S134-dsRNA-18	5284	6	14	3	0	0	0	23
S135-dsRNA-19	6105	10	12	3	0	0	0	25
S136-dsRNA-20	3663	2	8	1	0	0	0	11
S137-dsRNA-21	12640	17	23	15	0	0	0	55
S138-dsRNA-22	12734	2	18	5	0	0	0	25
S139-dsRNA-23	17635	23	41	9	0	0	0	73
S140-(-)ssRNA-1	8910	10	17	2	0	0	0	29
S141-(-)ssRNA-2	11161	19	25	3	0	0	0	47
S142-(-)ssRNA-3	11932	21	34	2	0	0	0	57
S143-(-)ssRNA-4	14900	41	37	8	1	0	0	87
S144-(-)ssRNA-5	12807	9	38	8	0	0	0	55
S145-(-)ssRNA-6	12020	6	28	9	0	0	0	43
S146-(-)ssRNA-7	11131	20	22	11	0	0	0	53
S147-(-)ssRNA-8	19111	23	37	3	0	0	0	63
S148-(-)ssRNA-9	18959	26	38	6	2	0	0	72
S149-(-)ssRNA-10	15384	5	38	4	0	0	0	47
S150-(-)ssRNA-11	15894	24	27	4	0	0	0	55
S151-(-)ssRNA-12	15384	20	39	1	0	0	0	60
S152-(-)ssRNA-13	18234	15	44	9	0	0	0	68
S153-(-)ssRNA-14	15186	22	37	4	0	0	0	63
S154-(-)ssRNA-15	15225	37	38	5	0	0	0	80
S155-(-)ssRNA-16	14071	27	44	10	0	0	0	81
S156-(-)ssRNA-17	12878	17	49	8	0	0	0	74
S157-(-)ssRNA-18	11278	31	32	9	0	0	0	72
S158-(-)ssRNA-19	13460	8	39	6	0	0	0	53
S159-(-)ssRNA-20	12555	20	30	6	0	0	0	56
S160-(-)ssRNA-21	10461	9	37	4	0	0	0	50
S161-(-)ssRNA-22	14452	15	22	9	1	0	0	47
S162-(-)ssRNA-23	12716	7	34	10	1	0	0	52
S163-(-)ssRNA-24	12294	17	37	11	1	0	0	66
S164-(-)ssRNA-25	11845	19	34	15	1	0	0	69
S165-(-)ssRNA-26	18859	40	38	9	0	0	0	87
S166-(-)ssRNA-27	11979	8	36	13	0	0	0	57
S167-(-)ssRNA-28	16634	59	37	8	0	2	0	106
S168-(-)ssRNA-29	17145	28	62	6	0	0	0	96

S169-(-)ssRNA-30	10056	10	18	1	0	0	0	29
S170-(-)ssRNA-31	1682	6	5	1	0	0	0	12
S171-(+)ssRNA-1	3569	2	6	0	0	0	0	8
S172-(+)ssRNA-2	4215	2	4	2	0	0	0	8
S173-(+)ssRNA-3	2514	3	3	1	0	0	0	7
S174-(+)ssRNA-4	2728	0	7	0	0	0	0	7
S175-(+)ssRNA-5	7440	3	15	3	0	0	0	21
S176-(+)ssRNA-6	7152	2	23	1	0	0	0	26
S177-(+)ssRNA-7	7478	9	9	4	0	0	0	22
S178-(+)ssRNA-8	7835	8	18	4	0	0	0	30
S179-(+)ssRNA-9	8161	2	23	3	0	0	0	28
S180-(+)ssRNA-10	7348	6	12	0	0	0	0	18
S181-(+)ssRNA-11	8828	3	16	1	0	0	0	20
S182-(+)ssRNA-12	8251	16	23	2	3	0	0	44
S183-(+)ssRNA-13	7117	2	16	2	0	0	0	20
S184-(+)ssRNA-14	9650	11	25	3	0	0	0	39
S185-(+)ssRNA-15	9185	4	26	6	0	0	0	36
S186-(+)ssRNA-16	8587	6	16	4	0	0	0	26
S187-(+)ssRNA-17	9871	4	28	3	0	0	0	35
S188-(+)ssRNA-18	12226	3	32	1	0	0	0	36
S189-(+)ssRNA-19	12138	11	39	7	0	0	0	57
S190-(+)ssRNA-20	10349	12	29	5	0	0	0	46
S191-(+)ssRNA-21	9370	2	18	3	0	0	0	23
S192-(+)ssRNA-22	9263	9	23	1	0	0	0	33
S193-(+)ssRNA-23	11443	8	30	3	2	0	0	43
S194-(+)ssRNA-24	9704	1	32	2	0	0	1	36
S195-(+)ssRNA-25	9535	1	21	3	0	0	0	25
S196-(+)ssRNA-26	11219	4	34	8	0	2	0	48
S197-(+)ssRNA-27	10818	6	16	5	0	0	0	27
S198-(+)ssRNA-28	9384	1	16	2	0	0	0	19
S199-(+)ssRNA-29	8284	5	12	1	0	0	0	18
S200-(+)ssRNA-30	7437	1	15	1	0	0	0	17
S201-(+)ssRNA-31	7654	8	15	5	0	0	0	28
S202-(+)ssRNA-32	7476	3	17	2	1	0	0	23
S203-(+)ssRNA-33	7176	5	11	6	0	0	0	22
S204-(+)ssRNA-34	6813	9	13	3	0	0	0	25
S205-(+)ssRNA-35	7003	11	19	4	0	0	0	34
S206-(+)ssRNA-36	4540	0	3	1	0	0	0	4
S207-(+)ssRNA-37	4528	1	5	2	0	0	0	8
S208-(+)ssRNA-38	6625	5	10	5	0	0	0	20
S209-(+)ssRNA-39	4194	2	11	2	0	0	0	15
S210-(+)ssRNA-40	5677	5	13	3	1	0	0	22
S211-(+)ssRNA-41	5987	1	10	3	0	0	0	14

S212-(+)ssRNA-42	5706	5	5	2	0	0	0	12
S213-(+)ssRNA-43	4776	2	15	1	0	0	0	18
S214-(+)ssRNA-44	4003	4	12	0	0	0	0	16
S215-(+)ssRNA-45	3684	4	5	4	0	0	0	13
S216-(+)ssRNA-46	5243	1	9	0	0	0	0	10
S217-(+)ssRNA-47	4437	2	13	1	0	0	0	16
S218-(+)ssRNA-48	4114	1	10	0	0	0	0	11
S219-(+)ssRNA-49	4354	4	10	3	0	0	0	17
S220-(+)ssRNA-50	4326	4	9	3	0	0	0	16
S221-(+)ssRNA-51	12704	1	28	11	0	0	0	40
S222-(+)ssRNA-52	27608	10	66	14	0	0	0	90
S223-(+)ssRNA-53	28475	39	49	14	0	0	0	102
S224-(+)ssRNA-54	26253	4	93	14	0	0	0	111
S225-(+)ssRNA-55	10862	10	29	5	0	0	0	44
S226-(+)ssRNA-56	12573	19	31	10	0	0	0	60
S227-(+)ssRNA-57	9646	20	16	4	0	0	0	40
S228-(+)ssRNA-58	11703	8	17	5	1	0	0	31
S229-(+)ssRNA-59	9755	15	47	9	0	0	0	71
S230-(+)ssRNA-60	6395	8	21	3	0	0	0	32
S231-(+)ssRNA-61	10646	9	25	7	0	0	0	41
S232-(+)ssRNA-62	10221	6	15	7	0	0	0	28
S233-(+)ssRNA-63	10692	10	23	10	0	0	0	43
S234-(+)ssRNA-64	12141	22	23	9	0	0	0	54
S235-(+)ssRNA-65	10401	11	33	11	0	0	0	55
S236-(+)ssRNA-66	15914	4	33	17	0	0	1	55
S237-(+)ssRNA-67	8274	6	16	2	0	0	0	24
S238-(+)ssRNA-68	8622	4	24	2	0	0	0	30
S239-(+)ssRNA-69	8210	3	24	6	0	0	0	33
S240-(+)ssRNA-70	8623	4	22	5	0	0	0	31
S241-(+)ssRNA-71	8301	10	13	3	0	0	0	26
S242-(+)ssRNA-72	4852	3	5	3	0	0	0	11
S243-(+)ssRNA-73	7680	3	21	2	0	0	0	26
S244-(+)ssRNA-74	7564	33	10	11	0	0	0	54
S245-(+)ssRNA-75	6318	8	10	1	0	0	0	19
S246-(+)ssRNA-76	6305	4	12	3	0	0	0	19
S247-(+)ssRNA-77	15480	12	32	6	0	0	0	50
S248-(+)ssRNA-78	15311	11	36	7	1	0	0	55
S249-(+)ssRNA-79	17919	16	34	8	0	0	0	58
S250-(+)ssRNA-80	6435	1	13	4	0	0	0	18
S251-(+)ssRNA-81	7560	1	29	6	1	0	0	37
S252-(+)ssRNA-82	8832	2	20	0	0	0	0	22
S253-(+)ssRNA-83	9306	5	20	4	0	0	0	29
S254-(+)ssRNA-84	6495	11	10	1	1	0	0	23

S255-(+)ssRNA-85	7351	1	14	2	0	0	0	17
S256-(+)ssRNA-86	7555	9	19	3	0	0	0	31
S257-(+)ssRNA-87	4009	2	4	3	0	0	0	9
