# Supplementary material for: Coevolution between simple sequence repeats (SSRs) and virus genome size
Source: BMC Genomics. 2012 Aug 30;13:435. doi: 10.1186/1471-2164-13-435 (PMC3585866; doi:10.1186/1471-2164-13-435)
Supplement: Additional file 3 — Length (bp) of SSRs in analyzed virus genomes. [file 1471-2164-13-435-S3.pdf]

Additional file 3 Length (bp) of SSRs in analyzed virus genomes

No. Type	Genome size (bp)	Mono-	Di-	Tri-	Tetra-	Penta-	Hexa-	Total	% (Total SSRs)
S1-dsDNA-1	168903	1154	1270	603	12	15	18	3072	1.82
S2-dsDNA-2	94800	871	796	300	0	0	0	1967	2.07
S3-dsDNA-3	33593	459	370	135	12	0	0	976	2.91
S4-dsDNA-4	36717	338	222	72	0	0	0	632	1.72
S5-dsDNA-5	132562	977	1568	333	0	0	0	2878	2.17
S6-dsDNA-6	48502	517	440	174	0	0	0	1131	2.33
S7-dsDNA-7	48836	416	390	144	12	0	0	962	1.97
S8-dsDNA-8	121750	601	1228	417	12	0	0	2258	1.85
S9-dsDNA-9	22172	411	224	141	12	0	0	788	3.55
S10-dsDNA-10	52297	80	284	249	12	0	0	625	1.20
S11-dsDNA-11	46375	456	356	165	0	15	0	992	2.14
S12-dsDNA-12	41491	67	620	186	0	0	0	873	2.10
S13-dsDNA-13	39937	6	376	108	0	0	0	490	1.23
S14-dsDNA-14	19282	30	210	54	0	0	0	294	1.52
S15-dsDNA-15	41724	291	332	189	0	0	0	812	1.95
S16-dsDNA-16	70153	158	366	342	24	0	0	890	1.27
S17-dsDNA-17	14927	94	24	78	0	0	0	196	1.31
S18-dsDNA-18	10079	230	188	72	0	0	0	490	4.86
S19-dsDNA-19	11965	136	116	27	0	0	0	279	2.33
S20-dsDNA-20	40900	822	672	165	0	0	0	1659	4.06
S21-dsDNA-21	20869	278	396	63	12	0	0	749	3.59
S22-dsDNA-22	35450	1261	792	174	0	30	0	2257	6.37
S23-dsDNA-23	15465	123	230	27	0	0	0	380	2.46
S24-dsDNA-24	14462	30	180	0	12	0	0	222	1.54
S25-dsDNA-25	194711	3178	4144	990	60	15	36	8423	4.33
S26-dsDNA-26	139962	313	5556	1572	248	0	36	7725	5.52
S27-dsDNA-27	288539	4641	6146	1536	120	15	0	12458	4.32
S28-dsDNA-28	149955	7502	3084	1065	96	15	18	11780	7.86
S29-dsDNA-29	161773	1907	2368	564	48	0	0	4887	3.02
S30-dsDNA-30	146454	3520	4776	1110	96	25	78	9605	6.56
S31-dsDNA-31	190289	868	9472	1455	36	30	54	11915	6.26
S32-dsDNA-32	134721	5585	2096	615	24	0	0	8320	6.18
S33-dsDNA-33	232392	9293	11926	5202	264	90	54	26829	11.54
S34-dsDNA-34	170101	5351	1888	732	72	0	0	8043	4.73
S35-dsDNA-35	212482	6814	2588	1707	84	30	24	11247	5.29
S36-dsDNA-36	191100	1818	1342	1116	72	15	18	4381	2.29
S37-dsDNA-37	105903	585	1674	579	0	0	0	2838	2.68
S38-dsDNA-38	102653	2590	1264	567	12	30	42	4505	4.39
S39-dsDNA-39	111362	403	2508	759	12	0	0	3682	3.31

S40-dsDNA-40	330743	5386	4598	1296	56	60	36	11432	3.46
S41-dsDNA-41	335593	3264	6276	3108	132	15	432	13227	3.94
S42-dsDNA-42	407339	4851	6210	3642	156	0	54	14913	3.66
S43-dsDNA-43	133894	1932	1902	981	48	30	54	4947	3.69
S44-dsDNA-44	123500	1336	2706	1413	116	15	24	5610	4.54
S45-dsDNA-45	305107	3299	3936	5892	64	0	144	13335	4.37
S46-dsDNA-46	134226	1891	2766	759	48	45	54	5563	4.14
S47-dsDNA-47	152261	4389	2970	1548	228	85	132	9352	6.14
S48-dsDNA-48	124884	1902	1986	354	60	30	18	4350	3.48
S49-dsDNA-49	177874	2477	2704	504	60	35	990	6770	3.81
S50-dsDNA-50	148687	1575	2234	543	36	0	0	4388	2.95
S51-dsDNA-51	235646	2340	4260	2763	28	0	0	9391	3.99
S52-dsDNA-52	230278	1561	4256	3393	244	0	96	9550	4.15
S53-dsDNA-53	159322	3364	2694	564	12	0	1158	7792	4.89
S54-dsDNA-54	172764	2643	2072	1209	64	0	0	5988	3.47
S55-dsDNA-55	112930	2267	1904	630	28	0	0	4829	4.28
S56-dsDNA-56	35937	442	596	420	0	0	18	1476	4.11
S57-dsDNA-57	43804	302	668	348	0	0	0	1318	3.01
S58-dsDNA-58	29576	715	276	99	0	0	0	1090	3.69
S59-dsDNA-59	26163	468	270	108	0	0	0	846	3.23
S60-dsDNA-60	5243	133	36	45	0	0	0	214	4.08
S61-dsDNA-61	7961	101	204	66	0	0	0	371	4.66
S62-dsDNA-62	7746	104	118	36	0	0	0	258	3.33
S63-dsDNA-63	7353	57	92	57	0	0	0	206	2.80
S64-dsDNA-64	8095	89	92	9	0	0	0	190	2.35
S65-dsDNA-65	7841	134	90	63	0	0	0	287	3.66
S66-dsDNA-66	7610	175	86	48	0	0	0	309	4.06
S67-dsDNA-67	7729	55	52	36	0	0	0	143	1.85
S68-dsDNA-68	7304	36	102	27	0	0	0	165	2.26
S69-dsDNA-69	7687	68	90	39	0	0	0	197	2.56
S70-dsDNA-70	7868	112	60	39	0	0	0	211	2.68
S71-dsDNA-71	8607	240	142	60	12	0	0	454	5.27
S72-dsDNA-72	7815	73	110	36	0	0	0	219	2.80
S73-dsDNA-73	7614	69	84	66	0	0	0	219	2.88
S74-dsDNA-74	7276	71	86	69	0	0	0	226	3.11
S75-dsDNA-75	7879	78	156	54	0	0	0	288	3.66
S76-dsDNA-76	246734	3167	3076	1050	216	50	18	7577	3.07
S77-dsDNA-77	156922	554	2238	1317	72	0	0	4181	2.66
S78-ssDNA-1	6407	39	42	45	0	0	0	126	1.97
S79-ssDNA-2	4491	109	102	45	0	0	0	256	5.70
S80-ssDNA-3	5386	25	24	0	0	0	0	49	0.91
S81-ssDNA-4	4421	100	50	9	0	0	0	159	3.60
S82-ssDNA-5	4594	97	42	39	0	0	0	178	3.87

S83-ssDNA-6	4877	44	26	45	0	0	0	115	2.36
S84-ssDNA-7	2690	19	30	18	0	0	0	67	2.49
S85-ssDNA-8	2994	25	36	45	0	0	0	106	3.54
S86-ssDNA-9	5232	24	74	18	0	0	0	116	2.22
S87-ssDNA-10	2861	30	42	27	0	0	0	99	3.46
S88-ssDNA-11	1758	25	12	18	12	0	0	67	3.81
S89-ssDNA-12	2319	115	42	9	0	0	0	166	7.16
S90-ssDNA-13	3852	151	80	54	0	0	0	285	7.40
S91-ssDNA-14	8024	14	190	93	0	15	0	312	3.89
S92-ssDNA-15	6396	45	124	81	0	0	0	250	3.91
S93-ssDNA-16	5149	76	76	27	0	0	0	179	3.48
S94-ssDNA-17	5594	99	88	18	0	0	0	205	3.66
S95-ssDNA-18	4679	12	98	48	48	0	0	206	4.40
S96-ssDNA-19	4801	45	110	63	0	0	0	218	4.54
S97-ssDNA-20	5517	89	174	45	0	0	0	308	5.58
S98-ssDNA-21	5908	45	66	78	0	0	0	189	3.20
S99-ssDNA-22	5078	100	108	72	12	0	0	292	5.75
S100-ssDNA-23	3776	58	72	36	0	0	0	166	4.40
S101-ssDNA-24	5454	39	56	75	0	0	0	170	3.12
S102-dsDNA-RT-1	3215	12	42	18	0	0	0	72	2.24
S103-dsDNA-RT-2	3027	0	32	36	0	0	0	68	2.25
S104-dsDNA-RT-3	8024	49	96	63	0	0	0	208	2.59
S105-dsDNA-RT-4	8178	193	72	36	0	0	0	301	3.68
S106-dsDNA-RT-5	8159	219	174	102	12	0	0	507	6.21
S107-dsDNA-RT-6	8002	96	138	36	0	0	0	270	3.37
S108-dsDNA-RT-7	7489	6	60	63	0	0	0	129	1.72
S109-dsDNA-RT-8	7206	78	62	36	0	0	0	176	2.44
S110-ssRNA-RT-1	8805	72	100	30	12	0	0	214	2.43
S111-ssRNA-RT-2	8282	69	100	18	0	0	0	187	2.26
S112-ssRNA-RT-3	7286	30	142	30	0	0	0	202	2.77
S113-ssRNA-RT-4	8419	146	164	18	0	0	0	328	3.90
S114-ssRNA-RT-5	9181	127	202	111	0	0	0	440	4.79
S115-ssRNA-RT-6	12708	124	146	72	12	0	0	354	2.79
S116-ssRNA-RT-7	13246	72	182	90	0	0	18	362	2.73
S117-dsRNA-1	13385	27	188	45	0	0	0	260	1.94
S118-dsRNA-2	23564	38	292	72	0	0	0	402	1.71
S119-dsRNA-3	19208	129	282	99	0	0	0	510	2.66
S120-dsRNA-4	17448	165	246	72	0	0	0	483	2.77
S121-dsRNA-5	29174	98	392	144	12	0	0	646	2.21
S122-dsRNA-6	23015	96	258	81	0	0	0	435	1.89
S123-dsRNA-7	24732	6	396	63	0	0	0	465	1.88
S124-dsRNA-8	29339	336	322	162	0	0	0	820	2.79
S125-dsRNA-9	25709	98	290	126	0	0	0	514	2.00

S126-dsRNA-10	26164	25	378	84	0	0	0	487	1.86
S127-dsRNA-11	20682	30	188	93	0	0	0	311	1.50
S128-dsRNA-12	23433	28	282	72	12	0	0	394	1.68
S129-dsRNA-13	5881	31	78	27	0	0	0	136	2.31
S130-dsRNA-14	5898	6	60	18	0	0	0	84	1.42
S131-dsRNA-15	6603	44	60	9	0	0	0	113	1.71
S132-dsRNA-16	4579	7	54	9	0	0	0	70	1.53
S133-dsRNA-17	6277	48	76	18	0	0	0	142	2.26
S134-dsRNA-18	5284	36	86	27	0	0	0	149	2.82
S135-dsRNA-19	6105	78	76	27	0	0	0	181	2.96
S136-dsRNA-20	3663	14	48	12	0	0	0	74	2.02
S137-dsRNA-21	12640	109	144	153	0	0	0	406	3.21
S138-dsRNA-22	12734	29	108	45	0	0	0	182	1.43
S139-dsRNA-23	17635	151	260	81	0	0	0	492	2.79
S140-(-)ssRNA-1	8910	63	104	18	0	0	0	185	2.08
S141-(-)ssRNA-2	11161	119	152	30	0	0	0	301	2.70
S142-(-)ssRNA-3	11932	136	212	18	0	0	0	366	3.07
S143-(-)ssRNA-4	14900	267	238	75	12	0	0	592	3.97
S144-(-)ssRNA-5	12807	58	240	78	0	0	0	376	2.94
S145-(-)ssRNA-6	12020	36	180	87	0	0	0	303	2.52
S146-(-)ssRNA-7	11131	131	138	105	0	0	0	374	3.36
S147-(-)ssRNA-8	19111	139	226	27	0	0	0	392	2.05
S148-(-)ssRNA-9	18959	160	228	54	24	0	0	466	2.46
S149-(-)ssRNA-10	15384	30	236	36	0	0	0	302	1.96
S150-(-)ssRNA-11	15894	154	168	36	0	0	0	358	2.25
S151-(-)ssRNA-12	15384	124	238	9	0	0	0	371	2.41
S152-(-)ssRNA-13	18234	92	268	81	0	0	0	441	2.42
S153-(-)ssRNA-14	15186	135	230	36	0	0	0	401	2.64
S154-(-)ssRNA-15	15225	236	238	45	0	0	0	519	3.41
S155-(-)ssRNA-16	14071	168	272	93	0	0	0	533	3.79
S156-(-)ssRNA-17	12878	108	302	72	0	0	0	482	3.74
S157-(-)ssRNA-18	11278	200	200	90	0	0	0	490	4.34
S158-(-)ssRNA-19	13460	49	244	54	0	0	0	347	2.58
S159-(-)ssRNA-20	12555	120	186	54	0	0	0	360	2.87
S160-(-)ssRNA-21	10461	54	232	36	0	0	0	322	3.08
S161-(-)ssRNA-22	14452	91	134	81	12	0	0	318	2.20
S162-(-)ssRNA-23	12716	60	214	93	12	0	0	379	2.98
S163-(-)ssRNA-24	12294	107	234	99	12	0	0	452	3.68
S164-(-)ssRNA-25	11845	121	214	135	12	0	0	482	4.07
S165-(-)ssRNA-26	18859	257	230	81	0	0	0	568	3.01
S166-(-)ssRNA-27	11979	51	226	117	0	0	0	394	3.29
S167-(-)ssRNA-28	16634	367	226	75	0	30	0	698	4.20
S168-(-)ssRNA-29	17145	184	386	54	0	0	0	624	3.64

S169-(-)ssRNA-30	10056	61	110	9	0	0	0	180	1.79
S170-(-)ssRNA-31	1682	36	32	9	0	0	0	77	4.58
S171-(+)ssRNA-1	3569	12	36	0	0	0	0	48	1.34
S172-(+)ssRNA-2	4215	12	26	18	0	0	0	56	1.33
S173-(+)ssRNA-3	2514	18	22	9	0	0	0	49	1.95
S174-(+)ssRNA-4	2728	0	42	0	0	0	0	42	1.54
S175-(+)ssRNA-5	7440	20	92	27	0	0	0	139	1.87
S176-(+)ssRNA-6	7152	12	148	9	0	0	0	169	2.36
S177-(+)ssRNA-7	7478	54	54	36	0	0	0	144	1.93
S178-(+)ssRNA-8	7835	161	110	36	0	0	0	307	3.92
S179-(+)ssRNA-9	8161	27	142	27	0	0	0	196	2.40
S180-(+)ssRNA-10	7348	49	76	0	0	0	0	125	1.70
S181-(+)ssRNA-11	8828	18	100	9	0	0	0	127	1.44
S182-(+)ssRNA-12	8251	100	148	18	36	0	0	302	3.66
S183-(+)ssRNA-13	7117	20	96	18	0	0	0	134	1.88
S184-(+)ssRNA-14	9650	70	156	27	0	0	0	253	2.62
S185-(+)ssRNA-15	9185	25	158	60	0	0	0	243	2.65
S186-(+)ssRNA-16	8587	41	98	36	0	0	0	175	2.04
S187-(+)ssRNA-17	9871	26	176	30	0	0	0	232	2.35
S188-(+)ssRNA-18	12226	65	196	9	0	0	0	270	2.21
S189-(+)ssRNA-19	12138	66	246	63	0	0	0	375	3.09
S190-(+)ssRNA-20	10349	73	178	57	0	0	0	308	2.98
S191-(+)ssRNA-21	9370	12	118	30	0	0	0	160	1.71
S192-(+)ssRNA-22	9263	54	142	9	0	0	0	205	2.21
S193-(+)ssRNA-23	11443	52	182	27	24	0	0	285	2.49
S194-(+)ssRNA-24	9704	7	196	18	0	0	18	239	2.46
S195-(+)ssRNA-25	9535	6	126	27	0	0	0	159	1.67
S196-(+)ssRNA-26	11219	25	206	72	0	40	0	343	3.06
S197-(+)ssRNA-27	10818	37	96	45	0	0	0	178	1.65
S198-(+)ssRNA-28	9384	6	96	18	0	0	0	120	1.28
S199-(+)ssRNA-29	8284	36	74	9	0	0	0	119	1.44
S200-(+)ssRNA-30	7437	6	90	9	0	0	0	105	1.41
S201-(+)ssRNA-31	7654	50	92	45	0	0	0	187	2.44
S202-(+)ssRNA-32	7476	18	104	18	12	0	0	152	2.03
S203-(+)ssRNA-33	7176	32	68	57	0	0	0	157	2.19
S204-(+)ssRNA-34	6813	57	82	27	0	0	0	166	2.44
S205-(+)ssRNA-35	7003	70	116	36	0	0	0	222	3.17
S206-(+)ssRNA-36	4540	0	18	9	0	0	0	27	0.59
S207-(+)ssRNA-37	4528	8	30	18	0	0	0	56	1.24
S208-(+)ssRNA-38	6625	30	60	51	0	0	0	141	2.13
S209-(+)ssRNA-39	4194	12	68	18	0	0	0	98	2.34
S210-(+)ssRNA-40	5677	32	78	27	12	0	0	149	2.62
S211-(+)ssRNA-41	5987	6	64	27	0	0	0	97	1.62

S212-(+)ssRNA-42	5706	33	32	18	0	0	0	83	1.45
S213-(+)ssRNA-43	4776	12	94	9	0	0	0	115	2.41
S214-(+)ssRNA-44	4003	27	74	0	0	0	0	101	2.52
S215-(+)ssRNA-45	3684	26	30	42	0	0	0	98	2.66
S216-(+)ssRNA-46	5243	6	54	0	0	0	0	60	1.14
S217-(+)ssRNA-47	4437	6	86	9	0	0	0	113	2.55
S218-(+)ssRNA-48	4114	18	62	0	0	0	0	68	1.65
S219-(+)ssRNA-49	4354	24	62	27	0	0	0	113	2.60
S220-(+)ssRNA-50	4326	27	54	27	0	0	0	108	2.50
S221-(+)ssRNA-51	12704	7	174	99	0	0	0	280	2.20
S222-(+)ssRNA-52	27608	61	410	126	0	0	0	597	2.16
S223-(+)ssRNA-53	28475	249	308	126	0	0	0	683	2.40
S224-(+)ssRNA-54	26253	36	586	132	0	0	0	754	2.87
S225-(+)ssRNA-55	10862	60	180	45	0	0	0	285	2.62
S226-(+)ssRNA-56	12573	115	194	93	0	0	0	402	3.20
S227-(+)ssRNA-57	9646	199	98	36	0	0	0	333	3.45
S228-(+)ssRNA-58	11703	49	102	45	12	0	0	208	1.78
S229-(+)ssRNA-59	9755	97	298	90	0	0	0	485	4.97
S230-(+)ssRNA-60	6395	48	130	30	0	0	0	208	3.25
S231-(+)ssRNA-61	10646	54	156	66	0	0	0	276	2.59
S232-(+)ssRNA-62	10221	81	96	63	0	0	0	240	2.35
S233-(+)ssRNA-63	10692	63	150	96	0	0	0	309	2.89
S234-(+)ssRNA-64	12141	142	140	87	0	0	0	369	3.04
S235-(+)ssRNA-65	10401	67	204	102	0	0	0	373	3.59
S236-(+)ssRNA-66	15914	24	206	162	0	0	18	410	2.58
S237-(+)ssRNA-67	8274	39	98	18	0	0	0	155	1.87
S238-(+)ssRNA-68	8622	24	146	18	0	0	0	188	2.18
S239-(+)ssRNA-69	8210	28	148	57	0	0	0	233	2.84
S240-(+)ssRNA-70	8623	25	142	45	0	0	0	212	2.46
S241-(+)ssRNA-71	8301	74	80	27	0	0	0	181	2.18
S242-(+)ssRNA-72	4852	19	30	30	0	0	0	79	1.63
S243-(+)ssRNA-73	7680	19	130	18	0	0	0	167	2.17
S244-(+)ssRNA-74	7564	248	60	105	0	0	0	413	5.46
S245-(+)ssRNA-75	6318	56	60	9	0	0	0	125	1.98
S246-(+)ssRNA-76	6305	25	72	27	0	0	0	124	1.97
S247-(+)ssRNA-77	15480	72	200	57	0	0	0	329	2.13
S248-(+)ssRNA-78	15311	67	220	66	12	0	0	365	2.38
S249-(+)ssRNA-79	17919	96	208	72	0	0	0	376	2.10
S250-(+)ssRNA-80	6435	6	78	36	0	0	0	120	1.86
S251-(+)ssRNA-81	7560	7	174	54	12	0	0	247	3.27
S252-(+)ssRNA-82	8832	14	126	0	0	0	0	140	1.59
S253-(+)ssRNA-83	9306	31	124	36	0	0	0	191	2.05
S254-(+)ssRNA-84	6495	69	60	9	12	0	0	150	2.31

S255-(+)ssRNA-85	7351	6	88	18	0	0	0	112	1.52
S256-(+)ssRNA-86	7555	59	114	27	0	0	0	200	2.65
S257-(+)ssRNA-87	4009	13	24	27	0	0	0	64	1.60
