# Supplementary material for: Coevolution between simple sequence repeats (SSRs) and virus genome size
Source: BMC Genomics. 2012 Aug 30;13:435. doi: 10.1186/1471-2164-13-435 (PMC3585866; doi:10.1186/1471-2164-13-435)
Supplement: Additional file 4 — Relative abundance of SSRs in analyzed virus genomes. [file 1471-2164-13-435-S4.pdf]

Additional file 4 Relative abundance of SSRs in analyzed virus genomes

No. Type	Genome size (bp)	Mono-	Di-	Tri-	Tetra-	Penta-	Hexa-	Total
S1-dsDNA-1	168903	1.13	1.25	0.40	0.0059	0.0059	0.0059	2.79
S2-dsDNA-2	94800	1.47	1.36	0.35	0	0	0	3.18
S3-dsDNA-3	33593	2.17	1.82	0.45	0.0298	0	0	4.47
S4-dsDNA-4	36717	1.50	0.98	0.22	0	0	0	2.70
S5-dsDNA-5	132562	1.12	1.92	0.28	0	0	0	3.33
S6-dsDNA-6	48502	1.71	1.48	0.39	0	0	0	3.59
S7-dsDNA-7	48836	1.35	1.29	0.33	0.0205	0	0	2.99
S8-dsDNA-8	121750	0.82	1.67	0.38	0.0082	0	0	2.87
S9-dsDNA-9	22172	2.89	1.67	0.68	0.0451	0	0	5.28
S10-dsDNA-10	52297	0.25	0.88	0.52	0.0191	0	0	1.66
S11-dsDNA-11	46375	1.55	1.27	0.39	0	0.0216	0	3.23
S12-dsDNA-12	41491	0.27	2.46	0.48	0	0	0	3.21
S13-dsDNA-13	39937	0.03	1.55	0.30	0	0	0	1.88
S14-dsDNA-14	19282	0.26	1.82	0.31	0	0	0	2.39
S15-dsDNA-15	41724	1.10	1.27	0.50	0	0	0	2.88
S16-dsDNA-16	70153	0.36	0.87	0.54	0.0285	0	0	1.80
S17-dsDNA-17	14927	1.00	0.27	0.54	0	0	0	1.81
S18-dsDNA-18	10079	3.57	3.08	0.79	0	0	0	7.44
S19-dsDNA-19	11965	1.76	1.50	0.25	0	0	0	3.51
S20-dsDNA-20	40900	3.13	2.64	0.44	0	0	0	6.21
S21-dsDNA-21	20869	2.11	3.07	0.34	0.0479	0	0	5.56
S22-dsDNA-22	35450	5.44	3.61	0.54	0	0.0564	0	9.65
S23-dsDNA-23	15465	1.29	2.33	0.19	0	0	0	3.82
S24-dsDNA-24	14462	0.35	2.01	0.00	0.0691	0	0	2.42
S25-dsDNA-25	194711	2.54	3.47	0.55	0.0257	0.0051	0.0103	6.61
S26-dsDNA-26	139962	0.36	6.13	1.24	0.1429	0	0.0143	7.88
S27-dsDNA-27	288539	2.51	3.42	0.59	0.0347	0.0035	0	6.56
S28-dsDNA-28	149955	7.38	3.28	0.77	0.0533	0.0067	0.0067	11.50
S29-dsDNA-29	161773	1.82	2.37	0.37	0.0247	0	0	4.59
S30-dsDNA-30	146454	3.66	5.13	0.82	0.0546	0.0068	0.0205	9.70
S31-dsDNA-31	190289	0.65	7.09	0.83	0.0158	0.0105	0.0158	8.60
S32-dsDNA-32	134721	6.21	2.50	0.49	0.0148	0	0	9.21
S33-dsDNA-33	232392	5.95	7.98	2.38	0.0947	0.0258	0.0129	16.45
S34-dsDNA-34	170101	4.63	1.80	0.47	0.0353	0	0	6.94
S35-dsDNA-35	212482	4.96	1.96	0.88	0.0329	0.0094	0.0047	7.85
S36-dsDNA-36	191100	1.50	1.15	0.64	0.0314	0.0052	0.0052	3.33
S37-dsDNA-37	105903	0.85	2.45	0.60	0	0	0	3.90
S38-dsDNA-38	102653	3.95	2.00	0.60	0.0097	0.0195	0.0195	6.60
S39-dsDNA-39	111362	0.57	3.62	0.70	0.0090	0	0	4.90

S40-dsDNA-40	330743	2.49	2.26	0.43	0.0060	0.0121	0.0060	5.19
S41-dsDNA-41	335593	1.46	2.98	0.91	0.0328	0.0030	0.0566	5.44
S42-dsDNA-42	407339	1.89	2.45	0.95	0.0319	0	0.0074	5.33
S43-dsDNA-43	133894	2.29	2.31	0.75	0.0299	0.0149	0.0224	5.41
S44-dsDNA-44	123500	1.67	3.53	1.20	0.0729	0.0081	0.0081	6.49
S45-dsDNA-45	305107	1.71	2.10	1.82	0.0164	0	0.0262	5.67
S46-dsDNA-46	134226	2.10	3.05	0.58	0.0298	0.0224	0.0149	5.80
S47-dsDNA-47	152261	4.35	3.07	1.06	0.1248	0.0263	0.0263	8.66
S48-dsDNA-48	124884	2.27	2.52	0.31	0.0400	0.0160	0.0080	5.16
S49-dsDNA-49	177874	1.96	2.48	0.31	0.0281	0.0112	0.1462	4.94
S50-dsDNA-50	148687	1.65	2.46	0.40	0.0202	0	0	4.53
S51-dsDNA-51	235646	1.46	2.91	1.26	0.0085	0	0	5.63
S52-dsDNA-52	230278	1.04	2.95	1.54	0.0782	0	0.0217	5.63
S53-dsDNA-53	159322	3.12	2.70	0.36	0.0063	0	0.1695	6.36
S54-dsDNA-54	172764	2.43	1.93	0.76	0.0289	0	0	5.16
S55-dsDNA-55	112930	3.13	2.74	0.59	0.0177	0	0	6.48
S56-dsDNA-56	35937	1.89	2.64	1.17	0	0	0.0278	5.73
S57-dsDNA-57	43804	1.07	2.47	0.82	0	0	0	4.36
S58-dsDNA-58	29576	3.79	1.52	0.37	0	0	0	5.68
S59-dsDNA-59	26163	2.79	1.72	0.42	0	0	0	4.93
S60-dsDNA-60	5243	4.01	1.14	0.76	0	0	0	5.91
S61-dsDNA-61	7961	1.88	4.02	0.88	0	0	0	6.78
S62-dsDNA-62	7746	2.07	2.32	0.39	0	0	0	4.78
S63-dsDNA-63	7353	1.22	2.04	0.82	0	0	0	4.08
S64-dsDNA-64	8095	1.61	1.85	0.12	0	0	0	3.58
S65-dsDNA-65	7841	2.55	1.91	0.77	0	0	0	5.23
S66-dsDNA-66	7610	3.55	1.84	0.66	0	0	0	6.04
S67-dsDNA-67	7729	1.04	1.04	0.52	0	0	0	2.59
S68-dsDNA-68	7304	0.82	2.19	0.41	0	0	0	3.42
S69-dsDNA-69	7687	1.30	1.95	0.52	0	0	0	3.77
S70-dsDNA-70	7868	2.16	1.27	0.51	0	0	0	3.94
S71-dsDNA-71	8607	3.60	2.67	0.58	0.1162	0	0	6.97
S72-dsDNA-72	7815	1.41	2.30	0.51	0	0	0	4.22
S73-dsDNA-73	7614	1.44	1.84	0.92	0	0	0	4.20
S74-dsDNA-74	7276	1.51	1.92	0.96	0	0	0	4.40
S75-dsDNA-75	7879	1.40	3.17	0.76	0	0	0	5.33
S76-dsDNA-76	246734	1.90	2.01	0.46	0.0689	0.0122	0.0041	4.45
S77-dsDNA-77	156922	0.50	2.26	0.77	0.0382	0	0	3.56
S78-ssDNA-1	6407	0.94	1.09	0.78	0	0	0	2.81
S79-ssDNA-2	4491	3.56	3.56	1.11	0	0	0	8.24
S80-ssDNA-3	5386	0.74	0.74	0.00	0	0	0	1.49
S81-ssDNA-4	4421	3.39	1.81	0.23	0	0	0	5.43
S82-ssDNA-5	4594	3.27	1.52	0.87	0	0	0	5.66

S83-ssDNA-6	4877	1.23	0.82	1.03	0	0	0	3.08
S84-ssDNA-7	2690	1.12	1.86	0.74	0	0	0	3.72
S85-ssDNA-8	2994	1.34	2.00	1.67	0	0	0	5.01
S86-ssDNA-9	5232	0.76	2.10	0.38	0	0	0	3.25
S87-ssDNA-10	2861	1.75	2.45	1.05	0	0	0	5.24
S88-ssDNA-11	1758	2.28	1.14	1.14	0.5688	0	0	5.12
S89-ssDNA-12	2319	7.33	3.02	0.43	0	0	0	10.78
S90-ssDNA-13	3852	5.71	3.37	1.56	0	0	0	10.64
S91-ssDNA-14	8024	0.25	3.49	1.25	0	0.1246	0	5.11
S92-ssDNA-15	6396	1.09	2.97	1.41	0	0	0	5.47
S93-ssDNA-16	5149	2.33	2.33	0.58	0	0	0	5.24
S94-ssDNA-17	5594	2.86	2.50	0.36	0	0	0	5.72
S95-ssDNA-18	4679	0.43	3.42	1.07	0.8549	0	0	5.77
S96-ssDNA-19	4801	1.46	3.54	1.25	0	0	0	6.25
S97-ssDNA-20	5517	2.54	5.08	0.91	0	0	0	8.52
S98-ssDNA-21	5908	1.18	1.86	1.35	0	0	0	4.40
S99-ssDNA-22	5078	3.15	3.54	1.58	0.1969	0	0	8.47
S100-ssDNA-23	3776	2.38	2.91	1.06	0	0	0	6.36
S101-ssDNA-24	5454	1.10	1.65	1.28	0	0	0	4.03
S102-dsDNA-RT-1	3215	0.62	2.18	0.62	0	0	0	3.42
S103-dsDNA-RT-2	3027	0.00	1.65	1.32	0	0	0	2.97
S104-dsDNA-RT-3	8024	1.00	1.87	0.87	0	0	0	3.74
S105-dsDNA-RT-4	8178	3.91	1.47	0.49	0	0	0	5.87
S106-dsDNA-RT-5	8159	4.41	3.31	1.35	0.1226	0	0	9.19
S107-dsDNA-RT-6	8002	2.00	2.62	0.50	0	0	0	5.12
S108-dsDNA-RT-7	7489	0.13	1.34	0.93	0	0	0	2.40
S109-dsDNA-RT-8	7206	1.80	1.39	0.56	0	0	0	3.75
S110-ssRNA-RT-1	8805	1.36	1.82	0.34	0.1136	0	0	3.63
S111-ssRNA-RT-2	8282	1.33	1.93	0.24	0	0	0	3.50
S112-ssRNA-RT-3	7286	0.69	3.16	0.41	0	0	0	4.25
S113-ssRNA-RT-4	8419	2.61	3.09	0.24	0	0	0	5.94
S114-ssRNA-RT-5	9181	2.29	3.49	1.31	0	0	0	7.08
S115-ssRNA-RT-6	12708	1.57	1.73	0.55	0.0787	0	0	3.93
S116-ssRNA-RT-7	13246	0.91	2.26	0.75	0	0	0.0755	4.00
S117-dsRNA-1	13385	0.30	2.17	0.37	0	0	0	2.84
S118-dsRNA-2	23564	0.25	1.99	0.34	0	0	0	2.59
S119-dsRNA-3	19208	1.09	2.34	0.57	0	0	0	4.01
S120-dsRNA-4	17448	1.55	2.24	0.46	0	0	0	4.24
S121-dsRNA-5	29174	0.55	2.16	0.55	0.0343	0	0	3.29
S122-dsRNA-6	23015	0.65	1.87	0.39	0	0	0	2.91
S123-dsRNA-7	24732	0.04	2.59	0.28	0	0	0	2.91
S124-dsRNA-8	29339	1.87	1.77	0.61	0	0	0	4.26
S125-dsRNA-9	25709	0.62	1.83	0.51	0	0	0	2.96

S126-dsRNA-10	26164	0.15	2.33	0.34	0	0	0	2.83
S127-dsRNA-11	20682	0.24	1.45	0.48	0	0	0	2.18
S128-dsRNA-12	23433	0.17	1.96	0.34	0.0427	0	0	2.52
S129-dsRNA-13	5881	0.85	2.21	0.51	0	0	0	3.57
S130-dsRNA-14	5898	0.17	1.70	0.34	0	0	0	2.20
S131-dsRNA-15	6603	1.06	1.51	0.15	0	0	0	2.73
S132-dsRNA-16	4579	0.22	1.97	0.22	0	0	0	2.40
S133-dsRNA-17	6277	1.12	1.91	0.32	0	0	0	3.35
S134-dsRNA-18	5284	1.14	2.65	0.57	0	0	0	4.35
S135-dsRNA-19	6105	1.64	1.97	0.49	0	0	0	4.10
S136-dsRNA-20	3663	0.55	2.18	0.27	0	0	0	3.00
S137-dsRNA-21	12640	1.34	1.82	1.19	0	0	0	4.35
S138-dsRNA-22	12734	0.16	1.41	0.39	0	0	0	1.96
S139-dsRNA-23	17635	1.30	2.32	0.51	0	0	0	4.14
S140-(-)ssRNA-1	8910	1.12	1.91	0.22	0	0	0	3.25
S141-(-)ssRNA-2	11161	1.70	2.24	0.27	0	0	0	4.21
S142-(-)ssRNA-3	11932	1.76	2.85	0.17	0	0	0	4.78
S143-(-)ssRNA-4	14900	2.75	2.48	0.54	0.0671	0	0	5.84
S144-(-)ssRNA-5	12807	0.70	2.97	0.62	0	0	0	4.29
S145-(-)ssRNA-6	12020	0.50	2.33	0.75	0	0	0	3.58
S146-(-)ssRNA-7	11131	1.80	1.98	0.99	0	0	0	4.76
S147-(-)ssRNA-8	19111	1.20	1.94	0.16	0	0	0	3.30
S148-(-)ssRNA-9	18959	1.37	2.00	0.32	0.1055	0	0	3.80
S149-(-)ssRNA-10	15384	0.33	2.47	0.26	0	0	0	3.06
S150-(-)ssRNA-11	15894	1.51	1.70	0.25	0	0	0	3.46
S151-(-)ssRNA-12	15384	1.30	2.54	0.07	0	0	0	3.90
S152-(-)ssRNA-13	18234	0.82	2.41	0.49	0	0	0	3.73
S153-(-)ssRNA-14	15186	1.45	2.44	0.26	0	0	0	4.15
S154-(-)ssRNA-15	15225	2.43	2.50	0.33	0	0	0	5.25
S155-(-)ssRNA-16	14071	1.92	3.13	0.71	0	0	0	5.76
S156-(-)ssRNA-17	12878	1.32	3.80	0.62	0	0	0	5.75
S157-(-)ssRNA-18	11278	2.75	2.84	0.80	0	0	0	6.38
S158-(-)ssRNA-19	13460	0.59	2.90	0.45	0	0	0	3.94
S159-(-)ssRNA-20	12555	1.59	2.39	0.48	0	0	0	4.46
S160-(-)ssRNA-21	10461	0.86	3.54	0.38	0	0	0	4.78
S161-(-)ssRNA-22	14452	1.04	1.52	0.62	0.0692	0	0	3.25
S162-(-)ssRNA-23	12716	0.55	2.67	0.79	0.0786	0	0	4.09
S163-(-)ssRNA-24	12294	1.38	3.01	0.89	0.0813	0	0	5.37
S164-(-)ssRNA-25	11845	1.60	2.87	1.27	0.0844	0	0	5.83
S165-(-)ssRNA-26	18859	2.12	2.01	0.48	0	0	0	4.61
S166-(-)ssRNA-27	11979	0.67	3.01	1.09	0	0	0	4.76
S167-(-)ssRNA-28	16634	3.55	2.22	0.48	0	0.1202	0	6.37
S168-(-)ssRNA-29	17145	1.63	3.62	0.35	0	0	0	5.60

S169-(-)ssRNA-30	10056	0.99	1.79	0.10	0	0	0	2.88
S170-(-)ssRNA-31	1682	3.57	2.97	0.59	0	0	0	7.13
S171-(+)ssRNA-1	3569	0.56	1.68	0.00	0	0	0	2.24
S172-(+)ssRNA-2	4215	0.47	0.95	0.47	0	0	0	1.90
S173-(+)ssRNA-3	2514	1.19	1.19	0.40	0	0	0	2.78
S174-(+)ssRNA-4	2728	0.00	2.57	0.00	0	0	0	2.57
S175-(+)ssRNA-5	7440	0.40	2.02	0.40	0	0	0	2.82
S176-(+)ssRNA-6	7152	0.28	3.22	0.14	0	0	0	3.64
S177-(+)ssRNA-7	7478	1.20	1.20	0.53	0	0	0	2.94
S178-(+)ssRNA-8	7835	1.02	2.30	0.51	0	0	0	3.83
S179-(+)ssRNA-9	8161	0.25	2.82	0.37	0	0	0	3.43
S180-(+)ssRNA-10	7348	0.82	1.63	0.00	0	0	0	2.45
S181-(+)ssRNA-11	8828	0.34	1.81	0.11	0	0	0	2.27
S182-(+)ssRNA-12	8251	1.94	2.79	0.24	0.3636	0	0	5.33
S183-(+)ssRNA-13	7117	0.28	2.25	0.28	0	0	0	2.81
S184-(+)ssRNA-14	9650	1.14	2.59	0.31	0	0	0	4.04
S185-(+)ssRNA-15	9185	0.44	2.83	0.65	0	0	0	3.92
S186-(+)ssRNA-16	8587	0.70	1.86	0.47	0	0	0	3.03
S187-(+)ssRNA-17	9871	0.41	2.84	0.30	0	0	0	3.55
S188-(+)ssRNA-18	12226	0.25	2.62	0.08	0	0	0	2.94
S189-(+)ssRNA-19	12138	0.91	3.21	0.58	0	0	0	4.70
S190-(+)ssRNA-20	10349	1.16	2.80	0.48	0	0	0	4.44
S191-(+)ssRNA-21	9370	0.21	1.92	0.32	0	0	0	2.45
S192-(+)ssRNA-22	9263	0.97	2.48	0.11	0	0	0	3.56
S193-(+)ssRNA-23	11443	0.70	2.62	0.26	0.1748	0	0	3.76
S194-(+)ssRNA-24	9704	0.10	3.30	0.21	0	0	0.1031	3.71
S195-(+)ssRNA-25	9535	0.10	2.20	0.31	0	0	0	2.62
S196-(+)ssRNA-26	11219	0.36	3.03	0.71	0	0.1783	0	4.28
S197-(+)ssRNA-27	10818	0.55	1.48	0.46	0	0	0	2.50
S198-(+)ssRNA-28	9384	0.11	1.71	0.21	0	0	0	2.02
S199-(+)ssRNA-29	8284	0.60	1.45	0.12	0	0	0	2.17
S200-(+)ssRNA-30	7437	0.13	2.02	0.13	0	0	0	2.29
S201-(+)ssRNA-31	7654	1.05	1.96	0.65	0	0	0	3.66
S202-(+)ssRNA-32	7476	0.40	2.27	0.27	0.1338	0	0	3.08
S203-(+)ssRNA-33	7176	0.70	1.53	0.84	0	0	0	3.07
S204-(+)ssRNA-34	6813	1.32	1.91	0.44	0	0	0	3.67
S205-(+)ssRNA-35	7003	1.57	2.71	0.57	0	0	0	4.86
S206-(+)ssRNA-36	4540	0.00	0.66	0.22	0	0	0	0.88
S207-(+)ssRNA-37	4528	0.22	1.10	0.44	0	0	0	1.77
S208-(+)ssRNA-38	6625	0.75	1.51	0.75	0	0	0	3.02
S209-(+)ssRNA-39	4194	0.48	2.62	0.48	0	0	0	3.58
S210-(+)ssRNA-40	5677	0.88	2.29	0.53	0.1761	0	0	3.88
S211-(+)ssRNA-41	5987	0.17	1.67	0.50	0	0	0	2.34

S212-(+)ssRNA-42	5706	0.88	0.88	0.35	0	0	0	2.10
S213-(+)ssRNA-43	4776	0.42	3.14	0.21	0	0	0	3.77
S214-(+)ssRNA-44	4003	1.00	3.00	0.00	0	0	0	4.00
S215-(+)ssRNA-45	3684	1.09	1.36	1.09	0	0	0	3.53
S216-(+)ssRNA-46	5243	0.19	1.72	0.00	0	0	0	1.91
S217-(+)ssRNA-47	4437	0.45	2.93	0.23	0	0	0	3.61
S218-(+)ssRNA-48	4114	0.24	2.43	0.00	0	0	0	2.67
S219-(+)ssRNA-49	4354	0.92	2.30	0.69	0	0	0	3.90
S220-(+)ssRNA-50	4326	0.92	2.08	0.69	0	0	0	3.70
S221-(+)ssRNA-51	12704	0.08	2.20	0.87	0	0	0	3.15
S222-(+)ssRNA-52	27608	0.36	2.39	0.51	0	0	0	3.26
S223-(+)ssRNA-53	28475	1.37	1.72	0.49	0	0	0	3.58
S224-(+)ssRNA-54	26253	0.15	3.54	0.53	0	0	0	4.23
S225-(+)ssRNA-55	10862	0.92	2.67	0.46	0	0	0	4.05
S226-(+)ssRNA-56	12573	1.51	2.47	0.80	0	0	0	4.77
S227-(+)ssRNA-57	9646	2.07	1.66	0.41	0	0	0	4.15
S228-(+)ssRNA-58	11703	0.68	1.45	0.43	0.0854	0	0	2.65
S229-(+)ssRNA-59	9755	1.54	4.82	0.92	0	0	0	7.28
S230-(+)ssRNA-60	6395	1.25	3.28	0.47	0	0	0	5.00
S231-(+)ssRNA-61	10646	0.85	2.35	0.66	0	0	0	3.85
S232-(+)ssRNA-62	10221	0.59	1.47	0.68	0	0	0	2.74
S233-(+)ssRNA-63	10692	0.94	2.15	0.94	0	0	0	4.02
S234-(+)ssRNA-64	12141	1.81	1.89	0.74	0	0	0	4.45
S235-(+)ssRNA-65	10401	1.06	3.17	1.06	0	0	0	5.29
S236-(+)ssRNA-66	15914	0.25	2.07	1.07	0	0	0.0628	3.46
S237-(+)ssRNA-67	8274	0.73	1.93	0.24	0	0	0	2.90
S238-(+)ssRNA-68	8622	0.46	2.78	0.23	0	0	0	3.48
S239-(+)ssRNA-69	8210	0.37	2.92	0.73	0	0	0	4.02
S240-(+)ssRNA-70	8623	0.46	2.55	0.58	0	0	0	3.60
S241-(+)ssRNA-71	8301	1.20	1.57	0.36	0	0	0	3.13
S242-(+)ssRNA-72	4852	0.62	1.03	0.62	0	0	0	2.27
S243-(+)ssRNA-73	7680	0.39	2.73	0.26	0	0	0	3.39
S244-(+)ssRNA-74	7564	4.36	1.32	1.45	0	0	0	7.14
S245-(+)ssRNA-75	6318	1.27	1.58	0.16	0	0	0	3.01
S246-(+)ssRNA-76	6305	0.63	1.90	0.48	0	0	0	3.01
S247-(+)ssRNA-77	15480	0.78	2.07	0.39	0	0	0	3.23
S248-(+)ssRNA-78	15311	0.72	2.35	0.46	0.0653	0	0	3.59
S249-(+)ssRNA-79	17919	0.89	1.90	0.45	0	0	0	3.24
S250-(+)ssRNA-80	6435	0.16	2.02	0.62	0	0	0	2.80
S251-(+)ssRNA-81	7560	0.13	3.84	0.79	0.1323	0	0	4.89
S252-(+)ssRNA-82	8832	0.23	2.26	0.00	0	0	0	2.49
S253-(+)ssRNA-83	9306	0.54	2.15	0.43	0	0	0	3.12
S254-(+)ssRNA-84	6495	1.69	1.54	0.15	0.1540	0	0	3.54

S255-(+)ssRNA-85	7351	0.14	1.90	0.27	0	0	0	2.31
S256-(+)ssRNA-86	7555	1.19	2.51	0.40	0	0	0	4.10
S257-(+)ssRNA-87	4009	0.50	1.00	0.75	0	0	0	2.24
