# Supplementary material for: Coevolution between simple sequence repeats (SSRs) and virus genome size
Source: BMC Genomics. 2012 Aug 30;13:435. doi: 10.1186/1471-2164-13-435 (PMC3585866; doi:10.1186/1471-2164-13-435)
Supplement: Additional file 5 — Relative density of SSRs in analyzed virus genomes. [file 1471-2164-13-435-S5.pdf]

Additional file 5 Relative density of SSRs in analyzed virus genomes

No. Type	Genome size (bp)	Mono-	Di-	Tri-	Tetra-	Penta-	Hexa-	Total
S1-dsDNA-1	168903	6.83	7.52	3.57	0.071	0.089	0.107	18.19
S2-dsDNA-2	94800	9.19	8.40	3.16	0	0	0	20.75
S3-dsDNA-3	33593	13.66	11.01	4.02	0.357	0	0	29.05
S4-dsDNA-4	36717	9.21	6.05	1.96	0	0	0	17.21
S5-dsDNA-5	132562	7.37	11.83	2.51	0	0	0	21.71
S6-dsDNA-6	48502	10.66	9.07	3.59	0	0	0	23.32
S7-dsDNA-7	48836	8.52	7.99	2.95	0.246	0	0	19.70
S8-dsDNA-8	121750	4.94	10.09	3.43	0.099	0	0	18.55
S9-dsDNA-9	22172	18.54	10.10	6.36	0.541	0	0	35.54
S10-dsDNA-10	52297	1.53	5.43	4.76	0.229	0	0	11.95
S11-dsDNA-11	46375	9.83	7.68	3.56	0	0.323	0	21.39
S12-dsDNA-12	41491	1.61	14.94	4.48	0	0	0	21.04
S13-dsDNA-13	39937	0.15	9.41	2.70	0	0	0	12.27
S14-dsDNA-14	19282	1.56	10.89	2.80	0	0	0	15.25
S15-dsDNA-15	41724	6.97	7.96	4.53	0	0	0	19.46
S16-dsDNA-16	70153	2.25	5.22	4.88	0.342	0	0	12.69
S17-dsDNA-17	14927	6.30	1.61	5.23	0	0	0	13.13
S18-dsDNA-18	10079	22.82	18.65	7.14	0	0	0	48.62
S19-dsDNA-19	11965	11.37	9.69	2.26	0	0	0	23.32
S20-dsDNA-20	40900	20.10	16.43	4.03	0	0	0	40.56
S21-dsDNA-21	20869	13.32	18.98	3.02	0.575	0	0	35.89
S22-dsDNA-22	35450	35.57	22.34	4.91	0	0.846	0	63.67
S23-dsDNA-23	15465	7.95	14.87	1.75	0	0	0	24.57
S24-dsDNA-24	14462	2.07	12.45	0.00	0.830	0	0	15.35
S25-dsDNA-25	194711	16.32	21.28	5.08	0.308	0.077	0.185	43.26
S26-dsDNA-26	139962	2.24	39.70	11.23	1.772	0	0.257	55.19
S27-dsDNA-27	288539	16.08	21.30	5.32	0.416	0.052	0	43.18
S28-dsDNA-28	149955	50.03	20.57	7.10	0.640	0.100	0.120	78.56
S29-dsDNA-29	161773	11.79	14.64	3.49	0.297	0	0	30.21
S30-dsDNA-30	146454	24.03	32.61	7.58	0.655	0.171	0.533	65.58
S31-dsDNA-31	190289	4.56	49.78	7.65	0.189	0.158	0.284	62.62
S32-dsDNA-32	134721	41.46	15.56	4.56	0.178	0	0	61.76
S33-dsDNA-33	232392	39.99	51.32	22.38	1.136	0.387	0.232	115.45
S34-dsDNA-34	170101	31.46	11.10	4.30	0.423	0	0	47.28
S35-dsDNA-35	212482	32.07	12.18	8.03	0.395	0.141	0.113	52.93
S36-dsDNA-36	191100	9.51	7.02	5.84	0.377	0.078	0.094	22.93
S37-dsDNA-37	105903	5.52	15.81	5.47	0	0	0	26.80
S38-dsDNA-38	102653	25.23	12.31	5.52	0.117	0.292	0.409	43.89
S39-dsDNA-39	111362	3.62	22.52	6.82	0.108	0	0	33.06

S40-dsDNA-40	330743	16.28	13.90	3.92	0.169	0.181	0.109	34.56
S41-dsDNA-41	335593	9.73	18.70	9.26	0.393	0.045	1.287	39.41
S42-dsDNA-42	407339	11.91	15.25	8.94	0.383	0	0.133	36.61
S43-dsDNA-43	133894	14.43	14.21	7.33	0.358	0.224	0.403	36.95
S44-dsDNA-44	123500	10.82	21.91	11.44	0.939	0.121	0.194	45.43
S45-dsDNA-45	305107	10.81	12.90	19.31	0.210	0	0.472	43.71
S46-dsDNA-46	134226	14.09	20.61	5.65	0.358	0.335	0.402	41.45
S47-dsDNA-47	152261	28.83	19.51	10.17	1.497	0.558	0.867	61.42
S48-dsDNA-48	124884	15.23	15.90	2.83	0.480	0.240	0.144	34.83
S49-dsDNA-49	177874	13.93	15.20	2.83	0.337	0.197	5.566	38.06
S50-dsDNA-50	148687	10.59	15.02	3.65	0.242	0	0	29.51
S51-dsDNA-51	235646	9.93	18.08	11.73	0.119	0	0	39.85
S52-dsDNA-52	230278	6.78	18.48	14.73	1.060	0	0.417	41.47
S53-dsDNA-53	159322	21.11	16.91	3.54	0.075	0	7.268	48.91
S54-dsDNA-54	172764	15.30	11.99	7.00	0.370	0	0	34.66
S55-dsDNA-55	112930	20.07	16.86	5.58	0.248	0	0	42.76
S56-dsDNA-56	35937	12.30	16.58	11.69	0	0	0.501	41.07
S57-dsDNA-57	43804	6.89	15.25	7.94	0	0	0	30.09
S58-dsDNA-58	29576	24.18	9.33	3.35	0	0	0	36.85
S59-dsDNA-59	26163	17.89	10.32	4.13	0	0	0	32.34
S60-dsDNA-60	5243	25.37	6.87	8.58	0	0	0	40.82
S61-dsDNA-61	7961	12.69	25.62	8.29	0	0	0	46.60
S62-dsDNA-62	7746	13.43	15.23	4.65	0	0	0	33.31
S63-dsDNA-63	7353	7.75	12.51	7.75	0	0	0	28.02
S64-dsDNA-64	8095	10.99	11.37	1.11	0	0	0	23.47
S65-dsDNA-65	7841	17.09	11.48	8.03	0	0	0	36.60
S66-dsDNA-66	7610	23.00	11.30	6.31	0	0	0	40.60
S67-dsDNA-67	7729	7.12	6.73	4.66	0	0	0	18.50
S68-dsDNA-68	7304	4.93	13.96	3.70	0	0	0	22.59
S69-dsDNA-69	7687	8.85	11.71	5.07	0	0	0	25.63
S70-dsDNA-70	7868	14.23	7.63	4.96	0	0	0	26.82
S71-dsDNA-71	8607	27.88	16.50	6.97	1.394	0	0	52.75
S72-dsDNA-72	7815	9.34	14.08	4.61	0	0	0	28.02
S73-dsDNA-73	7614	9.06	11.03	8.67	0	0	0	28.76
S74-dsDNA-74	7276	9.76	11.82	9.48	0	0	0	31.06
S75-dsDNA-75	7879	9.90	19.80	6.85	0	0	0	36.55
S76-dsDNA-76	246734	12.84	12.47	4.26	0.875	0.203	0.073	30.71
S77-dsDNA-77	156922	3.53	14.26	8.39	0.459	0	0	26.64
S78-ssDNA-1	6407	6.09	6.56	7.02	0	0	0	19.67
S79-ssDNA-2	4491	24.27	22.71	10.02	0	0	0	57.00
S80-ssDNA-3	5386	4.64	4.46	0.00	0	0	0	9.10
S81-ssDNA-4	4421	22.62	11.31	2.04	0	0	0	35.96
S82-ssDNA-5	4594	21.11	9.14	8.49	0	0	0	38.75

S83-ssDNA-6	4877	9.02	5.33	9.23	0	0	0	23.58
S84-ssDNA-7	2690	7.06	11.15	6.69	0	0	0	24.91
S85-ssDNA-8	2994	8.35	12.02	15.03	0	0	0	35.40
S86-ssDNA-9	5232	4.59	14.14	3.44	0	0	0	22.17
S87-ssDNA-10	2861	10.49	14.68	9.44	0	0	0	34.60
S88-ssDNA-11	1758	14.22	6.83	10.24	6.826	0	0	38.11
S89-ssDNA-12	2319	49.59	18.11	3.88	0	0	0	71.58
S90-ssDNA-13	3852	39.20	20.77	14.02	0	0	0	73.99
S91-ssDNA-14	8024	1.74	23.68	11.59	0	1.869	0	38.88
S92-ssDNA-15	6396	7.04	19.39	12.66	0	0	0	39.09
S93-ssDNA-16	5149	14.76	14.76	5.24	0	0	0	34.76
S94-ssDNA-17	5594	17.70	15.73	3.22	0	0	0	36.65
S95-ssDNA-18	4679	2.56	20.94	10.26	10.259	0	0	44.03
S96-ssDNA-19	4801	9.37	22.91	13.12	0	0	0	45.41
S97-ssDNA-20	5517	16.13	31.54	8.16	0	0	0	55.83
S98-ssDNA-21	5908	7.62	11.17	13.20	0	0	0	31.99
S99-ssDNA-22	5078	19.69	21.27	14.18	2.363	0	0	57.50
S100-ssDNA-23	3776	15.36	19.07	9.53	0	0	0	43.96
S101-ssDNA-24	5454	7.15	10.27	13.75	0	0	0	31.17
S102-dsDNA-RT-1	3215	3.73	13.06	5.60	0	0	0	22.40
S103-dsDNA-RT-2	3027	0.00	10.57	11.89	0	0	0	22.46
S104-dsDNA-RT-3	8024	6.11	11.96	7.85	0	0	0	25.92
S105-dsDNA-RT-4	8178	23.60	8.80	4.40	0	0	0	36.81
S106-dsDNA-RT-5	8159	26.84	21.33	12.50	1.471	0	0	62.14
S107-dsDNA-RT-6	8002	12.00	17.25	4.50	0	0	0	33.74
S108-dsDNA-RT-7	7489	0.80	8.01	8.41	0	0	0	17.23
S109-dsDNA-RT-8	7206	10.82	8.60	5.00	0	0	0	24.42
S110-ssRNA-RT-1	8805	8.18	11.36	3.41	1.363	0	0	24.30
S111-ssRNA-RT-2	8282	8.33	12.07	2.17	0	0	0	22.58
S112-ssRNA-RT-3	7286	4.12	19.49	4.12	0	0	0	27.72
S113-ssRNA-RT-4	8419	17.34	19.48	2.14	0	0	0	38.96
S114-ssRNA-RT-5	9181	13.83	22.00	12.09	0	0	0	47.93
S115-ssRNA-RT-6	12708	9.76	11.49	5.67	0.944	0	0	27.86
S116-ssRNA-RT-7	13246	5.44	13.74	6.79	0	0	1.359	27.33
S117-dsRNA-1	13385	2.02	14.05	3.36	0	0	0	19.42
S118-dsRNA-2	23564	1.61	12.39	3.06	0	0	0	17.06
S119-dsRNA-3	19208	6.72	14.68	5.15	0	0	0	26.55
S120-dsRNA-4	17448	9.46	14.10	4.13	0	0	0	27.68
S121-dsRNA-5	29174	3.36	13.44	4.94	0.411	0	0	22.14
S122-dsRNA-6	23015	4.17	11.21	3.52	0	0	0	18.90
S123-dsRNA-7	24732	0.24	16.01	2.55	0	0	0	18.80
S124-dsRNA-8	29339	11.45	10.98	5.52	0	0	0	27.95
S125-dsRNA-9	25709	3.81	11.28	4.90	0	0	0	19.99

S126-dsRNA-10	26164	0.96	14.45	3.21	0	0	0	18.61
S127-dsRNA-11	20682	1.45	9.09	4.50	0	0	0	15.04
S128-dsRNA-12	23433	1.19	12.03	3.07	0.512	0	0	16.81
S129-dsRNA-13	5881	5.27	13.26	4.59	0	0	0	23.13
S130-dsRNA-14	5898	1.02	10.17	3.05	0	0	0	14.24
S131-dsRNA-15	6603	6.66	9.09	1.36	0	0	0	17.11
S132-dsRNA-16	4579	1.53	11.79	1.97	0	0	0	15.29
S133-dsRNA-17	6277	7.65	12.11	2.87	0	0	0	22.62
S134-dsRNA-18	5284	6.81	16.28	5.11	0	0	0	28.20
S135-dsRNA-19	6105	12.78	12.45	4.42	0	0	0	29.65
S136-dsRNA-20	3663	3.82	13.10	3.28	0	0	0	20.20
S137-dsRNA-21	12640	8.62	11.39	12.10	0	0	0	32.12
S138-dsRNA-22	12734	2.28	8.48	3.53	0	0	0	14.29
S139-dsRNA-23	17635	8.56	14.74	4.59	0	0	0	27.90
S140-(-)ssRNA-1	8910	7.07	11.67	2.02	0	0	0	20.76
S141-(-)ssRNA-2	11161	10.66	13.62	2.69	0	0	0	26.97
S142-(-)ssRNA-3	11932	11.40	17.77	1.51	0	0	0	30.67
S143-(-)ssRNA-4	14900	17.92	15.97	5.03	0.805	0	0	39.73
S144-(-)ssRNA-5	12807	4.53	18.74	6.09	0	0	0	29.36
S145-(-)ssRNA-6	12020	3.00	14.98	7.24	0	0	0	25.21
S146-(-)ssRNA-7	11131	11.77	12.40	9.43	0	0	0	33.60
S147-(-)ssRNA-8	19111	7.27	11.83	1.41	0	0	0	20.51
S148-(-)ssRNA-9	18959	8.44	12.03	2.85	1.266	0	0	24.58
S149-(-)ssRNA-10	15384	1.95	15.34	2.34	0	0	0	19.63
S150-(-)ssRNA-11	15894	9.69	10.57	2.27	0	0	0	22.52
S151-(-)ssRNA-12	15384	8.06	15.47	0.59	0	0	0	24.12
S152-(-)ssRNA-13	18234	5.05	14.70	4.44	0	0	0	24.19
S153-(-)ssRNA-14	15186	8.89	15.15	2.37	0	0	0	26.41
S154-(-)ssRNA-15	15225	15.50	15.63	2.96	0	0	0	34.09
S155-(-)ssRNA-16	14071	11.94	19.33	6.61	0	0	0	37.88
S156-(-)ssRNA-17	12878	8.39	23.45	5.59	0	0	0	37.43
S157-(-)ssRNA-18	11278	17.73	17.73	7.98	0	0	0	43.45
S158-(-)ssRNA-19	13460	3.64	18.13	4.01	0	0	0	25.78
S159-(-)ssRNA-20	12555	9.56	14.81	4.30	0	0	0	28.67
S160-(-)ssRNA-21	10461	5.16	22.18	3.44	0	0	0	30.78
S161-(-)ssRNA-22	14452	6.30	9.27	5.60	0.830	0	0	22.00
S162-(-)ssRNA-23	12716	4.72	16.83	7.31	0.944	0	0	29.80
S163-(-)ssRNA-24	12294	8.70	19.03	8.05	0.976	0	0	36.77
S164-(-)ssRNA-25	11845	10.22	18.07	11.40	1.013	0	0	40.69
S165-(-)ssRNA-26	18859	13.63	12.20	4.30	0	0	0	30.12
S166-(-)ssRNA-27	11979	4.26	18.87	9.77	0	0	0	32.89
S167-(-)ssRNA-28	16634	22.06	13.59	4.51	0	1.804	0	41.96
S168-(-)ssRNA-29	17145	10.73	22.51	3.15	0	0	0	36.40

S169-(-)ssRNA-30	10056	6.07	10.94	0.89	0	0	0	17.90
S170-(-)ssRNA-31	1682	21.40	19.02	5.35	0	0	0	45.78
S171-(+)ssRNA-1	3569	3.36	10.09	0.00	0	0	0	13.45
S172-(+)ssRNA-2	4215	2.85	6.17	4.27	0	0	0	13.29
S173-(+)ssRNA-3	2514	7.16	8.75	3.58	0	0	0	19.49
S174-(+)ssRNA-4	2728	0.00	15.40	0.00	0	0	0	15.40
S175-(+)ssRNA-5	7440	2.69	12.37	3.63	0	0	0	18.68
S176-(+)ssRNA-6	7152	1.68	20.69	1.26	0	0	0	23.63
S177-(+)ssRNA-7	7478	7.22	7.22	4.81	0	0	0	19.26
S178-(+)ssRNA-8	7835	20.55	14.04	4.59	0	0	0	39.18
S179-(+)ssRNA-9	8161	3.31	17.40	3.31	0	0	0	24.02
S180-(+)ssRNA-10	7348	6.67	10.34	0.00	0	0	0	17.01
S181-(+)ssRNA-11	8828	2.04	11.33	1.02	0	0	0	14.39
S182-(+)ssRNA-12	8251	12.12	17.94	2.18	4.363	0	0	36.60
S183-(+)ssRNA-13	7117	2.81	13.49	2.53	0	0	0	18.83
S184-(+)ssRNA-14	9650	7.25	16.17	2.80	0	0	0	26.22
S185-(+)ssRNA-15	9185	2.72	17.20	6.53	0	0	0	26.46
S186-(+)ssRNA-16	8587	4.77	11.41	4.19	0	0	0	20.38
S187-(+)ssRNA-17	9871	2.63	17.83	3.04	0	0	0	23.50
S188-(+)ssRNA-18	12226	5.32	16.03	0.74	0	0	0	22.08
S189-(+)ssRNA-19	12138	5.44	20.27	5.19	0	0	0	30.89
S190-(+)ssRNA-20	10349	7.05	17.20	5.51	0	0	0	29.76
S191-(+)ssRNA-21	9370	1.28	12.59	3.20	0	0	0	17.08
S192-(+)ssRNA-22	9263	5.83	15.33	0.97	0	0	0	22.13
S193-(+)ssRNA-23	11443	4.54	15.90	2.36	2.097	0	0	24.91
S194-(+)ssRNA-24	9704	0.72	20.20	1.85	0	0	1.855	24.63
S195-(+)ssRNA-25	9535	0.63	13.21	2.83	0	0	0	16.68
S196-(+)ssRNA-26	11219	2.23	18.36	6.42	0	3.565	0	30.57
S197-(+)ssRNA-27	10818	3.42	8.87	4.16	0	0	0	16.45
S198-(+)ssRNA-28	9384	0.64	10.23	1.92	0	0	0	12.79
S199-(+)ssRNA-29	8284	4.35	8.93	1.09	0	0	0	14.37
S200-(+)ssRNA-30	7437	0.81	12.10	1.21	0	0	0	14.12
S201-(+)ssRNA-31	7654	6.53	12.02	5.88	0	0	0	24.43
S202-(+)ssRNA-32	7476	2.41	13.91	2.41	1.605	0	0	20.33
S203-(+)ssRNA-33	7176	4.46	9.48	7.94	0	0	0	21.88
S204-(+)ssRNA-34	6813	8.37	12.04	3.96	0	0	0	24.37
S205-(+)ssRNA-35	7003	10.00	16.56	5.14	0	0	0	31.70
S206-(+)ssRNA-36	4540	0.00	3.96	1.98	0	0	0	5.95
S207-(+)ssRNA-37	4528	1.77	6.63	3.98	0	0	0	12.37
S208-(+)ssRNA-38	6625	4.53	9.06	7.70	0	0	0	21.28
S209-(+)ssRNA-39	4194	2.86	16.21	4.29	0	0	0	23.37
S210-(+)ssRNA-40	5677	5.64	13.74	4.76	2.114	0	0	26.25
S211-(+)ssRNA-41	5987	1.00	10.69	4.51	0	0	0	16.20

S212-(+)ssRNA-42	5706	5.78	5.61	3.15	0	0	0	14.55
S213-(+)ssRNA-43	4776	2.51	19.68	1.88	0	0	0	24.08
S214-(+)ssRNA-44	4003	6.74	18.49	0.00	0	0	0	25.23
S215-(+)ssRNA-45	3684	7.06	8.14	11.40	0	0	0	26.60
S216-(+)ssRNA-46	5243	1.14	10.30	0.00	0	0	0	11.44
S217-(+)ssRNA-47	4437	1.35	19.38	2.03	0	0	0	25.47
S218-(+)ssRNA-48	4114	4.38	15.07	0.00	0	0	0	16.53
S219-(+)ssRNA-49	4354	5.51	14.24	6.20	0	0	0	25.95
S220-(+)ssRNA-50	4326	6.24	12.48	6.24	0	0	0	24.97
S221-(+)ssRNA-51	12704	0.55	13.70	7.79	0	0	0	22.04
S222-(+)ssRNA-52	27608	2.21	14.85	4.56	0	0	0	21.62
S223-(+)ssRNA-53	28475	8.74	10.82	4.42	0	0	0	23.99
S224-(+)ssRNA-54	26253	1.37	22.32	5.03	0	0	0	28.72
S225-(+)ssRNA-55	10862	5.52	16.57	4.14	0	0	0	26.24
S226-(+)ssRNA-56	12573	9.15	15.43	7.40	0	0	0	31.97
S227-(+)ssRNA-57	9646	20.63	10.16	3.73	0	0	0	34.52
S228-(+)ssRNA-58	11703	4.19	8.72	3.85	1.025	0	0	17.77
S229-(+)ssRNA-59	9755	9.94	30.55	9.23	0	0	0	49.72
S230-(+)ssRNA-60	6395	7.51	20.33	4.69	0	0	0	32.53
S231-(+)ssRNA-61	10646	5.07	14.65	6.20	0	0	0	25.93
S232-(+)ssRNA-62	10221	7.92	9.39	6.16	0	0	0	23.48
S233-(+)ssRNA-63	10692	5.89	14.03	8.98	0	0	0	28.90
S234-(+)ssRNA-64	12141	11.70	11.53	7.17	0	0	0	30.39
S235-(+)ssRNA-65	10401	6.44	19.61	9.81	0	0	0	35.86
S236-(+)ssRNA-66	15914	1.51	12.94	10.18	0	0	1.131	25.76
S237-(+)ssRNA-67	8274	4.71	11.84	2.18	0	0	0	18.73
S238-(+)ssRNA-68	8622	2.78	16.93	2.09	0	0	0	21.80
S239-(+)ssRNA-69	8210	3.41	18.03	6.94	0	0	0	28.38
S240-(+)ssRNA-70	8623	2.90	16.47	5.22	0	0	0	24.59
S241-(+)ssRNA-71	8301	8.91	9.64	3.25	0	0	0	21.80
S242-(+)ssRNA-72	4852	3.92	6.18	6.18	0	0	0	16.28
S243-(+)ssRNA-73	7680	2.47	16.93	2.34	0	0	0	21.74
S244-(+)ssRNA-74	7564	32.79	7.93	13.88	0	0	0	54.60
S245-(+)ssRNA-75	6318	8.86	9.50	1.42	0	0	0	19.78
S246-(+)ssRNA-76	6305	3.97	11.42	4.28	0	0	0	19.67
S247-(+)ssRNA-77	15480	4.65	12.92	3.68	0	0	0	21.25
S248-(+)ssRNA-78	15311	4.38	14.37	4.31	0.784	0	0	23.84
S249-(+)ssRNA-79	17919	5.36	11.61	4.02	0	0	0	20.98
S250-(+)ssRNA-80	6435	0.93	12.12	5.59	0	0	0	18.65
S251-(+)ssRNA-81	7560	0.93	23.02	7.14	1.587	0	0	32.67
S252-(+)ssRNA-82	8832	1.59	14.27	0.00	0	0	0	15.85
S253-(+)ssRNA-83	9306	3.33	13.32	3.87	0	0	0	20.52
S254-(+)ssRNA-84	6495	10.62	9.24	1.39	1.848	0	0	23.09

S255-(+)ssRNA-85	7351	0.82	11.97	2.45	0	0	0	15.24
S256-(+)ssRNA-86	7555	7.81	15.09	3.57	0	0	0	26.47
S257-(+)ssRNA-87	4009	3.24	5.99	6.73	0	0	0	15.96
