# Supplementary material for: Coevolution between simple sequence repeats (SSRs) and virus genome size
Source: BMC Genomics. 2012 Aug 30;13:435. doi: 10.1186/1471-2164-13-435 (PMC3585866; doi:10.1186/1471-2164-13-435)
Supplement: Additional file 6 — Scatter plots of SSRs relative abundance versus genome size. (A)Scatter plot of SSRs relative abundances in all analyzed virus genomes. (B) Scatter plot of SSRs relative abundances in analyzed virus genomes with size of < 30000 bp. (C) Scatter plot of SSRs relative abundances in analyzed virus genomes with size of > 30000 bp. [file 1471-2164-13-435-S6.pdf]

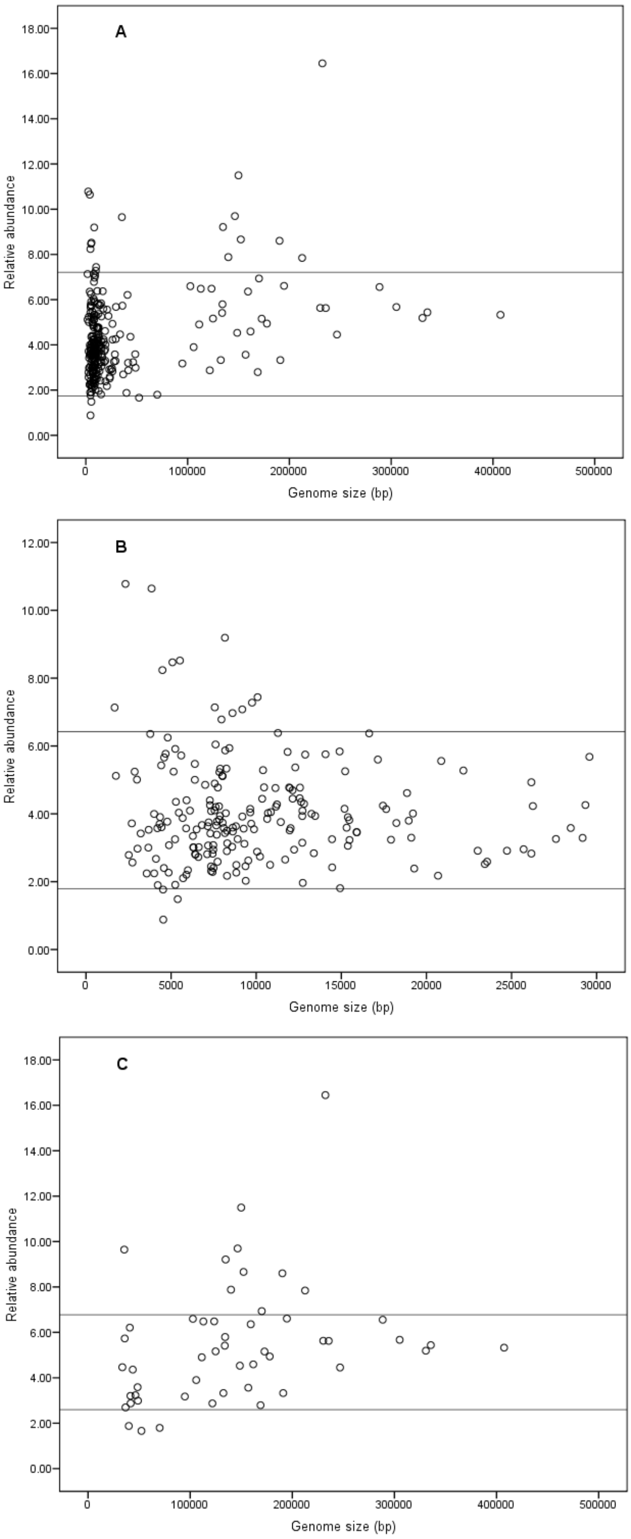

Additional file 13 Scatter plots of SSRs relative abundance versus genome size.

(*A*) Scatter plot of SSRs relative abundances in all analysed virus genomes. (*B*) Scatter plot of

SSRs relative abundances in analysed virus genomes with size of < 30000 bp. (*C*) Scatter plot of SSRs relative abundances in analysed virus genomes with size of > 30000 bp.
