# Supplementary material for: Coevolution between simple sequence repeats (SSRs) and virus genome size
Source: BMC Genomics. 2012 Aug 30;13:435. doi: 10.1186/1471-2164-13-435 (PMC3585866; doi:10.1186/1471-2164-13-435)
Supplement: Additional file 7 — Scatter plots of SSRs relative density versus genome size. (A) Scatter plot of SSRs relative densities in all analyzed virus genomes. (B) Scatter plot of SSRs relative densities in analyzed virus genomes with size of < 30000 bp. (C) Scatter plot of SSRs relative densities in analyzed virus genomes with size of > 30000 bp. [file 1471-2164-13-435-S7.pdf]

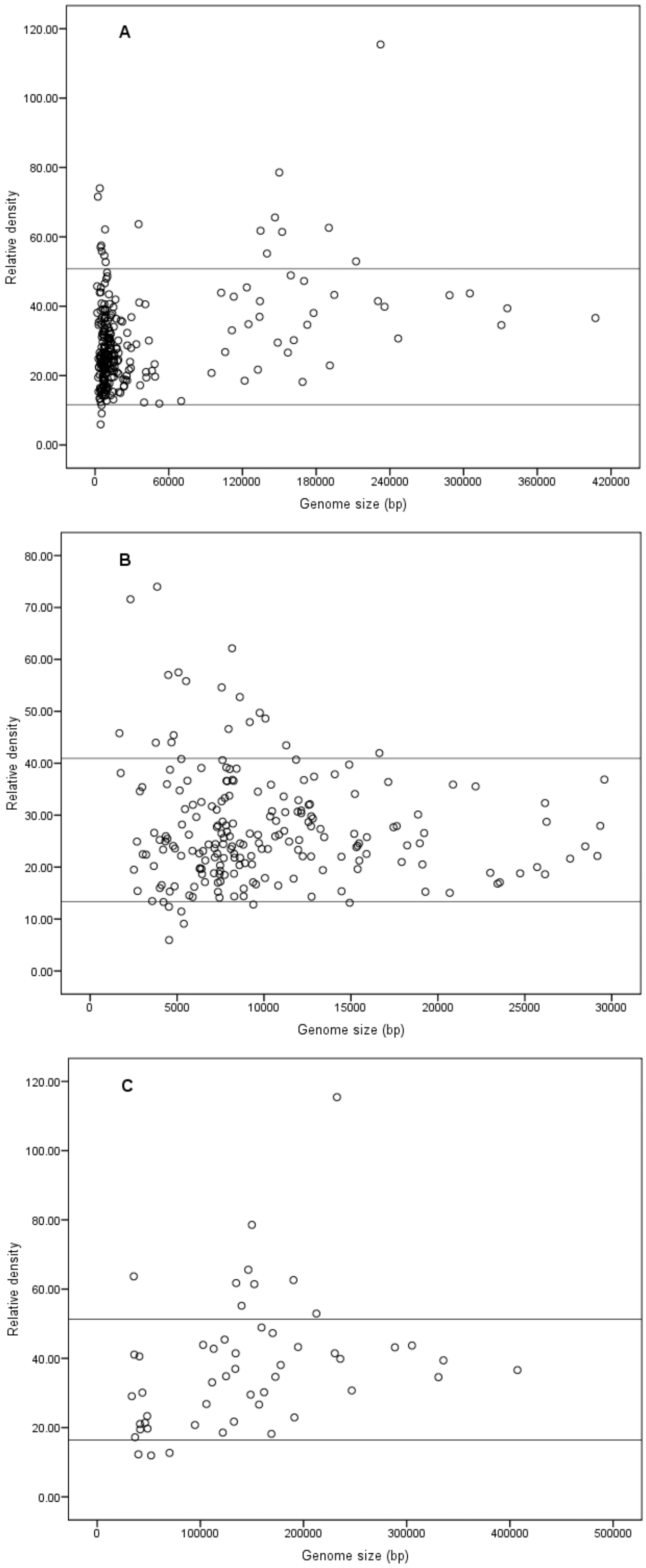

Additional file 14 Scatter plots of SSRs relative density versus genome size.

(*A*) Scatter plot of SSRs relative densities in all analysed virus genomes. (*B*) Scatter plot of SSRs relative densities in analysed virus genomes with size of < 30000 bp. (*C*) Scatter plot of

SSRs relative densities in analysed virus genomes with size of > 30000 bp.
