# Supplementary material for: Coevolution between simple sequence repeats (SSRs) and virus genome size
Source: BMC Genomics. 2012 Aug 30;13:435. doi: 10.1186/1471-2164-13-435 (PMC3585866; doi:10.1186/1471-2164-13-435)
Supplement: Additional file 8 — Descriptive statistics of SSRs variables. [file 1471-2164-13-435-S8.pdf]

Additional file 6 Descriptive statistics of SSRs variables

Parameters	Mean	Std. deviation	Minimum	Maximum
Mono-	76.008	191.514	0	1383
Di-	99.035	222.988	2	1855
Tri-	26.673	70.186	0	555
Tetra-	1.066	3.184	0	22
Penta-	1.187	0.693	0	6
Hexa-	0.486	2.714	0	27
