# Supplementary material for: Coevolution between simple sequence repeats (SSRs) and virus genome size
Source: BMC Genomics. 2012 Aug 30;13:435. doi: 10.1186/1471-2164-13-435 (PMC3585866; doi:10.1186/1471-2164-13-435)
Supplement: Additional file 9 — Matrix of correlation coefficients and 1-tailed tests between SSRs. [file 1471-2164-13-435-S9.pdf]

Additional file 7 Matrix of correlation coefficients and 1-tailed tests between SSRs

	Mono-	Di-	Tri-	Tetra-	Penta-	Hexa-
Mono-		**	**	**	**	**
Di-	0.790[a]		**	**	**	**
Tri-	0.742	0.867		**	**	**
Tetra-	0.698	0.796	0.771		**	**
Penta-	0.662	0.639	0.504	0.594		**
Hexa-	0.356	0.416	0.375	0.319	0.265	

[a] Correlation coefficients between repeat classes.

** Represents extremely significant difference (P<0.001) between variables.
