# Supplementary material for: Coevolution between simple sequence repeats (SSRs) and virus genome size
Source: BMC Genomics. 2012 Aug 30;13:435. doi: 10.1186/1471-2164-13-435 (PMC3585866; doi:10.1186/1471-2164-13-435)
Supplement: Additional file 10 — Scree plot. It displays the "cliff" and the "screes" vividly, which can be visually proved that the applicability of PCA is very good to the current data set. [file 1471-2164-13-435-S10.pdf]

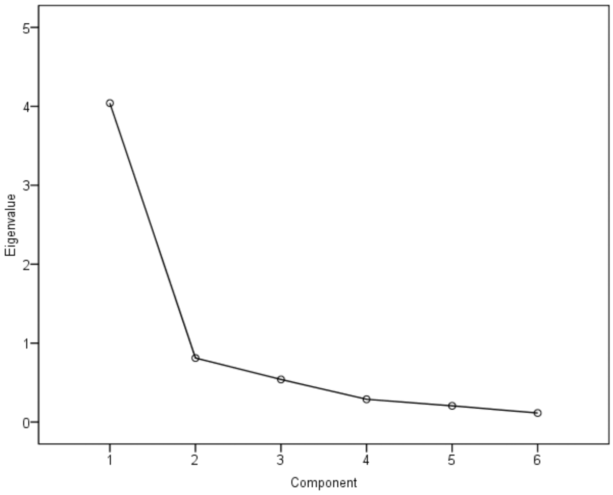

Additional file 15 Scree plot.

It displays the "cliff" and the "screes" vividly, which can be visually proved that the applicability of PCA is very good to the current data set.
