# Supplementary material for: Coevolution between simple sequence repeats (SSRs) and virus genome size
Source: BMC Genomics. 2012 Aug 30;13:435. doi: 10.1186/1471-2164-13-435 (PMC3585866; doi:10.1186/1471-2164-13-435)
Supplement: Additional file 11 — Occurrence of mono- SSRs in analyzed virus genomes. [file 1471-2164-13-435-S11.pdf]

Additional file 8 Occurrence of mono- SSRs in analyzed virus genomes

No. Type	Repeated motifs				Total mono-	A+T (%)	C+G (%)
	A	C	G	T (U)			
S1-dsDNA-1	79	0	0	112	191	100.00	0
S2-dsDNA-2	60	5	3	71	139	94.24	5.76
S3-dsDNA-3	34	0	2	37	73	97.26	2.74
S4-dsDNA-4	30	3	6	16	55	83.64	16.36
S5-dsDNA-5	90	3	4	52	149	95.30	4.70
S6-dsDNA-6	40	2	5	36	83	91.57	8.43
S7-dsDNA-7	35	0	2	29	66	96.97	3.03
S8-dsDNA-8	33	4	0	63	100	96.00	4.00
S9-dsDNA-9	32	1	1	30	64	96.88	3.13
S10-dsDNA-10	1	10	2	0	13	7.69	92.31
S11-dsDNA-11	34	1	1	36	72	97.22	2.78
S12-dsDNA-12	0	2	8	1	11	9.09	90.91
S13-dsDNA-13	0	0	0	1	1	100.00	0
S14-dsDNA-14	2	0	0	3	5	100.00	0
S15-dsDNA-15	23	0	3	20	46	93.48	6.52
S16-dsDNA-16	11	1	0	13	25	96.00	4.00
S17-dsDNA-17	12	0	0	3	15	100.00	0.00
S18-dsDNA-18	24	0	2	10	36	94.44	5.56
S19-dsDNA-19	17	0	0	4	21	100.00	0
S20-dsDNA-20	53	2	2	71	128	96.88	3.13
S21-dsDNA-21	25	2	1	16	44	93.18	6.82
S22-dsDNA-22	98	0	0	95	193	100.00	0
S23-dsDNA-23	8	0	0	12	20	100.00	0
S24-dsDNA-24	2	0	0	3	5	100.00	0
S25-dsDNA-25	237	0	2	256	495	99.60	0.40
S26-dsDNA-26	18	7	8	17	50	70.00	30.00
S27-dsDNA-27	379	5	5	335	724	98.62	1.38
S28-dsDNA-28	598	2	0	507	1107	99.82	0.18
S29-dsDNA-29	138	13	10	134	295	92.20	7.80
S30-dsDNA-30	275	0	1	260	536	99.81	0.19
S31-dsDNA-31	47	15	23	38	123	69.11	30.89
S32-dsDNA-32	432	4	2	398	836	99.28	0.72
S33-dsDNA-33	613	2	4	764	1383	99.57	0.43
S34-dsDNA-34	384	15	28	360	787	94.54	5.46
S35-dsDNA-35	476	10	2	566	1054	98.86	1.14
S36-dsDNA-36	135	7	12	132	286	93.36	6.64
S37-dsDNA-37	27	23	13	27	90	60.00	40.00
S38-dsDNA-38	174	1	0	230	405	99.75	0.25
S39-dsDNA-39	21	13	6	24	64	70.31	29.69

S40-dsDNA-40	381	29	24	388	822	93.55	6.45
S41-dsDNA-41	196	47	53	193	489	79.55	20.45
S42-dsDNA-42	373	18	14	363	768	95.83	4.17
S43-dsDNA-43	161	1	3	141	306	98.69	1.31
S44-dsDNA-44	101	11	8	86	206	90.78	9.22
S45-dsDNA-45	295	18	11	198	522	94.44	5.56
S46-dsDNA-46	63	68	81	70	282	47.16	52.84
S47-dsDNA-47	33	290	310	30	663	9.50	90.50
S48-dsDNA-48	80	58	46	99	283	63.25	36.75
S49-dsDNA-49	126	56	53	113	348	68.68	31.32
S50-dsDNA-50	76	36	30	103	245	73.06	26.94
S51-dsDNA-51	129	46	48	120	343	72.59	27.41
S52-dsDNA-52	49	60	63	67	239	48.54	51.46
S53-dsDNA-53	231	15	26	225	497	91.75	8.25
S54-dsDNA-54	18	166	210	26	420	10.48	89.52
S55-dsDNA-55	182	2	3	167	354	98.59	1.41
S56-dsDNA-56	24	13	14	17	68	60.29	39.71
S57-dsDNA-57	17	12	8	10	47	57.45	42.55
S58-dsDNA-58	56	2	0	54	112	98.21	1.79
S59-dsDNA-59	31	2	1	39	73	95.89	4.11
S60-dsDNA-60	10	1	0	10	21	95.24	4.76
S61-dsDNA-61	6	1	1	7	15	86.67	13.33
S62-dsDNA-62	5	2	3	6	16	68.75	31.25
S63-dsDNA-63	3	1	1	4	9	77.78	22.22
S64-dsDNA-64	6	1	3	3	13	69.23	30.77
S65-dsDNA-65	9	2	3	6	20	75.00	25.00
S66-dsDNA-66	4	6	10	7	27	40.74	59.26
S67-dsDNA-67	3	1	3	1	8	50.00	50.00
S68-dsDNA-68	1	1	2	2	6	50.00	50.00
S69-dsDNA-69	2	5	2	1	10	30.00	70.00
S70-dsDNA-70	3	3	5	6	17	52.94	47.06
S71-dsDNA-71	10	3	4	14	31	77.42	22.58
S72-dsDNA-72	6	0	0	5	11	100.00	0
S73-dsDNA-73	1	0	4	6	11	63.64	36.36
S74-dsDNA-74	4	2	1	4	11	72.73	27.27
S75-dsDNA-75	3	2	3	3	11	54.55	45.45
S76-dsDNA-76	213	5	2	249	469	98.51	1.49
S77-dsDNA-77	38	2	0	38	78	97.44	2.56
S78-ssDNA-1	5	0	0	1	6	100.00	0
S79-ssDNA-2	16	0	0	0	16	100.00	0
S80-ssDNA-3	1	0	0	3	4	100.00	0
S81-ssDNA-4	11	0	0	4	15	100.00	0
S82-ssDNA-5	14	1	0	0	15	93.33	6.67

S83-ssDNA-6	1	0	0	5	6	100.00	0
S84-ssDNA-7	1	0	1	1	3	66.67	33.33
S85-ssDNA-8	2	0	0	2	4	100.00	0
S86-ssDNA-9	1	0	1	2	4	75.00	25.00
S87-ssDNA-10	1	0	1	3	5	80.00	20.00
S88-ssDNA-11	0	1	1	2	4	50.00	50.00
S89-ssDNA-12	0	7	10	0	17	0	100.00
S90-ssDNA-13	8	7	5	2	22	45.45	54.55
S91-ssDNA-14	1	0	0	1	2	100.00	0
S92-ssDNA-15	5	2	0	0	7	71.43	28.57
S93-ssDNA-16	8	0	0	4	12	100.00	0
S94-ssDNA-17	8	0	3	5	16	81.25	18.75
S95-ssDNA-18	1	0	0	1	2	100.00	0
S96-ssDNA-19	4	0	2	1	7	71.43	28.57
S97-ssDNA-20	9	0	5	0	14	64.29	35.71
S98-ssDNA-21	5	0	0	2	7	100.00	0
S99-ssDNA-22	8	2	2	4	16	75.00	25.00
S100-ssDNA-23	8	0	0	1	9	100.00	0
S101-ssDNA-24	5	0	0	1	6	100.00	0
S102-dsDNA-RT-1	1	0	0	1	2	100.00	0
S103-dsDNA-RT-2	0	0	0	0	0	0	0
S104-dsDNA-RT-3	7	0	1	0	8	87.50	12.50
S105-dsDNA-RT-4	32	0	0	0	32	100.00	0
S106-dsDNA-RT-5	36	0	0	0	36	100.00	0
S107-dsDNA-RT-6	15	0	1	0	16	93.75	6.25
S108-dsDNA-RT-7	0	1	0	0	1	0	100.00
S109-dsDNA-RT-8	8	2	0	3	13	84.62	15.38
S110-ssRNA-RT-1	7	4	0	1	12	66.67	33.33
S111-ssRNA-RT-2	1	7	2	1	11	18.18	81.82
S112-ssRNA-RT-3	1	1	3	0	5	20.00	80.00
S113-ssRNA-RT-4	5	12	1	4	22	40.91	59.09
S114-ssRNA-RT-5	14	0	5	2	21	76.19	23.81
S115-ssRNA-RT-6	10	3	1	6	20	80.00	20.00
S116-ssRNA-RT-7	10	1	0	1	12	91.67	8.33
S117-dsRNA-1	3	0	1	0	4	75.00	25.00
S118-dsRNA-2	0	1	5	0	6	0	100.00
S119-dsRNA-3	12	0	3	6	21	85.71	14.29
S120-dsRNA-4	17	0	0	10	27	100.00	0
S121-dsRNA-5	2	3	2	9	16	68.75	31.25
S122-dsRNA-6	0	14	1	0	15	0	100.00
S123-dsRNA-7	0	0	1	0	1	0	100.00
S124-dsRNA-8	22	0	0	33	55	100.00	0
S125-dsRNA-9	11	0	0	5	16	100.00	0

S126-dsRNA-10	0	2	2	0	4	0	100.00
S127-dsRNA-11	3	1	0	1	5	80.00	20.00
S128-dsRNA-12	1	3	0	0	4	25.00	75.00
S129-dsRNA-13	0	4	1	0	5	0	100.00
S130-dsRNA-14	0	1	0	0	1	0	100.00
S131-dsRNA-15	1	3	1	2	7	42.86	57.14
S132-dsRNA-16	0	1	0	0	1	0	100.00
S133-dsRNA-17	2	1	4	0	7	28.57	71.43
S134-dsRNA-18	3	0	1	2	6	83.33	16.67
S135-dsRNA-19	3	2	1	4	10	70.00	30.00
S136-dsRNA-20	0	0	0	2	2	100.00	0
S137-dsRNA-21	9	0	1	7	17	94.12	5.88
S138-dsRNA-22	1	1	0	0	2	50.00	50.00
S139-dsRNA-23	17	3	2	1	23	78.26	21.74
S140-(-)ssRNA-1	6	0	4	0	10	60.00	40.00
S141-(-)ssRNA-2	9	2	0	8	19	89.47	10.53
S142-(-)ssRNA-3	11	1	5	4	21	71.43	28.57
S143-(-)ssRNA-4	34	0	2	5	41	95.12	4.88
S144-(-)ssRNA-5	6	1	2	0	9	66.67	33.33
S145-(-)ssRNA-6	2	0	3	1	6	50.00	50.00
S146-(-)ssRNA-7	15	2	1	2	20	85.00	15.00
S147-(-)ssRNA-8	13	2	0	8	23	91.30	8.70
S148-(-)ssRNA-9	17	1	0	8	26	96.15	3.85
S149-(-)ssRNA-10	1	2	2	0	5	20.00	80.00
S150-(-)ssRNA-11	8	7	6	3	24	45.83	54.17
S151-(-)ssRNA-12	11	1	4	4	20	75.00	25.00
S152-(-)ssRNA-13	6	1	2	6	15	80.00	20.00
S153-(-)ssRNA-14	10	5	5	2	22	54.55	45.45
S154-(-)ssRNA-15	34	0	0	3	37	100.00	0
S155-(-)ssRNA-16	19	0	3	5	27	88.89	11.11
S156-(-)ssRNA-17	13	1	1	2	17	88.24	11.76
S157-(-)ssRNA-18	7	0	0	24	31	100.00	0
S158-(-)ssRNA-19	4	0	2	2	8	75.00	25.00
S159-(-)ssRNA-20	17	0	0	3	20	100.00	0
S160-(-)ssRNA-21	6	1	1	1	9	77.78	22.22
S161-(-)ssRNA-22	12	0	2	1	15	86.67	13.33
S162-(-)ssRNA-23	4	0	1	2	7	85.71	14.29
S163-(-)ssRNA-24	16	0	0	1	17	100.00	0
S164-(-)ssRNA-25	6	1	1	11	19	89.47	10.53
S165-(-)ssRNA-26	29	0	2	9	40	95.00	5.00
S166-(-)ssRNA-27	1	4	3	0	8	12.50	87.50
S167-(-)ssRNA-28	34	0	1	24	59	98.31	1.69
S168-(-)ssRNA-29	16	1	0	11	28	96.43	3.57

S169-(-)ssRNA-30	4	1	3	2	10	60.00	40.00
S170-(-)ssRNA-31	1	4	1	0	6	16.67	83.33
S171-(+)ssRNA-1	0	1	1	0	2	0	100.00
S172-(+)ssRNA-2	0	1	1	0	2	0	100.00
S173-(+)ssRNA-3	0	1	2	0	3	0	100.00
S174-(+)ssRNA-4	0	0	0	0	0	0	0
S175-(+)ssRNA-5	2	1	0	0	3	66.67	33.33
S176-(+)ssRNA-6	1	0	1	0	2	50.00	50.00
S177-(+)ssRNA-7	3	0	1	5	9	88.89	11.11
S178-(+)ssRNA-8	1	6	0	1	8	25.00	75.00
S179-(+)ssRNA-9	1	1	0	0	2	50.00	50.00
S180-(+)ssRNA-10	4	0	1	1	6	83.33	16.67
S181-(+)ssRNA-11	1	2	0	0	3	33.33	66.67
S182-(+)ssRNA-12	0	15	1	0	16	0	100.00
S183-(+)ssRNA-13	1	1	0	0	2	50.00	50.00
S184-(+)ssRNA-14	4	1	0	6	11	90.91	9.09
S185-(+)ssRNA-15	2	0	0	2	4	100.00	0
S186-(+)ssRNA-16	1	1	0	4	6	83.33	16.67
S187-(+)ssRNA-17	1	1	1	1	4	50.00	50.00
S188-(+)ssRNA-18	1	1	1	0	3	33.33	66.67
S189-(+)ssRNA-19	1	3	1	6	11	63.64	36.36
S190-(+)ssRNA-20	2	0	0	10	12	100.00	0
S191-(+)ssRNA-21	0	0	0	2	2	100.00	0
S192-(+)ssRNA-22	3	0	1	5	9	88.89	11.11
S193-(+)ssRNA-23	1	3	0	4	8	62.50	37.50
S194-(+)ssRNA-24	1	0	0	0	1	100.00	0
S195-(+)ssRNA-25	1	0	0	0	1	100.00	0
S196-(+)ssRNA-26	1	2	1	0	4	25.00	75.00
S197-(+)ssRNA-27	5	0	1	0	6	83.33	16.67
S198-(+)ssRNA-28	1	0	0	0	1	100.00	0
S199-(+)ssRNA-29	3	2	0	0	5	60.00	40.00
S200-(+)ssRNA-30	0	0	1	0	1	0	100.00
S201-(+)ssRNA-31	0	6	2	0	8	0	100.00
S202-(+)ssRNA-32	0	3	0	0	3	0	100.00
S203-(+)ssRNA-33	1	4	0	0	5	20.00	80.00
S204-(+)ssRNA-34	6	0	1	2	9	88.89	11.11
S205-(+)ssRNA-35	5	0	0	6	11	100.00	0
S206-(+)ssRNA-36	0	0	0	0	0	0	0
S207-(+)ssRNA-37	0	1	0	0	1	0	100.00
S208-(+)ssRNA-38	1	3	1	0	5	20.00	80.00
S209-(+)ssRNA-39	0	1	1	0	2	0	100.00
S210-(+)ssRNA-40	1	3	0	1	5	40.00	60.00
S211-(+)ssRNA-41	0	1	0	0	1	0	100.00

S212-(+)ssRNA-42	0	4	1	0	5	0	100.00
S213-(+)ssRNA-43	0	0	2	0	2	0	100.00
S214-(+)ssRNA-44	0	1	3	0	4	0	100.00
S215-(+)ssRNA-45	0	0	4	0	4	0	100.00
S216-(+)ssRNA-46	1	0	0	0	1	100.00	0
S217-(+)ssRNA-47	0	1	1	0	2	0	100.00
S218-(+)ssRNA-48	0	0	1	0	1	0	100.00
S219-(+)ssRNA-49	0	0	4	0	4	0	100.00
S220-(+)ssRNA-50	0	2	2	0	4	0	100.00
S221-(+)ssRNA-51	0	1	0	0	1	0	100.00
S222-(+)ssRNA-52	4	1	1	4	10	80.00	20.00
S223-(+)ssRNA-53	4	1	2	32	39	92.31	7.69
S224-(+)ssRNA-54	1	2	0	1	4	50.00	50.00
S225-(+)ssRNA-55	6	0	0	4	10	100.00	0
S226-(+)ssRNA-56	14	0	3	2	19	84.21	15.79
S227-(+)ssRNA-57	0	8	8	4	20	20.00	80.00
S228-(+)ssRNA-58	5	1	0	2	8	87.50	12.50
S229-(+)ssRNA-59	0	13	2	0	15	0	100.00
S230-(+)ssRNA-60	4	1	1	2	8	75.00	25.00
S231-(+)ssRNA-61	3	0	3	3	9	66.67	33.33
S232-(+)ssRNA-62	4	0	0	2	6	100.00	0
S233-(+)ssRNA-63	5	2	0	3	10	80.00	20.00
S234-(+)ssRNA-64	10	3	0	9	22	86.36	13.64
S235-(+)ssRNA-65	7	1	0	3	11	90.91	9.09
S236-(+)ssRNA-66	1	0	0	3	4	100.00	0
S237-(+)ssRNA-67	2	0	0	4	6	100.00	0
S238-(+)ssRNA-68	3	0	1	0	4	75.00	25.00
S239-(+)ssRNA-69	2	0	0	1	3	100.00	0
S240-(+)ssRNA-70	0	3	0	1	4	25.00	75.00
S241-(+)ssRNA-71	5	1	0	4	10	90.00	10.00
S242-(+)ssRNA-72	0	2	1	0	3	0	100.00
S243-(+)ssRNA-73	1	1	0	1	3	66.67	33.33
S244-(+)ssRNA-74	0	33	0	0	33	0	100.00
S245-(+)ssRNA-75	0	8	0	0	8	0	100.00
S246-(+)ssRNA-76	0	4	0	0	4	0	100.00
S247-(+)ssRNA-77	4	0	1	7	12	91.67	8.33
S248-(+)ssRNA-78	5	1	0	5	11	90.91	9.09
S249-(+)ssRNA-79	3	1	4	8	16	68.75	31.25
S250-(+)ssRNA-80	1	0	0	0	1	100.00	0
S251-(+)ssRNA-81	0	1	0	0	1	0	100.00
S252-(+)ssRNA-82	0	1	1	0	2	0	100.00
S253-(+)ssRNA-83	4	0	0	1	5	100.00	0
S254-(+)ssRNA-84	8	0	0	3	11	100.00	0

S255-(+)ssRNA-85	0	0	1	0	1	0	100.00
S256-(+)ssRNA-86	6	0	1	2	9	88.89	11.11
S257-(+)ssRNA-87	0	1	1	0	2	0	100.00
