# Supplementary material for: Coevolution between simple sequence repeats (SSRs) and virus genome size
Source: BMC Genomics. 2012 Aug 30;13:435. doi: 10.1186/1471-2164-13-435 (PMC3585866; doi:10.1186/1471-2164-13-435)
Supplement: Additional file 12 — Occurrence of di- SSRs in analyzed virus genomes. [file 1471-2164-13-435-S12.pdf]

Additional file 9 Occurrence of di- SSRs in analyzed virus genomes

No. Type	Repeated motifs						Total
	AC/CA	AG/GA	AT/TA	CG/GC	CT/TC	GT/TG	
S1-dsDNA-1	29	17	119	1	22	23	211
S2-dsDNA-2	21	10	24	43	12	19	129
S3-dsDNA-3	6	3	14	22	6	10	61
S4-dsDNA-4	3	4	4	10	8	7	36
S5-dsDNA-5	27	70	85	0	31	42	255
S6-dsDNA-6	16	13	12	6	14	11	72
S7-dsDNA-7	1	11	20	20	4	7	63
S8-dsDNA-8	11	31	103	8	27	23	203
S9-dsDNA-9	8	7	13	0	8	1	37
S10-dsDNA-10	7	9	0	7	11	12	46
S11-dsDNA-11	11	11	6	24	1	6	59
S12-dsDNA-12	6	6	0	78	7	5	102
S13-dsDNA-13	14	18	3	3	15	9	62
S14-dsDNA-14	3	4	14	0	4	10	35
S15-dsDNA-15	10	12	11	1	10	9	53
S16-dsDNA-16	7	14	15	0	13	12	61
S17-dsDNA-17	0	0	4	0	0	0	4
S18-dsDNA-18	5	5	6	12	0	3	31
S19-dsDNA-19	1	1	15	0	0	1	18
S20-dsDNA-20	4	13	61	0	22	8	108
S21-dsDNA-21	5	18	28	0	7	6	64
S22-dsDNA-22	4	15	96	0	10	3	128
S23-dsDNA-23	7	6	16	0	7	0	36
S24-dsDNA-24	6	3	10	2	3	5	29
S25-dsDNA-25	45	89	391	5	112	34	676
S26-dsDNA-26	40	62	13	648	62	33	858
S27-dsDNA-27	34	45	812	11	44	42	988
S28-dsDNA-28	25	12	420	0	10	25	492
S29-dsDNA-29	55	53	148	28	47	53	384
S30-dsDNA-30	24	27	668	0	10	23	752
S31-dsDNA-31	108	55	6	1036	30	114	1349
S32-dsDNA-32	27	29	228	2	26	25	337
S33-dsDNA-33	40	11	1764	0	6	34	1855
S34-dsDNA-34	34	28	165	19	25	36	307
S35-dsDNA-35	43	23	284	2	33	32	417
S36-dsDNA-36	42	28	24	48	33	45	220
S37-dsDNA-37	20	94	34	6	78	27	259
S38-dsDNA-38	20	2	155	3	10	15	205
S39-dsDNA-39	151	16	39	60	18	119	403

S40-dsDNA-40	109	76	357	31	78	95	746
S41-dsDNA-41	193	183	64	145	168	246	999
S42-dsDNA-42	142	60	535	105	39	117	998
S43-dsDNA-43	53	19	93	51	21	72	309
S44-dsDNA-44	152	30	45	39	33	137	436
S45-dsDNA-45	117	152	136	9	125	102	641
S46-dsDNA-46	69	64	40	105	79	52	409
S47-dsDNA-47	57	23	24	300	16	48	468
S48-dsDNA-48	67	22	106	39	31	50	315
S49-dsDNA-49	58	53	196	30	52	53	442
S50-dsDNA-50	43	64	50	106	65	38	366
S51-dsDNA-51	114	62	70	291	53	95	685
S52-dsDNA-52	94	101	56	236	112	81	680
S53-dsDNA-53	83	67	102	37	64	77	430
S54-dsDNA-54	60	73	14	44	75	68	334
S55-dsDNA-55	58	24	134	2	38	53	309
S56-dsDNA-56	11	6	5	53	9	11	95
S57-dsDNA-57	13	16	14	36	20	9	108
S58-dsDNA-58	8	5	25	0	4	3	45
S59-dsDNA-59	3	16	10	3	9	4	45
S60-dsDNA-60	2	0	0	0	2	2	6
S61-dsDNA-61	4	0	12	0	0	16	32
S62-dsDNA-62	5	3	8	0	1	1	18
S63-dsDNA-63	0	2	9	0	1	3	15
S64-dsDNA-64	2	4	2	1	4	2	15
S65-dsDNA-65	4	3	2	1	2	3	15
S66-dsDNA-66	0	1	3	2	2	6	14
S67-dsDNA-67	0	3	3	0	1	1	8
S68-dsDNA-68	0	4	5	1	3	3	16
S69-dsDNA-69	3	1	8	1	1	1	15
S70-dsDNA-70	4	1	1	0	1	3	10
S71-dsDNA-71	6	3	4	1	4	5	23
S72-dsDNA-72	1	4	9	0	0	4	18
S73-dsDNA-73	3	4	5	0	0	2	14
S74-dsDNA-74	5	5	2	0	0	2	14
S75-dsDNA-75	3	4	9	1	1	7	25
S76-dsDNA-76	87	63	170	27	68	80	495
S77-dsDNA-77	117	36	32	35	22	112	354
S78-ssDNA-1	0	0	4	0	3	0	7
S79-ssDNA-2	4	1	8	0	3	0	16
S80-ssDNA-3	0	1	0	1	0	2	4
S81-ssDNA-4	0	1	4	0	0	3	8
S82-ssDNA-5	5	0	1	0	1	0	7

S83-ssDNA-6	0	1	3	0	0	0	4
S84-ssDNA-7	0	2	0	1	1	1	5
S85-ssDNA-8	0	1	2	0	3	0	6
S86-ssDNA-9	0	1	7	0	1	2	11
S87-ssDNA-10	1	0	2	1	2	1	7
S88-ssDNA-11	0	0	0	0	1	1	2
S89-ssDNA-12	0	1	3	0	2	1	7
S90-ssDNA-13	5	0	1	6	0	1	13
S91-ssDNA-14	0	10	8	0	6	4	28
S92-ssDNA-15	3	3	8	0	3	2	19
S93-ssDNA-16	4	1	3	3	0	1	12
S94-ssDNA-17	2	0	3	2	1	6	14
S95-ssDNA-18	3	5	0	4	4	0	16
S96-ssDNA-19	4	3	8	0	1	1	17
S97-ssDNA-20	8	9	2	5	2	2	28
S98-ssDNA-21	2	0	6	0	3	0	11
S99-ssDNA-22	3	4	8	2	1	0	18
S100-ssDNA-23	2	1	8	0	0	0	11
S101-ssDNA-24	1	0	7	1	0	0	9
S102-dsDNA-RT-1	0	1	1	0	5	0	7
S103-dsDNA-RT-2	0	0	2	0	3	0	5
S104-dsDNA-RT-3	0	2	5	0	6	2	15
S105-dsDNA-RT-4	3	2	7	0	0	0	12
S106-dsDNA-RT-5	1	2	23	0	0	1	27
S107-dsDNA-RT-6	1	7	12	0	0	1	21
S108-dsDNA-RT-7	2	1	2	0	4	1	10
S109-dsDNA-RT-8	1	1	2	0	5	1	10
S110-ssRNA-RT-1	1	6	3	0	4	2	16
S111-ssRNA-RT-2	0	6	1	2	6	1	16
S112-ssRNA-RT-3	4	6	1	3	6	3	23
S113-ssRNA-RT-4	1	3	4	1	16	1	26
S114-ssRNA-RT-5	7	10	4	1	5	5	32
S115-ssRNA-RT-6	4	4	9	1	4	0	22
S116-ssRNA-RT-7	3	7	12	0	3	5	30
S117-dsRNA-1	2	3	4	6	10	4	29
S118-dsRNA-2	6	11	11	10	4	5	47
S119-dsRNA-3	6	11	13	7	4	4	45
S120-dsRNA-4	2	16	15	0	3	3	39
S121-dsRNA-5	9	22	6	3	11	12	63
S122-dsRNA-6	4	2	2	13	13	9	43
S123-dsRNA-7	13	8	26	5	2	10	64
S124-dsRNA-8	4	10	26	0	4	8	52
S125-dsRNA-9	5	6	26	1	2	7	47

S126-dsRNA-10	6	26	12	5	3	9	61
S127-dsRNA-11	4	6	11	1	1	7	30
S128-dsRNA-12	5	5	14	3	13	6	46
S129-dsRNA-13	4	6	0	1	1	1	13
S130-dsRNA-14	6	2	0	0	2	0	10
S131-dsRNA-15	1	4	4	0	1	0	10
S132-dsRNA-16	4	0	1	0	0	4	9
S133-dsRNA-17	2	3	3	0	2	2	12
S134-dsRNA-18	5	2	1	0	1	5	14
S135-dsRNA-19	2	2	3	0	4	1	12
S136-dsRNA-20	3	0	1	1	3	0	8
S137-dsRNA-21	4	11	2	2	0	4	23
S138-dsRNA-22	3	2	1	5	5	2	18
S139-dsRNA-23	19	9	2	3	0	8	41
S140-(-)ssRNA-1	5	3	6	0	2	1	17
S141-(-)ssRNA-2	4	7	7	0	5	2	25
S142-(-)ssRNA-3	2	16	3	0	7	6	34
S143-(-)ssRNA-4	1	17	15	0	1	3	37
S144-(-)ssRNA-5	4	16	5	0	7	6	38
S145-(-)ssRNA-6	1	7	8	0	8	4	28
S146-(-)ssRNA-7	3	9	2	0	6	2	22
S147-(-)ssRNA-8	9	7	11	0	3	7	37
S148-(-)ssRNA-9	8	10	11	0	5	4	38
S149-(-)ssRNA-10	4	15	8	0	8	3	38
S150-(-)ssRNA-11	6	5	5	0	5	6	27
S151-(-)ssRNA-12	6	6	10	0	9	8	39
S152-(-)ssRNA-13	5	18	12	1	7	1	44
S153-(-)ssRNA-14	2	9	11	0	11	4	37
S154-(-)ssRNA-15	15	6	14	0	3	0	38
S155-(-)ssRNA-16	8	17	8	0	3	8	44
S156-(-)ssRNA-17	1	27	7	0	13	1	49
S157-(-)ssRNA-18	3	1	15	0	9	4	32
S158-(-)ssRNA-19	9	15	4	0	6	5	39
S159-(-)ssRNA-20	4	10	10	0	6	0	30
S160-(-)ssRNA-21	8	16	3	0	6	4	37
S161-(-)ssRNA-22	4	12	3	0	1	2	22
S162-(-)ssRNA-23	4	14	4	0	4	8	34
S163-(-)ssRNA-24	1	10	19	0	3	4	37
S164-(-)ssRNA-25	7	11	9	1	2	4	34
S165-(-)ssRNA-26	8	15	7	0	5	3	38
S166-(-)ssRNA-27	2	15	5	0	9	5	36
S167-(-)ssRNA-28	7	15	9	0	4	2	37
S168-(-)ssRNA-29	10	11	20	0	14	7	62

S169-(-)ssRNA-30	3	4	4	0	4	3	18
S170-(-)ssRNA-31	0	3	0	0	2	0	5
S171-(+)ssRNA-1	0	2	0	1	3	0	6
S172-(+)ssRNA-2	0	1	0	1	2	0	4
S173-(+)ssRNA-3	0	0	0	1	2	0	3
S174-(+)ssRNA-4	0	2	3	0	2	0	7
S175-(+)ssRNA-5	5	4	0	1	4	1	15
S176-(+)ssRNA-6	9	3	8	0	0	3	23
S177-(+)ssRNA-7	0	3	0	0	3	3	9
S178-(+)ssRNA-8	2	8	1	1	3	3	18
S179-(+)ssRNA-9	9	5	1	4	0	4	23
S180-(+)ssRNA-10	3	5	2	0	0	2	12
S181-(+)ssRNA-11	5	2	0	2	4	3	16
S182-(+)ssRNA-12	1	0	0	4	15	3	23
S183-(+)ssRNA-13	3	3	0	0	5	5	16
S184-(+)ssRNA-14	1	5	11	2	1	5	25
S185-(+)ssRNA-15	7	10	5	0	3	1	26
S186-(+)ssRNA-16	4	1	1	6	1	3	16
S187-(+)ssRNA-17	5	8	1	0	9	5	28
S188-(+)ssRNA-18	4	7	7	2	2	10	32
S189-(+)ssRNA-19	1	3	10	5	11	9	39
S190-(+)ssRNA-20	2	7	5	1	11	3	29
S191-(+)ssRNA-21	6	2	2	0	6	2	18
S192-(+)ssRNA-22	1	9	4	0	6	3	23
S193-(+)ssRNA-23	3	4	3	2	11	7	30
S194-(+)ssRNA-24	8	10	5	0	3	6	32
S195-(+)ssRNA-25	9	4	3	3	0	2	21
S196-(+)ssRNA-26	13	4	3	4	5	5	34
S197-(+)ssRNA-27	1	3	2	3	0	7	16
S198-(+)ssRNA-28	4	3	2	2	0	5	16
S199-(+)ssRNA-29	6	2	1	0	3	0	12
S200-(+)ssRNA-30	4	3	0	1	0	7	15
S201-(+)ssRNA-31	6	3	1	0	3	2	15
S202-(+)ssRNA-32	8	2	0	4	0	3	17
S203-(+)ssRNA-33	0	1	0	2	6	2	11
S204-(+)ssRNA-34	4	3	1	1	1	3	13
S205-(+)ssRNA-35	7	3	2	1	2	4	19
S206-(+)ssRNA-36	1	0	0	2	0	0	3
S207-(+)ssRNA-37	1	0	0	0	2	2	5
S208-(+)ssRNA-38	3	3	2	2	0	0	10
S209-(+)ssRNA-39	1	2	0	2	1	5	11
S210-(+)ssRNA-40	1	5	3	0	2	2	13
S211-(+)ssRNA-41	1	3	1	3	1	1	10

S212-(+)ssRNA-42	0	3	0	0	2	0	5
S213-(+)ssRNA-43	4	4	1	2	1	3	15
S214-(+)ssRNA-44	2	2	1	0	3	4	12
S215-(+)ssRNA-45	1	1	2	0	1	0	5
S216-(+)ssRNA-46	4	0	1	1	2	1	9
S217-(+)ssRNA-47	4	4	1	0	2	2	13
S218-(+)ssRNA-48	1	2	0	0	1	6	10
S219-(+)ssRNA-49	1	2	1	1	2	3	10
S220-(+)ssRNA-50	4	1	1	0	2	1	9
S221-(+)ssRNA-51	5	4	0	5	4	10	28
S222-(+)ssRNA-52	10	10	10	0	6	30	66
S223-(+)ssRNA-53	3	9	12	0	1	24	49
S224-(+)ssRNA-54	54	2	6	0	29	2	93
S225-(+)ssRNA-55	4	13	0	0	3	9	29
S226-(+)ssRNA-56	6	12	5	0	3	5	31
S227-(+)ssRNA-57	2	2	0	4	4	4	16
S228-(+)ssRNA-58	6	4	1	2	0	4	17
S229-(+)ssRNA-59	3	0	0	41	1	2	47
S230-(+)ssRNA-60	4	8	2	2	1	4	21
S231-(+)ssRNA-61	3	15	2	1	1	3	25
S232-(+)ssRNA-62	1	7	3	0	2	2	15
S233-(+)ssRNA-63	3	6	2	2	3	7	23
S234-(+)ssRNA-64	2	12	3	0	2	4	23
S235-(+)ssRNA-65	2	12	5	1	2	11	33
S236-(+)ssRNA-66	5	4	9	1	1	13	33
S237-(+)ssRNA-67	1	7	7	0	1	0	16
S238-(+)ssRNA-68	5	6	4	0	1	8	24
S239-(+)ssRNA-69	3	4	2	1	9	5	24
S240-(+)ssRNA-70	2	3	4	3	4	6	22
S241-(+)ssRNA-71	1	6	0	1	5	0	13
S242-(+)ssRNA-72	0	1	0	0	4	0	5
S243-(+)ssRNA-73	1	6	5	1	4	4	21
S244-(+)ssRNA-74	0	0	0	1	9	0	10
S245-(+)ssRNA-75	2	0	0	0	8	0	10
S246-(+)ssRNA-76	1	0	0	3	8	0	12
S247-(+)ssRNA-77	0	5	2	11	9	5	32
S248-(+)ssRNA-78	2	8	16	0	3	7	36
S249-(+)ssRNA-79	2	7	8	4	4	9	34
S250-(+)ssRNA-80	3	5	1	0	2	2	13
S251-(+)ssRNA-81	14	3	0	3	8	1	29
S252-(+)ssRNA-82	9	2	2	0	4	3	20
S253-(+)ssRNA-83	0	7	3	0	3	7	20
S254-(+)ssRNA-84	0	7	1	0	1	1	10

S255-(+)ssRNA-85	4	3	2	1	3	1	14
S256-(+)ssRNA-86	4	7	1	0	0	7	19
S257-(+)ssRNA-87	3	1	0	0	0	0	4
