# Supplementary material for: Coevolution between simple sequence repeats (SSRs) and virus genome size
Source: BMC Genomics. 2012 Aug 30;13:435. doi: 10.1186/1471-2164-13-435 (PMC3585866; doi:10.1186/1471-2164-13-435)
Supplement: Additional file 13 — Occurrence of tri- SSRs in analyzed virus genomes. [file 1471-2164-13-435-S13.pdf]

Additional file 10 Occurrence of tri- SSRs in analyzed virus genomes

No. Type	Repeat motif groups										Total
	**aat ata att taa tat tta**	**aac aca caa gtt tgt ttg**	**aag aga ctt gaa tct ttc**	**acc cac cca ggt gtg tgg**	**acg cga cgt gac gtc tcg**	**agt act cta gta tac tag**	**agc cag ctg gca gct tgc**	**agg cct ctc gag gga tcc**	**atg atc cat gat tca tga**	**ggc ccg cgc cgg gcc gcg**	
S1-dsDNA-1	27	7	13	2			3		15		67
S2-dsDNA-2	2	5	3	1	3	1	3	4	8	3	33
S3-dsDNA-3	2			1			3		2	7	15
S4-dsDNA-4		1			1		2		1	3	8
S5-dsDNA-5	7	4	10			2	1	6	7		37
S6-dsDNA-6	3	1	3	2	3		1	1	2	3	19
S7-dsDNA-7		2	1			1	1		7	4	16
S8-dsDNA-8	19	4	7	1		2	5	1	6	1	46
S9-dsDNA-9	6	1	3			3			2		15
S10-dsDNA-10			4	3	7	1	3	2	2	5	27
S11-dsDNA-11		2	1				5	2	5	3	18
S12-dsDNA-12			4	1	9		4			2	20
S13-dsDNA-13	1		1	1	3	1	2		3		12
S14-dsDNA-14	1	2	1			1				1	6
S15-dsDNA-15	1	2	6	3	2		5		1	1	21
S16-dsDNA-16		3	7	3		4	17		4		38
S17-dsDNA-17	1	4								3	8
S18-dsDNA-18	2	4								2	8
S19-dsDNA-19	2								1		3
S20-dsDNA-20	8	2	5	1		1		1			18
S21-dsDNA-21		1	5	1							7
S22-dsDNA-22	7	1	7	1	1	2					19
S23-dsDNA-23	2					1					3
S24-dsDNA-24											0
S25-dsDNA-25	36	5	15	1	8	10	1	6	25	1	108
S26-dsDNA-26	2	6	10	15	19	5	32	15	3	66	173
S27-dsDNA-27	89	9	27	3	2	13	2	3	21		169
S28-dsDNA-28	69	6	10	1	1	4		3	21		115
S29-dsDNA-29	9	9	11	2	20	2		5	1	1	60
S30-dsDNA-30	80	9	8			6		1	16		120
S31-dsDNA-31		6	18	10	22	4	38	5	6	48	157
S32-dsDNA-32	30	14	7	1	4	4	1	2	2	1	66
S33-dsDNA-33	510	4	11		1	3	1	1	23		554
S34-dsDNA-34	31	8	11	2		2	2	9	12	3	80
S35-dsDNA-35	65	18	55	1	7	5	2	4	29		186
S36-dsDNA-36	16	13	9	16	15	7	12	13	4	17	122
S37-dsDNA-37		1	5	7	5	1	21	11	2	11	64

S38-dsDNA-38	36	9	4	3	2		1		7		62
S39-dsDNA-39	2	12	1	12	7	2	14	4	8	16	78
S40-dsDNA-40	33	19	37	6	7	4	9	2	23	1	141
S41-dsDNA-41	11	26	44	27	47	2	40	21	59	29	306
S42-dsDNA-42	54	39	29	144	19	6	16	12	28	41	388
S43-dsDNA-43	18	18	5	5	20	4	3	3	5	20	101
S44-dsDNA-44	7	25	6	38	14	13	6	9	23	7	148
S45-dsDNA-45	41	96	157	23	12	31	56	80	53	6	555
S46-dsDNA-46	7	4	6	9	3		4	14	24	7	78
S47-dsDNA-47		3	4	20	31	2	12	15	1	73	161
S48-dsDNA-48	9	3	4	3	6			6	2	6	39
S49-dsDNA-49	15	9	6	1	4	1	2	8	8	2	56
S50-dsDNA-50	8	3	9	6	3	3	5	11	4	8	60
S51-dsDNA-51	3	13	17	43	45	5	25	26	22	97	296
S52-dsDNA-52	6	22	24	24	63	11	41	41	29	94	355
S53-dsDNA-53	4	11	7	3	4	3	5	7	7	7	58
S54-dsDNA-54	1	4	3	17	1	1	23	41	7	34	132
S55-dsDNA-55	11	7	31	1		2	9	1	5		67
S56-dsDNA-56	2	1	4	4			4	6	3	18	42
S57-dsDNA-57		1	4	5	4		4	10	1	7	36
S58-dsDNA-58	2		5			3		1			11
S59-dsDNA-59		3	3	2			1		2		11
S60-dsDNA-60		1					1		2		4
S61-dsDNA-61		2	1				2	2			7
S62-dsDNA-62	1							2			3
S63-dsDNA-63			1				1	4			6
S64-dsDNA-64							1				1
S65-dsDNA-65						1	1	3	1		6
S66-dsDNA-66		1	1				1	1		1	5
S67-dsDNA-67			1				2	1			4
S68-dsDNA-68				1						2	3
S69-dsDNA-69				1			1	1		1	4
S70-dsDNA-70			1				1	1	1		4
S71-dsDNA-71	1	1	1	1				1			5
S72-dsDNA-72	1	1	1					1			4
S73-dsDNA-73	1	1					1	1	2	1	7
S74-dsDNA-74	2	1		2				2			7
S75-dsDNA-75	1	3		1					1		6
S76-dsDNA-76	31	12	23	4	4	1	8	10	18	3	114
S77-dsDNA-77	5	21	6	30	26	2	3	2	19	7	121
S78-ssDNA-1	1			1	1	1				1	5
S79-ssDNA-2		4				1					5
S80-ssDNA-3											0

S81-ssDNA-4	1									1
S82-ssDNA-5			1		1		1		1		4
S83-ssDNA-6	1	1	3								5
S84-ssDNA-7			1					1			2
S85-ssDNA-8	1	1	2				1				5
S86-ssDNA-9		1	1								2
S87-ssDNA-10		2	1								3
S88-ssDNA-11		1						1			2
S89-ssDNA-12									1		1
S90-ssDNA-13	1	1		1		1	1	1			6
S91-ssDNA-14	4		5					1			10
S92-ssDNA-15	6		2						1		9
S93-ssDNA-16				1			1		1		3
S94-ssDNA-17							1	1			2
S95-ssDNA-18		2		1	1			1			5
S96-ssDNA-19		1	2	2		1					6
S97-ssDNA-20	1	2					1			1	5
S98-ssDNA-21	3				1		1		3		8
S99-ssDNA-22	2		1	4				1			8
S100-ssDNA-23		1	2				1				4
S101-ssDNA-24	1		1	4				1			7
S102-dsDNA-RT-1			1					1			2
S103-dsDNA-RT-2			2	2							4
S104-dsDNA-RT-3			6				1				7
S105-dsDNA-RT-4	1		2					1			4
S106-dsDNA-RT-5	6		5								11
S107-dsDNA-RT-6	1		2			1					4
S108-dsDNA-RT-7	1	1	4				1				7
S109-dsDNA-RT-8	1		3								4
S110-ssRNA-RT-1	2								1		3
S111-ssRNA-RT-2	1							1			2
S112-ssRNA-RT-3				2				1			3
S113-ssRNA-RT-4	1	1									2
S114-ssRNA-RT-5	2	1	2			2	3	2			12
S115-ssRNA-RT-6	1	2	1					3			7
S116-ssRNA-RT-7	1	1	4	1		1		1	1		10
S117-dsRNA-1	1	1		2			1				5
S118-dsRNA-2	1		1	1	1		1		3		8
S119-dsRNA-3			1				1	4	5		11
S120-dsRNA-4	4		1				1		2		8
S121-dsRNA-5	1	4			1		1	3	4	2	16
S122-dsRNA-6		1	1		1		1	2	1	2	9
S123-dsRNA-7	1	2				1	1		2		7

S124-dsRNA-8	7	3	4			1		1	2		18
S125-dsRNA-9	2	1	4	1		1			3	1	13
S126-dsRNA-10		2			1	1		2	3		9
S127-dsRNA-11	1	5		1		1	1		1		10
S128-dsRNA-12	1	1	1		1	1	1		2		8
S129-dsRNA-13		1		1		1					3
S130-dsRNA-14			1				1				2
S131-dsRNA-15			1								1
S132-dsRNA-16									1		1
S133-dsRNA-17		1						1			2
S134-dsRNA-18		1	1	1							3
S135-dsRNA-19				1		2					3
S136-dsRNA-20							1				1
S137-dsRNA-21		8	1	3				1	2		15
S138-dsRNA-22				1	1		1	2			5
S139-dsRNA-23		2	2	2		1	2				9
S140-(-)ssRNA-1				1				1			2
S141-(-)ssRNA-2	1	1							1		3
S142-(-)ssRNA-3								1		1	2
S143-(-)ssRNA-4	1		1					1	5		8
S144-(-)ssRNA-5	1		3	1			1		2		8
S145-(-)ssRNA-6	1		1				2	2	3		9
S146-(-)ssRNA-7		2	2		1		1	5			11
S147-(-)ssRNA-8								1	2		3
S148-(-)ssRNA-9	1	1		1	1				2		6
S149-(-)ssRNA-10			1					2	1		4
S150-(-)ssRNA-11					1			1	2		4
S151-(-)ssRNA-12	1										1
S152-(-)ssRNA-13		1				2		2	4		9
S153-(-)ssRNA-14	1	2	1								4
S154-(-)ssRNA-15	1		2	1					1		5
S155-(-)ssRNA-16	1	3	1	1	1		2	1			10
S156-(-)ssRNA-17			3					2	3		8
S157-(-)ssRNA-18	2		3					1	3		9
S158-(-)ssRNA-19	1	1	2						2		6
S159-(-)ssRNA-20			1				1	2	2		6
S160-(-)ssRNA-21			1	3							4
S161-(-)ssRNA-22	1	3	2					1	2		9
S162-(-)ssRNA-23	1	1	5	1			2				10
S163-(-)ssRNA-24	1	2	3						5		11
S164-(-)ssRNA-25	1	1	1	1		5	2		4		15
S165-(-)ssRNA-26			2	2		1	1	1	2		9
S166-(-)ssRNA-27		1	2				2	4	4		13

S167-(-)ssRNA-28		1	4					1	2		8
S168-(-)ssRNA-29	2	1	3								6
S169-(-)ssRNA-30			1								1
S170-(-)ssRNA-31								1			1
S171-(+)ssRNA-1											0
S172-(+)ssRNA-2			1							1	2
S173-(+)ssRNA-3					1						1
S174-(+)ssRNA-4											0
S175-(+)ssRNA-5			2	1							3
S176-(+)ssRNA-6				1							1
S177-(+)ssRNA-7	2							1	1		4
S178-(+)ssRNA-8		1	1					1		1	4
S179-(+)ssRNA-9			1	1					1		3
S180-(+)ssRNA-10											0
S181-(+)ssRNA-11									1		1
S182-(+)ssRNA-12					1		1				2
S183-(+)ssRNA-13			1				1				2
S184-(+)ssRNA-14							2		1		3
S185-(+)ssRNA-15	2	3	1								6
S186-(+)ssRNA-16			1				1		1	1	4
S187-(+)ssRNA-17	1			1				1			3
S188-(+)ssRNA-18			1								1
S189-(+)ssRNA-19			1	1			2	2	1		7
S190-(+)ssRNA-20		2		2				1			5
S191-(+)ssRNA-21		2	1								3
S192-(+)ssRNA-22								1			1
S193-(+)ssRNA-23			2						1		3
S194-(+)ssRNA-24		1				1					2
S195-(+)ssRNA-25			1	1			1				3
S196-(+)ssRNA-26		2	1						5		8
S197-(+)ssRNA-27		1	1	2					1		5
S198-(+)ssRNA-28		1	1								2
S199-(+)ssRNA-29									1		1
S200-(+)ssRNA-30									1		1
S201-(+)ssRNA-31			1					1	3		5
S202-(+)ssRNA-32			1	1							2
S203-(+)ssRNA-33				2			1	1		2	6
S204-(+)ssRNA-34		1					1		1		3
S205-(+)ssRNA-35		1		1					2		4
S206-(+)ssRNA-36		1									1
S207-(+)ssRNA-37	1							1			2
S208-(+)ssRNA-38		2		1		1		1			5
S209-(+)ssRNA-39				1					1		2

S210-(+)ssRNA-40		1	1						1		3
S211-(+)ssRNA-41							1	1		1	3
S212-(+)ssRNA-42			1			1					2
S213-(+)ssRNA-43		1									1
S214-(+)ssRNA-44											0
S215-(+)ssRNA-45		1	1	1			1				4
S216-(+)ssRNA-46											0
S217-(+)ssRNA-47										1	1
S218-(+)ssRNA-48											0
S219-(+)ssRNA-49	1		1						1		3
S220-(+)ssRNA-50		1		1		1					3
S221-(+)ssRNA-51		2	1	2	1		1		3	1	11
S222-(+)ssRNA-52	1	5	2	2		2	1		1		14
S223-(+)ssRNA-53	5	5		2		1			1		14
S224-(+)ssRNA-54		5	1	4		3			1		14
S225-(+)ssRNA-55			1	1				1	2		5
S226-(+)ssRNA-56	4	1	3			2					10
S227-(+)ssRNA-57			1	1			1		1		4
S228-(+)ssRNA-58			1	2			1		1		5
S229-(+)ssRNA-59				2				1		6	9
S230-(+)ssRNA-60		1							2		3
S231-(+)ssRNA-61	1	1	2	1		2					7
S232-(+)ssRNA-62		2	3	2							7
S233-(+)ssRNA-63	1		3	2	1	1			2		10
S234-(+)ssRNA-64	2	1		2			4				9
S235-(+)ssRNA-65	1	2	4	1	1			1	1		11
S236-(+)ssRNA-66	2	3	3	2		1	3	1	2		17
S237-(+)ssRNA-67			1				1				2
S238-(+)ssRNA-68		1		1							2
S239-(+)ssRNA-69	2	1	1	1					1		6
S240-(+)ssRNA-70		2	1	1					1		5
S241-(+)ssRNA-71		2	1								3
S242-(+)ssRNA-72			1					1		1	3
S243-(+)ssRNA-73		1							1		2
S244-(+)ssRNA-74		1	2	1				6		1	11
S245-(+)ssRNA-75					1						1
S246-(+)ssRNA-76								3			3
S247-(+)ssRNA-77			3	2		1					6
S248-(+)ssRNA-78	1	1	1			1	1		2		7
S249-(+)ssRNA-79	1	2	2		1		2				8
S250-(+)ssRNA-80		2		1			1				4
S251-(+)ssRNA-81				1	2	1		2			6
S252-(+)ssRNA-82											0

S253-(+)ssRNA-83	1		2				1				4
S254-(+)ssRNA-84			1								1
S255-(+)ssRNA-85			2								2
S256-(+)ssRNA-86								1	2		3
S257-(+)ssRNA-87			2		1						3
