# Supplementary material for: Coevolution between simple sequence repeats (SSRs) and virus genome size
Source: BMC Genomics. 2012 Aug 30;13:435. doi: 10.1186/1471-2164-13-435 (PMC3585866; doi:10.1186/1471-2164-13-435)
Supplement: Additional file 14 — Occurrence of tetra-, penta- and hexa- SSRs in analyzed virus genomes. [file 1471-2164-13-435-S14.pdf]

Additional file 11 Occurrence of tetra-, penta- and hexa- SSRs in analyzed virus genomes

No. Type	Tetra-		Penta-		Hexa-	
	Total	Repeat motifs	Total	Repeat motifs	Total	Repeat motifs
S1-dsDNA-1	1	tctt (1)	1	tttaa (1)	1	tcgtgt (1)
S2-dsDNA-2	0		0		0	
S3-dsDNA-3	1	gttt (1)	0		0	
S4-dsDNA-4	0		0		0	
S5-dsDNA-5	0		0		0	
S6-dsDNA-6	0		0		0	
S7-dsDNA-7	1	cgac (1)	0		0	
S8-dsDNA-8	1	aatt (1)	0		0	
S9-dsDNA-9	1	aaga (1)	0		0	
S10-dsDNA-10	1	cggc (1)	0		0	
S11-dsDNA-11	0		1	ggcgc (1)	0	
S12-dsDNA-12	0		0		0	
S13-dsDNA-13	0		0		0	
S14-dsDNA-14	0		0		0	
S15-dsDNA-15	0		0		0	
S16-dsDNA-16	2	aaat (1); ttca (1)	0		0	
S17-dsDNA-17	0		0		0	
S18-dsDNA-18	0		0		0	
S19-dsDNA-19	0		0		0	
S20-dsDNA-20	0		0		0	
S21-dsDNA-21	1	aaag (1)	0		0	
S22-dsDNA-22	0		2	aatat (1); atatt (1)	0	
S23-dsDNA-23	0		0		0	
S24-dsDNA-24	1	cgtg (1)	0		0	
S25-dsDNA-25	5	atag (1); atta (1); tgaa (1); ttat (1); ttta (1)	1	aattc (1)	2	aatagg (1); ttctta (1)
S26-dsDNA-26	20	actc (3); agcg (1); cact (1); ccca (1); cgag (2); ctcg (4); gagc (1); gagt (4); gtga (1); taat (1); tcac (1)	0		2	agttac (1); acactc (1)
S27-dsDNA-27	10	aata (1); aatt (1); agtt (1); atac (1); gatg (1); tatt (1); tctt (1); ttac (1); ttat (1); ttgg (1)	1	attat (1)	0	
S28-dsDNA-28	8	aaat (2); aatt (1);	1	atttt (1)	1	gataat (1)

		tca (1); attt (1); gatg (1); ttta (2)				
S29-dsDNA-29	4	aaat (1); ggta (1); gttc (1); tacg (1)	0		0	
S30-dsDNA-30	8	aata (1); agtg (1); cact (1); gatg (1); taca (1); tatt (1); ttta (2)	1	tgaaa (1)	3	atcctt (1); aatgac (1); ataagg (1)
S31-dsDNA-31	3	ccgc (1); gagc (1); tgcg (1)	2	gcgcg (1); cgcgc (1)	3	ctgcgc (1); gagcgc (1); tccacg (1)
S32-dsDNA-32	2	caaa (1); aatg (1)	0		0	
S33-dsDNA-33	22	aaac (1); aaat (3); aata (3); aatt (3); ataa (3); atac (1); attt (4); tatt (1); ttat (2); tttc (1)	6	atata (2); taata (2); tattt (1); ttata (1)	3	taaata (1); tatatt (1); tcaggt (1)
S34-dsDNA-34	6	ataa (1); atca (2); attt (1); tgat (2)	0		0	
S35-dsDNA-35	7	aaat (2); aata (1); gaaa (1); gttt (1); tgtt (1); ttga (1)	2	atttt (1); atatt (1)	1	gaaacg (1)
S36-dsDNA-36	6	aaat (1); ataa (1); gttg (1); tcga (1); ttga (1); tttg (1)	1	tcgct (1)	1	cggctg (1)
S37-dsDNA-37	0		0		0	
S38-dsDNA-38	1	aatt (1)	2	tttat (1); ataat (1)	2	atattt (1); ttaaaa (1)
S39-dsDNA-39	1	accg (1)	0		0	
S40-dsDNA-40	2	caaa (1); gttt (1)	4	ataat (1); atacg (1); gcgga (1); gctcc (1)	2	ggtcct (1); tgttat (1)
S41-dsDNA-41	11	aaag (1); aatt (2); accg (1); atgt (1); cggt (1); taaa (1); taat (1); ttaa (2); ttat (1)	1	cctac (1)	19	actgaa (1); cagcaa (1); ccgacc (1); ccgatg (1); cgacat (1); ctcctt (1); gaggcg (1); gatggc (2); gcagat (1); ggcgga (1); ggtgcc (1); ggtggc (2); tcactt (1); tgccga (1); tgcgtt (1); tggcgc (1); tttcag (1)
S42-dsDNA-42	13	aaag (1); aatt (3); accg (1); atgt (1); cggt (1); taaa (1); taat (1); ttaa (3); ttat (1)	0		3	ctgcat (1); tcatcg (1); tggagg (1)
S43-dsDNA-43	4	ataa (1); gttt (1);	2	aaaat (1); aatta (1)	3	aggtgt (2); ggcgga (1)

		tatt (1); ttta (1)				
S44-dsDNA-44	9	aaat (2); acaa (1); ggtg (1); gtgg (1); tgat (1); tggt (1); ttta (2)	1	ataat (1)	1	tcatct (1)
S45-dsDNA-45	5	aacc (1); aaga (1); atgc (1); ttct (1); ttgg (1)	0		8	accaga (1); aggagt (2); atctgg (1); cagcaa (1); cgatga (1); cttcct (1); gaggaa (1)
S46-dsDNA-46	4	attc (1); cacg (1); gagg (2)	3	ggcgc (1); ttgtc (2)	2	tccgag (1); tgcccg (1)
S47-dsDNA-47	19	caaa (1); cacc (1); ccca (1); cccg (1); ccgg (1); cgcc (1); cggc (1); ggcg (1); ggtg (2); gtgg (7); tggg (1); ttta (1)	4	cccgg (2); ggccg (2)	4	tggggc (2); tggggt (1); ttgggg (1)
S48-dsDNA-48	5	acag (1); accg (1); cggt (1); gttg (1); tgtc (1)	2	tgggg (1); cccca (1)	1	gaggac (1)
S49-dsDNA-49	5	atag (1); attt (1); taaa (1); tatg (1); tctt (1)	2	aatat (1); tatat (1)	26	aaccct (9); acccta (3); agggtt (2); ccctaa (1); cctaac (1); ctaacc (2); gggtta (4); ggttag (1); taaccc (1); tagggt (2)
S50-dsDNA-50	3	aaag (1); agcc (1); tgtt (1)	0		0	
S51-dsDNA-51	2	gtcc (1); tgta (1)	0		0	
S52-dsDNA-52	18	acgg (1); agct (1); agtt (1); catc (1); cgag (1); cgtt (1); ctgt (1); gacc (1); gcgg (1); ggac (1); ggga (1); gtga (1); gtgc (1); tatc (1); tcac (1); tcga (1); tcgc (1); tttc (1)	0		5	cggcag (1); ggcgtc (1); tcaggc (1); tgatgc (1); tggcgg (1)
S53-dsDNA-53	1	tgtt (1)	0		27	aaccct (1); acccta (6); ccctaa (2); cctaac (5); ctaacc (2); gtatgt (1); taaccc (1); tgtatg (3); tgtgta (6)

S54-dsDNA-54	5	ttat (1); ccca (1); ggcg (3)	0		0	
S55-dsDNA-55	2	aaat (1); aaca (1)	0		0	
S56-dsDNA-56	0		0		1	gcggca (1)
S57-dsDNA-57	0		0		0	
S58-dsDNA-58	0		0		0	
S59-dsDNA-59	0		0		0	
S60-dsDNA-60	0		0		0	
S61-dsDNA-61	0		0		0	
S62-dsDNA-62	0		0		0	
S63-dsDNA-63	0		0		0	
S64-dsDNA-64	0		0		0	
S65-dsDNA-65	0		0		0	
S66-dsDNA-66	0		0		0	
S67-dsDNA-67	0		0		0	
S68-dsDNA-68	0		0		0	
S69-dsDNA-69	0		0		0	
S70-dsDNA-70	0		0		0	
S71-dsDNA-71	1	aggg (1)	0		0	
S72-dsDNA-72	0		0		0	
S73-dsDNA-73	0		0		0	
S74-dsDNA-74	0		0		0	
S75-dsDNA-75	0		0		0	
S76-dsDNA-76	17	aatc (1); ataa (1); atgc (1); cata (1); ccat (2); cctt (1); ctca (2); gtat (1); taca (1); taaa (2); tgaa (1); tgga (1); ttaa (1); ttgt (1)	3	ttaac (2) tatag (1)	1	attttt (1)
S77-dsDNA-77	6	cacc (1); ccac (1); cgat (1); cgtc (1); gtat (1); taaa (1)	0		0	
S78-ssDNA-1	0		0		0	
S79-ssDNA-2	0		0		0	
S80-ssDNA-3	0		0		0	
S81-ssDNA-4	0		0		0	
S82-ssDNA-5	0		0		0	
S83-ssDNA-6	0		0		0	
S84-ssDNA-7	0		0		0	
S85-ssDNA-8	0		0		0	
S86-ssDNA-9	0		0		0	
S87-ssDNA-10	0		0		0	

S88-ssDNA-11	1	ttta (1)	0		0	
S89-ssDNA-12	0		0		0	
S90-ssDNA-13	0		0		0	
S91-ssDNA-14	0		1	taaat (1)	0	
S92-ssDNA-15	0		0		0	
S93-ssDNA-16	0		0		0	
S94-ssDNA-17	0		0		0	
S95-ssDNA-18	4	cgct (2); gagc (2)	0		0	
S96-ssDNA-19	0		0		0	
S97-ssDNA-20	0		0		0	
S98-ssDNA-21	0		0		0	
S99-ssDNA-22	1	taaa (1)	0		0	
S100-ssDNA-23	0		0		0	
S101-ssDNA-24	0		0		0	
S102-dsDNA-RT-1	0		0		0	
S103-dsDNA-RT-2	0		0		0	
S104-dsDNA-RT-3	0		0		0	
S105-dsDNA-RT-4	0		0		0	
S106-dsDNA-RT-5	1	gaaa (1)	0		0	
S107-dsDNA-RT-6	0		0		0	
S108-dsDNA-RT-7	0		0		0	
S109-dsDNA-RT-8	0		0		0	
S110-ssRNA-RT-1	1	ccta (1)	0		0	
S111-ssRNA-RT-2	0		0		0	
S112-ssRNA-RT-3	0		0		0	
S113-ssRNA-RT-4	0		0		0	
S114-ssRNA-RT-5	0		0		0	
S115-ssRNA-RT-6	1	aaat (1)	0		0	
S116-ssRNA-RT-7	0		0		1	agaggt (1)
S117-dsRNA-1	0		0		0	
S118-dsRNA-2	0		0		0	
S119-dsRNA-3	0		0		0	
S120-dsRNA-4	0		0		0	
S121-dsRNA-5	1	ccgt (1)	0		0	
S122-dsRNA-6	0		0		0	
S123-dsRNA-7	0		0		0	
S124-dsRNA-8	0		0		0	
S125-dsRNA-9	0		0		0	
S126-dsRNA-10	0		0		0	
S127-dsRNA-11	0		0		0	
S128-dsRNA-12	1	atac (1)	0		0	
S129-dsRNA-13	0		0		0	
S130-dsRNA-14	0		0		0	

S131-dsRNA-15	0		0		0	
S132-dsRNA-16	0		0		0	
S133-dsRNA-17	0		0		0	
S134-dsRNA-18	0		0		0	
S135-dsRNA-19	0		0		0	
S136-dsRNA-20	0		0		0	
S137-dsRNA-21	0		0		0	
S138-dsRNA-22	0		0		0	
S139-dsRNA-23	0		0		0	
S140-(-)ssRNA-1	0		0		0	
S141-(-)ssRNA-2	0		0		0	
S142-(-)ssRNA-3	0		0		0	
S143-(-)ssRNA-4	1	caat (1)	0		0	
S144-(-)ssRNA-5	0		0		0	
S145-(-)ssRNA-6	0		0		0	
S146-(-)ssRNA-7	0		0		0	
S147-(-)ssRNA-8	0		0		0	
S148-(-)ssRNA-9	2	aata (1); tcga (1)	0		0	
S149-(-)ssRNA-10	0		0		0	
S150-(-)ssRNA-11	0		0		0	
S151-(-)ssRNA-12	0		0		0	
S152-(-)ssRNA-13	0		0		0	
S153-(-)ssRNA-14	0		0		0	
S154-(-)ssRNA-15	0		0		0	
S155-(-)ssRNA-16	0		0		0	
S156-(-)ssRNA-17	0		0		0	
S157-(-)ssRNA-18	0		0		0	
S158-(-)ssRNA-19	0		0		0	
S159-(-)ssRNA-20	0		0		0	
S160-(-)ssRNA-21	0		0		0	
S161-(-)ssRNA-22	1	aagc (1)	0		0	
S162-(-)ssRNA-23	1	agga (1)	0		0	
S163-(-)ssRNA-24	1	tgta (1)	0		0	
S164-(-)ssRNA-25	1	cttt (1)	0		0	
S165-(-)ssRNA-26	0		0		0	
S166-(-)ssRNA-27	0		0		0	
S167-(-)ssRNA-28	0		2	taaaa (1); tttta (1)	0	
S168-(-)ssRNA-29	0		0		0	
S169-(-)ssRNA-30	0		0		0	
S170-(-)ssRNA-31	0		0		0	
S171-(+)ssRNA-1	0		0		0	
S172-(+)ssRNA-2	0		0		0	
S173-(+)ssRNA-3	0		0		0	

S174-(+)ssRNA-4	0		0		0	
S175-(+)ssRNA-5	0		0		0	
S176-(+)ssRNA-6	0		0		0	
S177-(+)ssRNA-7	0		0		0	
S178-(+)ssRNA-8	0		0		0	
S179-(+)ssRNA-9	0		0		0	
S180-(+)ssRNA-10	0		0		0	
S181-(+)ssRNA-11	0		0		0	
S182-(+)ssRNA-12	3	ctcc (1); tcct (1); ttag (1)	0		0	
S183-(+)ssRNA-13	0		0		0	
S184-(+)ssRNA-14	0		0		0	
S185-(+)ssRNA-15	0		0		0	
S186-(+)ssRNA-16	0		0		0	
S187-(+)ssRNA-17	0		0		0	
S188-(+)ssRNA-18	0		0		0	
S189-(+)ssRNA-19	0		0		0	
S190-(+)ssRNA-20	0		0		0	
S191-(+)ssRNA-21	0		0		0	
S192-(+)ssRNA-22	0		0		0	
S193-(+)ssRNA-23	2	tgtc (2)	0		0	
S194-(+)ssRNA-24	0		0		1	gcagag (1)
S195-(+)ssRNA-25	0		0		0	
S196-(+)ssRNA-26	0		2	aaaca (2)	0	
S197-(+)ssRNA-27	0		0		0	
S198-(+)ssRNA-28	0		0		0	
S199-(+)ssRNA-29	0		0		0	
S200-(+)ssRNA-30	0		0		0	
S201-(+)ssRNA-31	0		0		0	
S202-(+)ssRNA-32	1	ggtt (1)	0		0	
S203-(+)ssRNA-33	0		0		0	
S204-(+)ssRNA-34	0		0		0	
S205-(+)ssRNA-35	0		0		0	
S206-(+)ssRNA-36	0		0		0	
S207-(+)ssRNA-37	0		0		0	
S208-(+)ssRNA-38	0		0		0	
S209-(+)ssRNA-39	0		0		0	
S210-(+)ssRNA-40	1	cctc (1)	0		0	
S211-(+)ssRNA-41	0		0		0	
S212-(+)ssRNA-42	0		0		0	
S213-(+)ssRNA-43	0		0		0	
S214-(+)ssRNA-44	0		0		0	
S215-(+)ssRNA-45	0		0		0	

S216-(+)ssRNA-46	0		0		0	
S217-(+)ssRNA-47	0		0		0	
S218-(+)ssRNA-48	0		0		0	
S219-(+)ssRNA-49	0		0		0	
S220-(+)ssRNA-50	0		0		0	
S221-(+)ssRNA-51	0		0		0	
S222-(+)ssRNA-52	0		0		0	
S223-(+)ssRNA-53	0		0		0	
S224-(+)ssRNA-54	0		0		0	
S225-(+)ssRNA-55	0		0		0	
S226-(+)ssRNA-56	0		0		0	
S227-(+)ssRNA-57	0		0		0	
S228-(+)ssRNA-58	1	gaaa (1)	0		0	
S229-(+)ssRNA-59	0		0		0	
S230-(+)ssRNA-60	0		0		0	
S231-(+)ssRNA-61	0		0		0	
S232-(+)ssRNA-62	0		0		0	
S233-(+)ssRNA-63	0		0		0	
S234-(+)ssRNA-64	0		0		0	
S235-(+)ssRNA-65	0		0		0	
S236-(+)ssRNA-66	0		0		1	tataat (1)
S237-(+)ssRNA-67	0		0		0	
S238-(+)ssRNA-68	0		0		0	
S239-(+)ssRNA-69	0		0		0	
S240-(+)ssRNA-70	0		0		0	
S241-(+)ssRNA-71	0		0		0	
S242-(+)ssRNA-72	0		0		0	
S243-(+)ssRNA-73	0		0		0	
S244-(+)ssRNA-74	0		0		0	
S245-(+)ssRNA-75	0		0		0	
S246-(+)ssRNA-76	0		0		0	
S247-(+)ssRNA-77	0		0		0	
S248-(+)ssRNA-78	1	aaac (1)	0		0	
S249-(+)ssRNA-79	0		0		0	
S250-(+)ssRNA-80	0		0		0	
S251-(+)ssRNA-81	1	ccac (1)	0		0	
S252-(+)ssRNA-82	0		0		0	
S253-(+)ssRNA-83	0		0		0	
S254-(+)ssRNA-84	1	gaag (1)	0		0	
S255-(+)ssRNA-85	0		0		0	
S256-(+)ssRNA-86	0		0		0	
S257-(+)ssRNA-87	0		0		0	
