# Supplementary material for: Coevolution between simple sequence repeats (SSRs) and virus genome size
Source: BMC Genomics. 2012 Aug 30;13:435. doi: 10.1186/1471-2164-13-435 (PMC3585866; doi:10.1186/1471-2164-13-435)
Supplement: Additional file 15 — Hosts of analyzed virus genomes. [file 1471-2164-13-435-S15.pdf]

Additional file 12 Hosts of analyzed virus genomes

No. Type	Genome size (bp)	Occurrence	Length (bp)	SSRs (%)	Relative abundance	Relative density	Host	Coding S (%)	Gene No.	GC content (%)
S1-dsDNA-1	168903	472	3072	1.82	2.79	18.19	archaea, bacteria	93	288	35
S2-dsDNA-2	94800	301	1967	2.07	3.18	20.75	bacteria	86	117	47
S3-dsDNA-3	33593	150	976	2.91	4.47	29.05	bacteria	92	43	50
S4-dsDNA-4	36717	99	632	1.72	2.70	17.21	bacteria	94	55	52
S5-dsDNA-5	132562	441	2878	2.17	3.33	21.71	bacteria	89	209	39
S6-dsDNA-6	48502	174	1131	2.33	3.59	23.32	bacteria	87	92	49
S7-dsDNA-7	48836	146	962	1.97	2.99	19.70	bacteria	91	78	45
S8-dsDNA-8	121750	350	2258	1.85	2.87	18.55	bacteria	79	195	39
S9-dsDNA-9	22172	117	788	3.55	5.28	35.54	bacteria	92	41	36
S10-dsDNA-10	52297	87	625	1.20	1.66	11.95	bacteria	87	88	62
S11-dsDNA-11	46375	150	992	2.14	3.23	21.39	bacteria	90	60	51
S12-dsDNA-12	41491	133	873	2.10	3.21	21.04	archaea, bacteria	89	54	63
S13-dsDNA-13	39937	75	490	1.23	1.88	12.27	bacteria	92	60	48
S14-dsDNA-14	19282	46	294	1.52	2.39	15.25	bacteria	93	27	39
S15-dsDNA-15	41724	120	812	1.95	2.88	19.46	bacteria	90	74	47
S16-dsDNA-16	70153	126	890	1.27	1.80	12.69	bacteria	94	72	41
S17-dsDNA-17	14927	27	196	1.31	1.81	13.13	bacteria	95	31	48
S18-dsDNA-18	10079	75	490	4.86	7.44	48.62	bacteria	92	22	42
S19-dsDNA-19	11965	42	279	2.33	3.51	23.32	archaea, bacteria	82	14	31
S20-dsDNA-20	40900	254	1659	4.06	6.21	40.56	archaea	88	73	33
S21-dsDNA-21	20869	116	749	3.59	5.56	35.89	archaea	91	40	36
S22-dsDNA-22	35450	342	2257	6.37	9.65	63.67	archaea	79	54	25
S23-dsDNA-23	15465	59	380	2.46	3.82	24.57	archaea	90	33	39
S24-dsDNA-24	14462	35	222	1.54	2.42	15.35	archaea	91	35	38
S25-dsDNA-25	194711	1287	8423	4.33	6.61	43.26	vertebrates	88	223	33
S26-dsDNA-26	139962	1103	7725	5.52	7.88	55.19	vertebrates	89	130	63
S27-dsDNA-27	288539	1892	12458	4.32	6.56	43.18	vertebrates	84	260	30
S28-dsDNA-28	149955	1724	11780	7.86	11.50	78.56	vertebrates	93	148	25
S29-dsDNA-29	161773	743	4887	3.02	4.59	30.21	vertebrates	95	170	43
S30-dsDNA-30	146454	1420	9605	6.56	9.70	65.58	vertebrates	96	150	27
S31-dsDNA-31	190289	1637	11915	6.26	8.60	62.62	vertebrates	85	163	63
S32-dsDNA-32	134721	1241	8320	6.18	9.21	61.76	vertebrates	94	140	29
S33-dsDNA-33	232392	3823	26829	11.54	16.45	115.45	invertebrates	91	294	17
S34-dsDNA-34	170101	1180	8043	4.73	6.94	47.28	vertebrates	87	151	38
S35-dsDNA-35	212482	1667	11247	5.29	7.85	52.93	invertebrates	90	463	28
S36-dsDNA-36	191100	636	4381	2.29	3.33	22.93	invertebrates	68	126	47

S37-dsDNA-37	105903	413	2838	2.68	3.90	26.80	vertebrates	79	99	55
S38-dsDNA-38	102653	677	4505	4.39	6.60	43.89	vertebrates	92	110	29
S39-dsDNA-39	111362	546	3682	3.31	4.90	33.06	vertebrates	93	125	54
S40-dsDNA-40	330743	1717	11432	3.46	5.19	34.56	algae	90	699	39
S41-dsDNA-41	335593	1825	13227	3.94	5.44	39.41	algae	70	240	51
S42-dsDNA-42	407339	2170	14913	3.66	5.33	36.61	algae	90	478	40
S43-dsDNA-43	133894	725	4947	3.69	5.41	36.95	invertebrates	91	156	40
S44-dsDNA-44	123500	801	5610	4.54	6.49	45.43	invertebrates	88	143	45
S45-dsDNA-45	305107	1731	13335	4.37	5.67	43.71	invertebrates	92	531	41
S46-dsDNA-46	134226	778	5563	4.14	5.80	41.45	vertebrates	83	90	56
S47-dsDNA-47	152261	1319	9352	6.14	8.66	61.42	vertebrates	79	77	68
S48-dsDNA-48	124884	645	4350	3.48	5.16	34.83	vertebrates	89	73	46
S49-dsDNA-49	177874	879	6770	3.81	4.94	38.06	vertebrates	73	87	44
S50-dsDNA-50	148687	674	4388	2.95	4.53	29.51	vertebrates	81	79	48
S51-dsDNA-51	235646	1326	9391	3.99	5.63	39.85	vertebrates	79	167	57
S52-dsDNA-52	230278	1297	9550	4.15	5.63	41.47	vertebrates	72	168	58
S53-dsDNA-53	159322	1013	7792	4.89	6.36	48.91	vertebrates	79	88	42
S54-dsDNA-54	172764	891	5988	3.47	5.16	34.66	vertebrates	68	82	59
S55-dsDNA-55	112930	732	4829	4.28	6.48	42.76	vertebrates	87	76	34
S56-dsDNA-56	35937	206	1476	4.11	5.73	41.07	vertebrates	90	18	55
S57-dsDNA-57	43804	191	1318	3.01	4.36	30.09	vertebrates	84	N/A	54
S58-dsDNA-58	29576	168	1090	3.69	5.68	36.85	vertebrates	92	31	33
S59-dsDNA-59	26163	129	846	3.23	4.93	32.34	vertebrates	92	23	37
S60-dsDNA-60	5243	31	214	4.08	5.91	40.82	vertebrates	89	8	40
S61-dsDNA-61	7961	54	371	4.66	6.78	46.60	vertebrates	84	6	40
S62-dsDNA-62	7746	37	258	3.33	4.78	33.31	vertebrates	92	8	42
S63-dsDNA-63	7353	30	206	2.80	4.08	28.02	vertebrates	92	7	38
S64-dsDNA-64	8095	29	190	2.35	3.58	23.47	vertebrates	91	15	47
S65-dsDNA-65	7841	41	287	3.66	5.23	36.60	vertebrates	86	6	44
S66-dsDNA-66	7610	46	309	4.06	6.04	40.60	vertebrates	87	7	52
S67-dsDNA-67	7729	20	143	1.85	2.59	18.50	vertebrates	89	6	47
S68-dsDNA-68	7304	25	165	2.26	3.42	22.59	vertebrates	93	6	49
S69-dsDNA-69	7687	29	197	2.56	3.77	25.63	vertebrates	92	6	50
S70-dsDNA-70	7868	31	211	2.68	3.94	26.82	vertebrates	90	10	46
S71-dsDNA-71	8607	60	454	5.27	6.97	52.75	vertebrates	77	7	41
S72-dsDNA-72	7815	33	219	2.80	4.22	28.02	vertebrates	86	7	40
S73-dsDNA-73	7614	32	219	2.88	4.20	28.76	vertebrates	90	11	46
S74-dsDNA-74	7276	32	226	3.11	4.40	31.06	vertebrates	91	8	43
S75-dsDNA-75	7879	42	288	3.66	5.33	36.55	vertebrates	89	8	46
S76-dsDNA-76	246734	1099	7577	3.07	4.45	30.71	invertebrates	N/A	N/A	41
S77-dsDNA-77	156922	559	4181	2.66	3.56	26.64	invertebrates	68	123	49
S78-ssDNA-1	6407	18	126	1.97	2.81	19.67	bacteria	91	10	40
S79-ssDNA-2	4491	37	256	5.70	8.24	57.00	bacteria	48	4	33

S80-ssDNA-3	5386	8	49	0.91	1.49	9.10	bacteria	95	11	44
S81-ssDNA-4	4421	24	159	3.60	5.43	35.96	bacteria	93	9	32
S82-ssDNA-5	4594	26	178	3.87	5.66	38.75	bacteria	86	11	46
S83-ssDNA-6	4877	15	115	2.36	3.08	23.58	bacteria	94	12	36
S84-ssDNA-7	2690	10	67	2.49	3.72	24.91	plants	82	7	49
S85-ssDNA-8	2994	15	106	3.54	5.01	35.40	plants	88	6	39
S86-ssDNA-9	5232	17	116	2.22	3.25	22.17	plants	74	8	39
S87-ssDNA-10	2861	15	99	3.46	5.24	34.60	plants	89	6	41
S88-ssDNA-11	1758	9	67	3.81	5.12	38.11	vertebrates	92	2	48
S89-ssDNA-12	2319	25	166	7.16	10.78	71.58	vertebrates	90	2	56
S90-ssDNA-13	3852	41	285	7.40	10.64	73.99	vertebrates	70	3	49
S91-ssDNA-14	8024	41	312	3.89	5.11	38.88	plants	59	8	39
S92-ssDNA-15	6396	35	250	3.91	5.47	39.09	plants	50	5	40
S93-ssDNA-16	5149	27	179	3.48	5.24	34.76	vertebrates	86	3	42
S94-ssDNA-17	5594	32	205	3.66	5.72	36.65	vertebrates	77	2	43
S95-ssDNA-18	4679	27	206	4.40	5.77	44.03	vertebrates	87	10	53
S96-ssDNA-19	4801	30	218	4.54	6.25	45.41	vertebrates	82	4	38
S97-ssDNA-20	5517	47	308	5.58	8.52	55.83	vertebrates	82	4	48
S98-ssDNA-21	5908	26	189	3.20	4.40	31.99	invertebrates	78	4	37
S99-ssDNA-22	5078	43	292	5.75	8.47	57.50	invertebrates	84	8	39
S100-ssDNA-23	3776	24	166	4.40	6.36	43.96	invertebrates	92	3	37
S101-ssDNA-24	5454	22	170	3.12	4.03	31.17	invertebrates	94	8	38
S102-dsDNA-RT-1	3215	11	72	2.24	3.42	22.40	vertebrates	100	4	49
S103-dsDNA-RT-2	3027	9	68	2.25	2.97	22.46	vertebrates	100	7	43
S104-dsDNA-RT-3	8024	30	208	2.59	3.74	25.92	plants	89	7	39
S105-dsDNA-RT-4	8178	48	301	3.68	5.87	36.81	plants	93	9	33
S106-dsDNA-RT-5	8159	75	507	6.21	9.19	62.14	plants	93	5	24
S107-dsDNA-RT-6	8002	41	270	3.37	5.12	33.74	plants	89	4	33
S108-dsDNA-RT-7	7489	18	129	1.72	2.40	17.23	plants	89	4	39
S109-dsDNA-RT-8	7206	27	176	2.44	3.75	24.42	plants	90	1	38
S110-ssRNA-RT-1	8805	32	214	2.43	3.63	24.30	vertebrates	91	7	43
S111-ssRNA-RT-2	8282	29	187	2.26	3.50	22.58	vertebrates	86	3	53
S112-ssRNA-RT-3	7286	31	202	2.77	4.25	27.72	vertebrates	65	11	53
S113-ssRNA-RT-4	8419	50	328	3.90	5.94	38.96	vertebrates	79	5	54
S114-ssRNA-RT-5	9181	65	440	4.79	7.08	47.93	vertebrates	93	9	42
S115-ssRNA-RT-6	12708	50	354	2.79	3.93	27.86	vertebrates	86	7	41
S116-ssRNA-RT-7	13246	53	362	2.73	4.00	27.33	vertebrates	78	6	38
S117-dsRNA-1	13385	38	260	1.94	2.84	19.42	bacteria	78	13	55
S118-dsRNA-2	23564	61	402	1.71	2.59	17.06	vertebrates	96	11	46
S119-dsRNA-3	19208	77	510	2.66	4.01	26.55	vertebrates	96	10	43
S120-dsRNA-4	17448	74	483	2.77	4.24	27.68	vertebrates	100	12	32
S121-dsRNA-5	29174	96	646	2.21	3.29	22.14	vertebrates	95	13	46
S122-dsRNA-6	23015	67	435	1.89	2.91	18.90	vertebrates	94	9	55

S123-dsRNA-7	24732	72	465	1.88	2.91	18.80	invertebrates	93	10	43
S124-dsRNA-8	29339	125	820	2.79	4.26	27.95	plants	94	12	31
S125-dsRNA-9	25709	76	514	2.00	2.96	19.99	plants	91	12	39
S126-dsRNA-10	26164	74	487	1.86	2.83	18.61	plants	92	11	44
S127-dsRNA-11	20682	45	311	1.50	2.18	15.04	vertebrates	86	12	39
S128-dsRNA-12	23433	59	394	1.68	2.52	16.81	fungi	93	11	41
S129-dsRNA-13	5881	21	136	2.31	3.57	23.13	invertebrates, vertebrates	93	3	54
S130-dsRNA-14	5898	13	84	1.42	2.20	14.24	invertebrates, vertebrates	96	3	53
S131-dsRNA-15	6603	18	113	1.71	2.73	17.11	invertebrates	92	2	46
S132-dsRNA-16	4579	11	70	1.53	2.40	15.29	fungi	98	3	45
S133-dsRNA-17	6277	21	142	2.26	3.35	22.62	protozoa	89	2	50
S134-dsRNA-18	5284	23	149	2.82	4.35	28.20	protozoa	94	3	46
S135-dsRNA-19	6105	25	181	2.96	4.10	29.65	fungi	91	2	39
S136-dsRNA-20	3663	11	74	2.02	3.00	20.20	plants	90	2	46
S137-dsRNA-21	12640	55	406	3.21	4.35	32.12	fungi	91	4	50
S138-dsRNA-22	12734	25	182	1.43	1.96	14.29	fungi	89	2	52
S139-dsRNA-23	17635	73	492	2.79	4.14	27.90	plants	99	1	47
S140-(-)ssRNA-1	8910	29	185	2.08	3.25	20.76	vertebrates	97	6	50
S141-(-)ssRNA-2	11161	47	301	2.70	4.21	26.97	invertebrates, vertebrates	95	5	41
S142-(-)ssRNA-3	11932	57	366	3.07	4.78	30.67	invertebrates, vertebrates	91	5	45
S143-(-)ssRNA-4	14900	87	592	3.97	5.84	39.73	invertebrates, vertebrates	95	11	33
S144-(-)ssRNA-5	12807	55	376	2.94	4.29	29.36	invertebrates, plants	90	8	42
S145-(-)ssRNA-6	12020	43	303	2.52	3.58	25.21	invertebrates, plants	95	7	46
S146-(-)ssRNA-7	11131	53	374	3.36	4.76	33.60	invertebrates, vertebrates	92	6	51
S147-(-)ssRNA-8	19111	63	392	2.05	3.30	20.51	vertebrates	76	7	38
S148-(-)ssRNA-9	18959	72	466	2.46	3.80	24.58	vertebrates	76	7	41
S149-(-)ssRNA-10	15384	47	302	1.96	3.06	19.63	vertebrates	94	9	46
S150-(-)ssRNA-11	15894	55	358	2.25	3.46	22.52	vertebrates	89	6	47
S151-(-)ssRNA-12	15384	60	371	2.41	3.90	24.12	vertebrates	92	7	42
S152-(-)ssRNA-13	18234	68	441	2.42	3.73	24.19	vertebrates	82	6	39
S153-(-)ssRNA-14	15186	63	401	2.64	4.15	26.41	vertebrates	90	6	46
S154-(-)ssRNA-15	15225	80	519	3.41	5.25	34.09	vertebrates	89	10	33
S155-(-)ssRNA-16	14071	81	533	3.79	5.76	37.88	vertebrates	92	9	41
S156-(-)ssRNA-17	12878	74	482	3.74	5.75	37.43	plants	83	6	45
S157-(-)ssRNA-18	11278	72	490	4.34	6.38	43.45	plants	94	4	34

S158-(-)ssRNA-19	13460	53	347	2.58	3.94	25.78	vertebrates	97	10	42
S159-(-)ssRNA-20	12555	56	360	2.87	4.46	28.67	vertebrates	98	9	37
S160-(-)ssRNA-21	10461	50	322	3.08	4.78	30.78	vertebrates	96	7	47
S161-(-)ssRNA-22	14452	47	318	2.20	3.25	22.00	vertebrates	94	11	40
S162-(-)ssRNA-23	12716	52	379	2.98	4.09	29.80	vertebrates	94	10	43
S163-(-)ssRNA-24	12294	66	452	3.68	5.37	36.77	vertebrates	95	4	35
S164-(-)ssRNA-25	11845	69	482	4.07	5.83	40.69	vertebrates	94	3	38
S165-(-)ssRNA-26	18859	87	568	3.01	4.61	30.12	vertebrates	96	3	40
S166-(-)ssRNA-27	11979	57	394	3.29	4.76	32.89	vertebrates	95	4	44
S167-(-)ssRNA-28	16634	106	698	4.20	6.37	41.96	plants	88	5	34
S168-(-)ssRNA-29	17145	96	624	3.64	5.60	36.40	plants	86	7	38
S169-(-)ssRNA-30	10056	29	180	1.79	2.88	17.90	vertebrates	97	4	42
S170-(-)ssRNA-31	1682	12	77	4.58	7.13	45.78	vertebrates	59	2	58
S171-(+)ssRNA-1	3569	8	48	1.34	2.24	13.45	bacteria	90	4	52
S172-(+)ssRNA-2	4215	8	56	1.33	1.90	13.29	bacteria	95	4	48
S173-(+)ssRNA-3	2514	7	49	1.95	2.78	19.49	fungi	99	1	58
S174-(+)ssRNA-4	2728	7	42	1.54	2.57	15.40	fungi	89	1	36
S175-(+)ssRNA-5	7440	21	139	1.87	2.82	18.68	vertebrates	89	1	46
S176-(+)ssRNA-6	7152	26	169	2.36	3.64	23.63	vertebrates	90	1	39
S177-(+)ssRNA-7	7478	22	144	1.93	2.94	19.26	vertebrates	89	3	37
S178-(+)ssRNA-8	7835	30	307	3.92	3.83	39.18	vertebrates	87	1	49
S179-(+)ssRNA-9	8161	28	196	2.40	3.43	24.02	vertebrates	85	1	53
S180-(+)ssRNA-10	7348	18	125	1.70	2.45	17.01	vertebrates	89	1	39
S181-(+)ssRNA-11	8828	20	127	1.44	2.27	14.39	vertebrates	88	1	48
S182-(+)ssRNA-12	8251	44	302	3.66	5.33	36.60	vertebrates	88	1	58
S183-(+)ssRNA-13	7117	20	134	1.88	2.81	18.83	vertebrates	94	1	44
S184-(+)ssRNA-14	9650	39	253	2.62	4.04	26.22	invertebrates	95	1	42
S185-(+)ssRNA-15	9185	36	243	2.65	3.92	26.46	invertebrates	87	2	39
S186-(+)ssRNA-16	8587	26	175	2.04	3.03	20.38	algae	90	1	46
S187-(+)ssRNA-17	9871	35	232	2.35	3.55	23.50	plants	92	1	43
S188-(+)ssRNA-18	12226	36	270	2.21	2.94	22.08	plants	85	1	45
S189-(+)ssRNA-19	12138	57	375	3.09	4.70	30.89	plants	90	2	46
S190-(+)ssRNA-20	10349	46	308	2.98	4.44	29.76	plants	93	7	43
S191-(+)ssRNA-21	9370	23	160	1.71	2.45	17.08	plants	94	4	42
S192-(+)ssRNA-22	9263	33	205	2.21	3.56	22.13	plants	92	2	43
S193-(+)ssRNA-23	11443	43	285	2.49	3.76	24.91	plants	89	2	46
S194-(+)ssRNA-24	9704	36	239	2.46	3.71	24.63	plants	94	1	42
S195-(+)ssRNA-25	9535	25	159	1.67	2.62	16.68	plants	97	1	46
S196-(+)ssRNA-26	11219	48	343	3.06	4.28	30.57	plants	88	2	46
S197-(+)ssRNA-27	10818	27	178	1.65	2.50	16.45	plants	95	1	42
S198-(+)ssRNA-28	9384	19	120	1.28	2.02	12.79	plants	97	1	44
S199-(+)ssRNA-29	8284	18	119	1.44	2.17	14.37	vertebrates	97	3	47
S200-(+)ssRNA-30	7437	17	105	1.41	2.29	14.12	vertebrates	99	2	50

S201-(+)ssRNA-31	7654	28	187	2.44	3.66	24.43	vertebrates	99	3	48
S202-(+)ssRNA-32	7476	23	152	2.03	3.08	20.33	vertebrates	98	2	51
S203-(+)ssRNA-33	7176	22	157	2.19	3.07	21.88	vertebrates	98	3	57
S204-(+)ssRNA-34	6813	25	166	2.44	3.67	24.37	vertebrates	97	2	44
S205-(+)ssRNA-35	7003	34	222	3.17	4.86	31.70	vertebrates	97	2	43
S206-(+)ssRNA-36	4540	4	27	0.59	0.88	5.95	invertebrates, vertebrates	95	3	54
S207-(+)ssRNA-37	4528	8	56	1.24	1.77	12.37	invertebrates, vertebrates	87	3	53
S208-(+)ssRNA-38	6625	20	141	2.13	3.02	21.28	invertebrates	87	3	54
S209-(+)ssRNA-39	4194	15	98	2.34	3.58	23.37	plants	95	4	51
S210-(+)ssRNA-40	5677	22	149	2.62	3.88	26.25	plants	86	7	48
S211-(+)ssRNA-41	5987	14	97	1.62	2.34	16.20	plants	91	6	49
S212-(+)ssRNA-42	5706	12	83	1.45	2.10	14.55	plants	89	5	49
S213-(+)ssRNA-43	4776	18	115	2.41	3.77	24.08	plants	87	5	48
S214-(+)ssRNA-44	4003	16	101	2.52	4.00	25.23	plants	91	6	48
S215-(+)ssRNA-45	3684	13	98	2.66	3.53	26.60	plants	96	5	48
S216-(+)ssRNA-46	5243	10	60	1.14	1.91	11.44	plants	87	4	47
S217-(+)ssRNA-47	4437	16	113	2.55	3.61	25.47	plants	89	5	50
S218-(+)ssRNA-48	4114	11	68	1.65	2.67	16.53	plants	88	4	50
S219-(+)ssRNA-49	4354	17	113	2.60	3.90	25.95	plants	93	5	47
S220-(+)ssRNA-50	4326	16	108	2.50	3.70	24.97	plants	91	6	50
S221-(+)ssRNA-51	12704	40	280	2.20	3.15	22.04	vertebrates	97	8	51
S222-(+)ssRNA-52	27608	90	597	2.16	3.26	21.62	vertebrates	95	6	37
S223-(+)ssRNA-53	28475	102	683	2.40	3.58	23.99	vertebrates	96	6	38
S224-(+)ssRNA-54	26253	111	754	2.87	4.23	28.72	vertebrates	97	N/A	46
S225-(+)ssRNA-55	10862	44	285	2.62	4.05	26.24	vertebrates	94	1	49
S226-(+)ssRNA-56	12573	60	402	3.20	4.77	31.97	vertebrates	95	1	45
S227-(+)ssRNA-57	9646	40	333	3.45	4.15	34.52	vertebrates	93	2	58
S228-(+)ssRNA-58	11703	31	208	1.78	2.65	17.77	vertebrates	96	3	50
S229-(+)ssRNA-59	9755	71	485	4.97	7.28	49.72	vertebrates	99	2	69
S230-(+)ssRNA-60	6395	32	208	3.25	5.00	32.53	plants	95	6	43
S231-(+)ssRNA-61	10646	41	276	2.59	3.85	25.93	plants	83	7	42
S232-(+)ssRNA-62	10221	28	240	2.35	2.74	23.48	plants	87	8	42
S233-(+)ssRNA-63	10692	43	309	2.89	4.02	28.90	plants	86	6	43
S234-(+)ssRNA-64	12141	54	369	3.04	4.45	30.39	plants	84	8	42
S235-(+)ssRNA-65	10401	55	373	3.59	5.29	35.86	plants	87	9	42
S236-(+)ssRNA-66	15914	55	410	2.58	3.46	25.76	plants	80	10	40
S237-(+)ssRNA-67	8274	24	155	1.87	2.90	18.73	plants	88	4	42
S238-(+)ssRNA-68	8622	30	188	2.18	3.48	21.80	plants	88	5	43
S239-(+)ssRNA-69	8210	33	233	2.84	4.02	28.38	plants	83	4	46
S240-(+)ssRNA-70	8623	31	212	2.46	3.60	24.59	plants	82	5	46
S241-(+)ssRNA-71	8301	26	181	2.18	3.13	21.80	plants	80	4	48

S242-(+)ssRNA-72	4852	11	79	1.63	2.27	16.28	plants	83	3	51
S243-(+)ssRNA-73	7680	26	167	2.17	3.39	21.74	plants	91	3	42
S244-(+)ssRNA-74	7564	54	413	5.46	7.14	54.60	plants	92	4	66
S245-(+)ssRNA-75	6318	19	125	1.98	3.01	19.78	plants	96	3	56
S246-(+)ssRNA-76	6305	19	124	1.97	3.01	19.67	plants	96	2	61
S247-(+)ssRNA-77	15480	50	329	2.13	3.23	21.25	plants	97	8	46
S248-(+)ssRNA-78	15311	55	365	2.38	3.59	23.84	plants	90	7	36
S249-(+)ssRNA-79	17919	58	376	2.10	3.24	20.98	plants	89	12	46
S250-(+)ssRNA-80	6435	18	120	1.86	2.80	18.65	plants	96	5	46
S251-(+)ssRNA-81	7560	37	247	3.27	4.89	32.67	plants	98	6	51
S252-(+)ssRNA-82	8832	22	140	1.59	2.49	15.85	plants	96	6	49
S253-(+)ssRNA-83	9306	29	191	2.05	3.12	20.52	plants	96	5	43
S254-(+)ssRNA-84	6495	23	150	2.31	3.54	23.09	plants	97	2	41
S255-(+)ssRNA-85	7351	17	112	1.52	2.31	15.24	plants	97	5	49
S256-(+)ssRNA-86	7555	31	200	2.65	4.10	26.47	plants	95	3	41
S257-(+)ssRNA-87	4009	9	64	1.60	2.24	15.96	fungi	91	4	46
